# *In situ* imaging and proteome profiling indicate andrographolide is a highly promiscuous compound

**DOI:** 10.1038/srep11522

**Published:** 2015-06-24

**Authors:** Lin Li, Hadhi Wijaya, Sanjay Samanta, Yulin Lam, Shao Q. Yao

**Affiliations:** 1 Department of Chemistry, National University of Singapore, Singapore 117543; 2 Key Laboratory of Flexible Electronics (KLOFE) & Institute of Advanced Materials (IAM), National Jiangsu Synergistic Innovation Center for Advanced Materials (SICAM), Nanjing Tech University (NanjingTech), Nanjing 211816, P. R. China

## Abstract

Natural products represent an enormous source of pharmacologically useful compounds, and are often used as the starting point in modern drug discovery. Many biologically interesting natural products are however not being pursued as potential drug candidates, partly due to a lack of well-defined mechanism-of-action. Traditional *in vitro* methods for target identification of natural products based on affinity protein enrichment from crude cellular lysates cannot faithfully recapitulate protein-drug interactions in living cells. Reported herein are dual-purpose probes inspired by the natural product andrographolide, capable of both reaction-based, real-time bioimaging and *in situ* proteome profiling/target identification in live mammalian cells. Our results confirm that andrographolide is a highly promiscuous compound and engaged in covalent interactions with numerous previously unknown cellular targets in cell type-specific manner. We caution its potential therapeutic effects should be further investigated in detail.

Natural products represent an enormous source of pharmacologically useful compounds^1^. They are the active components of many traditional medicines, and often used as the starting point in modern drug discovery^2^. Approximately 50% of FDA-approved drugs are directly derived from or inspired by natural products. Many biologically interesting natural products, however, have not been pursued as potential drug candidates due to a variety of reasons, including accessibility, cost, structural complexity, and a lack of well-defined mechanism-of-action^3^. Traditionally, *in vitro* methods based on affinity protein enrichment from crude cellular lysates are used to identify potential targets of natural products, but they are highly limited and cannot recapitulate protein-small molecule interactions *in situ* (e.g., in living cells)^4^. Consequently, wrong cellular targets might be identified^5^. Therefore, the field of natural product drug discovery can clearly benefit from innovative chemical tools capable of proteome-wide target identification under native cellular environments^6^. Inspired by concepts developed in activity-based protein profiling (ABPP)^7^, we recently introduced the so-called “*in situ* drug profiling“ approach in which protein-small molecule interactions were directly interrogated in living cells by using cell-permeable probes minimally modified from their parental bioactive compounds^8^,^9^, The strategy is applicable to compounds that form either irreversible or reversible complexes with their cellular targets, and has been adopted by others for both on- and off-target studies^10^,^11^,^12^,^13^,^14^,^15^. Few natural products, however, have been studied in this way, partly due to limited synthetic accessibility^6^,^15^. More recently, dual-purpose small molecule probes capable of both *in situ* imaging and target identification have been developed^16^; by combining information obtained from both the sub-cellular localization and large-scale cell-based proteome profiling of such probes, high-confidence cellular targets of bioactive compounds could be delineated. Notwithstanding, since the imaging capability of such probes was driven by non-covalent interaction with their intended cellular targets, they fell short of reporting real-time target engagement. Bogyo and co-workers developed fluorescently quenched activity-based probes based on peptide acyloxymethylketones which were capable of real-time imaging of protease activities in mammalian cells^17^. Herein, by successfully uniting natural product drug discovery, *in situ* drug profiling and real-time bioimaging of target-drug interaction for the first time, we report dual-purpose probes based on andrographolide (a natural product; Fig. 1). Detailed *in situ* bioimaging and proteome profiling confirmed that andrographolide is a highly promiscuous compound engaged in covalent interactions with numerous previously unidentified cellular targets in cell type-specific manner.

Andrographolide (henceforth referred to as **WT**) is a bicyclic diterpenoid isolated from leaves of *Andrographis paniculata*^18^, which is used extensively in traditional Chinese medicine (TCM). It possesses a wide spectrum of biological activities including antibacterial, anti-inflammatory, antimalarial, anticancer and others^19^, and is currently used in clinic in China for the treatment of paediatric pneumonia and respiratory tract infection^20^. The compound contains an α-alkylidene γ-butyrolactone moiety, two olefinic C-8/17 and C-12/13 bonds and three hydroxyls at C-14/C-3/C-19 positions. Like many known natural products that possess electrophilic moieties^15^, the α,β-unsaturated ester in **WT** is essential in maintaining the compound’s cellular activities by engaging in covalent reactions with its targets^19^. Previous structure-activity relationship (SAR) studies show attachment of potential leaving groups at C-14 OH of **WT**, via an ester or ether linkage, could improve its cellular potency, presumably due to facilitated nucleophilic attack by the target protein (Fig. 1)^19^,^21^. In our probe design (Fig. 2 & Table 1), we took advantage of this key feature by linking fluorogenic dyes of one- and two-photon properties (4-methyl-7-aminocoumarin or **AMC**, and 6-acetyl-2-naphthylamine or **AAN**) via a self-immolative carbamate^22^, giving probes **APCM**/**AP1CM**/**AP2CM** and **APNP**/**AP1NP**/**AP2NP**, respectively. Michael addition at the C-12 position of these probes by a nucleophilic residue (i.e. cysteine) from the intended target would result in the formation of a covalent protein-probe adduct, which then undergoes rapid and spontaneous β-elimination to release the highly fluorescent **AMC** and **AAN** (Fig. 2 & Supplementary Information). Since the Turn-ON fluorescence effect is position-specific and reaction-driven^23^, these probes are ideally suited for accurate, real-time reporting of target-probe interaction in living cells. SAR studies indicate modifications at C-3/C-19 OHs of **WT** were less predictable, but in many cases resulted in analogues that retained most of **WT**’s biological activities^19^,^24^. We therefore introduced 5-hexynoic acid to C-19 and C-3, via an ester linkage, providing the corresponding **AP1**/**AP1CM**/**AP1NP** and **AP2**/**AP2CM**/**AP2NP**, respectively. Molecular “tagging” of **WT** with such a clickable “minimalist” linker was not expected to adversely affect its intended cellular activities and target recognition^6^,^12^,^25^, and at the same time allowed *in situ* proteome profiling and subsequent *in vitro* affinity protein enrichment/target identification via the use of Cu(I)-catalyzed azide-alkyne cycloaddition (CuAAC)^26^. **WT** was the natural product of choice in our dual-purpose probe design, not only because of its unique chemical structure/reactivity, diverse biological activities and easy access via semi-synthesis^21^,^24^, but also of the ongoing controversy surrounding its true cellular targets. Several reports have indicated that, in addition to intracellular glutathione (GSH)^27^, and p50 protein of the transcription factor complex NFκB^28^, **WT** might have other unknown cellular targets^12^,^18^,^19^,^29^,^30^. In total, 9 different probes of **WT** were designed for the current study (Table 1).

## Results

### Design and synthesis of dual-purpose probes based on WT

**Figure 2:**
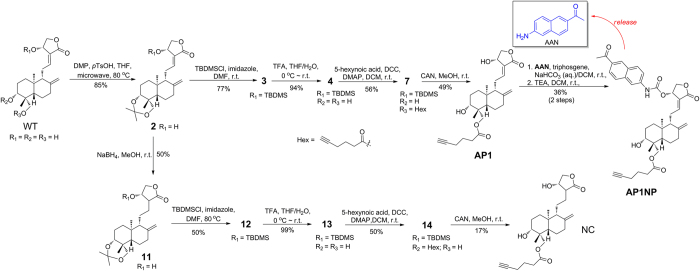
Chemical synthesis of dual-purpose probes from andrographolide. **AAN**: 2-acetyl-6-amino-napthalene; TBDMSCl: *tert*-butyldimethylsilyl chloride; *p*TsOH: *p*-toluenesulfonic acid; DMP: 2,2-dimethoxypropane; DCC: *N*,*N*’-dicyclohexylcarbodiimide; TEA: triethylamine; TFA: trifluoroacetic acid; DMAP: *N*,*N*-dimethyl-4-aminopyridine; CAN: cerium (IV) ammonium nitrate; r.t.: room temperature.

Synthetic access to these probes was significantly simplified by adopting semi-synthetic strategies based on previously reported procedures (Fig. 2 & Supplementary Information)^21^,^24^. **AP1** and **AP2** were obtained from the diol-protected key intermediate **2** in 4 and 5 steps, respectively. **AP3** was obtained in 2 steps from **2** in 40% overall yield, by using a modified Steglich esterification condition (**2** to **8**) followed by acetal deprotection (with AcOH/H_2_O). The synthesis of **APCM** and **APNP** was achieved by reacting **2** with the isocyanate of **ACM** and **AAN**, giving **8** and **9**, respectively, followed by acetal deprotection (with TFA/H_2_O). **AP1CM** and **AP1NP** were obtained by using similar methods from **AP1** in 65% and 36% yields, respectively. **AP2CM** and **AP2NP** were synthesised from **WT** through intermediate **18** by sequential protection of C-19 OH (giving **15**, 90%) and C-14 OH (giving **16**, 78%), followed by Steglich esterification with hexynoic acid (giving **17**, 94%) and THP removal with BF_3_^⊡^Et_2_O (73%). Subsequently, dye attachment followed by removal of protecting groups afforded **AP2CM** and **AP2NP** in 20% and 32% overall yields (3 steps). A negative probe, **NC**, which was structurally identical to **AP1** except the C-12/13 double bond was reduced, was synthesized from **2** by NaBH_4_ reduction (giving **11**, 50%), followed by steps of TBDMS protection of C-14 OH (giving **12**, 50%), acetal deprotection (giving **13**, 99%), Steglich esterification (giving **14**, 50%) and TBDMS deprotection (giving **NC**, 17%). All probes were fully characterized (see Supplementary Information).

### Photophysical and enzymatic propeties of probes

**Figure 3:**
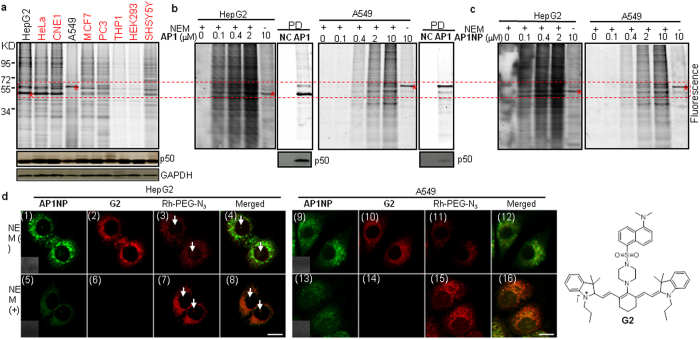
Proteome profiling and bioimaging. **a**, In-gel fluorescence scanning showing the proteome reactivity profiles of different cells labeled by **AP1** (10 μM, 3 h). WB results of p50 endogenous expression levels and GAPDH (loading control) are shown (bottom gels). **b**, In-gel fluorescence scanning showing concentration-dependent, *in situ* proteome profiles of HepG2/A549 cells pre-treated with NEM (1 mM, 20 min) followed by labeling with **AP1** (10 μM, 3 h), The corresponding pull-down (PD) gels of untreated HepG2/A549 cells labeled by **AP1** (10 μM, 3 h; **NC**: negative control) were shown. (Bottom) WB validation of the labeled p50 band. **c**, Same as **b** except **AP1NP** was used for labeling. (*) two cell type-specific bands at ~50 and 60 kDa. **d**, One-photon imaging of live HepG2 and A549 cells pre-treated with NEM (1 mM, 20 min) upon incubation with **AP1NP** (1 μM, 3 h) and **G2** (20 μM, 20 min). Cells were subsequently fixed and clicked with Rh-PEG-N_3_. λ_ex/em_ channels (**AP1NP**: 405/500–550 nm; **G2**: 635/680–780 nm; Rh-PEG-N_3_: 543/565–600 nm). (Merged) panels 1/3, 5/7, 9/11 & 13/15. (Insets) DIC images. Scale bar = 10 μm. (arrowed) putative p50-genereated, nuclear-localized fluorescence. Full size WB can be found in Supplementary Fig. S13.

We first evaluated the photochemical, photophysical and biochemical properties of these probes under physiological conditions (PBS buffer at *p*H 7.5, supplemented with 0.02% Triton X-100; Table 1 & Supplementary Table S1, Supplementary Fig. S1). **APNP**/**AP1NP**/**AP2NP** all had identical absorption maxima at 320 nm (ε = 7970/6540/6690 M^−1^cm^−1^), but different emission maxima for **APNP** (at 450 nm; Φ = 0.16) and **AP1NP**/**AP2NP** (at 485 nm; Φ = 0.08/0.03), which were blue-shifted when compared to the free dye **AAN** (λ_ex_/_em_ = 340/500 nm; ε = 7970 M^−1^cm^−1^, Φ = 0.34). For **APCM**/**AP1CM**/**AP2CM**, however, all three probes showed similar absorption and emission maxima (λ_ex_/_em_ = ~330/445 nm; ε = 7070/8500/9770 M^−1^cm^−1^, Φ = 0.51/0.17/0.08), which were similar to free dye **AMC** (λ_ex_/_em_ = 345/445 nm, ε = 9920 M^−1^cm^−1^). As expected, the brighter one- and two-photon excited fluorescence emission in **AAN** (ε.Φ = 2710, δ.Φ = 45 GM) and **AMC** (ε.Φ = 5059, δ.Φ = 14 GM), when compared to the corresponding probes **APNP**/**AP1NP**/**AP2NP** (ε.Φ = 1275/523/200, δ.Φ = 14/9.8/2.7 GM) and **APCM**/**AP1CM**/**AP2CM** (ε.Φ = 3606/1445/781, δ.Φ = 7.2/2.6/0.8 GM) at their excitation wavelengths, illustrates the anticipated Turn-ON fluorescence effect of the probes upon release of the free dye. Since the probes were expected to first react with intracellular GSH (>1 mM) upon cell uptake, we first investigated their reactivity/Turn-ON effect *in vitro* by reactions with exogenous GSH and HepG2 mammalian lysates^31^, with and without *N*-ethylmaleimide (NEM, a known GSH blocker^32^). Both time- and concentration-dependent fluorescence measurements were performed to obtain the reaction rates (Table 1 & Supplementary Fig. S1); without NEM, all dye-modified probes (**APNP/AP1NP/AP2NP/AP1CM/AP2CM**) except **APCM** produced significant Turn-ON fluorescence upon treatment with GSH and HepG2 lysates. The second-order rate constants of lysate/probe (*k*_HepG2_) were determined to be between 0.088 and 0.220 L^.^mg^−1^sec^−1^. NEM treatment caused nearly complete suppression of fluorescence increases, indicating they were a direct result of probe/GSH reaction. Compared to **AMC**-modified probes, **APNP/AP1NP/AP2NP** consistently produced more favorable Turn-ON fluorescence and faster kinetics. Since two-photon probes are ideally suited for live-cell/tissue imaging experiments^22^, they were chosen for subsequent bioimaging and *in situ* proteome profiling experiments (Fig. 3).

### Bioimaging and ***in situ*** proteome profiling

We first determined the cellular activities of these probes by XTT antiproliferation assay in HepG2 and A549 cancer cells (Supplementary Fig. S2). All probes showed similar activities as **WT** in inhibiting cell growth at micromolar concentrations. **NC** did not inhibit cell growth as expected. With the hexynoic acid-modified probes, **AP1**/**AP2**/**AP3**/**AP1NP**/**AP2NP**/**AP1CM**/**AP2CM**, additional *in situ* proteome profiling could be performed. Upon probe labeling, cell lysis and click chemistry with Rh-PEG-N_3_, cell lysates were further analyzed by SDS-PAGE and in-gel fluorescence scanning (Supplementary Fig. S3). Interestingly, by comparing the fluorescent labeling profiles (overall intensity and specificity), **AP1**/**AP1NP** consistently outperformed other probes. Surprisingly, distinctly different fluorescence profiles were observed between the two mammalian cell lines (see Fig. 3a–c & Supplementary Fig. S4; boxed in red), indicating the presence of cell type-specific probe targeting. Since our earlier *in vitro* results also showed **AP1**/**AP1NP** were the best-performing probes in Turn-ON fluorescence experiments (Table 1), they were chosen as the *de facto* dual-purpose set for subsequent imaging and proteome profiling experiments. We first surveyed different mammalian cells by *in situ* labeling with **AP1** at varied probe concentrations and incubation time (Fig. 3a & Supplementary Fig. S4); at 10 μM and 3 h incubation (conditions similar to most published protocols with **WT**^12^,^18^,^19^,^20^,^21^,^24^,^27^,^28^,^29^,^30^,^31^), all tested cell lines except THP1/HEK293 showed robust fluorescence profiles. We again observed a highly distinctly labeled band at ~60 kDa in A549 cells, but in most other cell lines including HepG2, the strongest labeled band was at ~50 kDa (* in Fig. 3a–c), which might be that of labeled p50 from NFκB^28^. Subsequent Western blotting (WB) analysis indicated endogenous p50 expression levels in all cell lines were similar. Competitive labeling with excessive **WT** (up to 10×) further confirmed the two highly cell type-specific 50-/60-kDa bands were indeed specific endogenous targets of **WT** (Supplementary Fig. S5). As the most abundant cellular target of **WT**, intracellular GSH would effectively quench most of **WT**/**AP1**/**AP1NP** upon their cellular uptake, rendering them inactive toward other cellular protein targets which might be more biologically relevant. We therefore developed an NEM-treatment protocol to effectively block intracellular GSH activities prior to subsequent *in situ* profiling and imaging experiments. A previously reported GSH-specific imaging probe **G2** was used to quantitatively monitor intracellular GSH levels (Fig. 3d)^32^. Pre-treatment of cells with NEM (1 mM, 20 min) completely abolished detectable intracellular GSH activities without causing significant cell death (Supplementary Fig. S6), and led to dramatic improvement of *in situ* proteome labeling profiles in both HepG2 and A549 cells (Fig. 3b); significantly more fluorescently labeled protein bands were detected even at lower **AP1** concentrations (0.1–2 μM). Upon click chemistry with Rh-Biotin-N_3_, the labeled lysates were subsequently pulled-down (PD) followed by WB analysis, which unequivocally confirmed the 50-kDa labeled band in HepG2 cells was indeed p50 of NFκB. In A549 cells, p50 was also positively, but much more weakly, labeled by **AP1** (bottom gels). Both **AP1** and **AP1NP** were shown to label cells similarly, with or without NEM treatment (Fig. 3c). **AP1NP** was thus used for subsequent reaction-driven, Turn-ON fluorescence imaging experiments in real-time (Fig. 3d & Supplementary video SI_IV).

**AP1NP** (a bifunctional **WT** mimic) was capable of real-time imaging of target/probe reaction through its C-14-linked **AAN** reporter (see Supplementary video). In addition, with the C-19-attached 5-hexynoic acid, it could provide information about the sub-cellular location of labeled protein targets (Fig. 3d), and when needed, be used for target identification by large-scale PD/LC-MS/MS experiments^8^,^9^,^16^. As a red-fluorescing GSH sensor, **G2** was used together with **AP1NP** for simultaneous two-color imaging. Again, cells were pre-treated with NEM to suppress intracellular GSH activities (Fig. 3d); without NEM treatment, intense fluorescence was observed in both **AP1NP** and **G2** channels (panels 1/2 & 9/10 for HepG2 & A549 cells, respectively). While signals from the **G2** channel were completely NEM-dependent (panels 6/14) and could be readily washed away, weaker **AAN** signals from the **AP1NP** channel persisted after NEM treatment (panels 5/13), indicating they were likely from covalent reactions between **AP1NP** and cellular protein targets. This was further confirmed by click chemistry of labeled cells with Rh-PEG-N_3_ followed by imaging (panels 3/7/11/15), with merged images of NEM-treated cells showing excellent overlaps between **AP1NP** and Rh-PEG-N_3_ channels (panels 8/16). During imaging, care was taken to ensure minimal diffusion of these **AAN** signals, which could also be removed by repeated washes of the cells. Significantly higher fluorescence signals from the Rh-PEG-N_3_ channel were detected in NEM-treated cells (compare panels 7/15 with 3/11), indicating blocking intracellular GSH activities had led to more effective labeling of endogenous cellular proteins, which is consist with our earlier *in situ* proteome profiling results (Fig. 3b,c). Of note, most fluorescence signals from both **AP1NP** and Rh-PEG-N_3_ channels were cytosolic, indicating the presence of unknown cellular targets in addition to the nuclear-localized p50 from NFκB (*vide infra*). Finally, to confirm **APNP**/**AP1NP**/**AP2NP** might be useful in deep-tissue imaging experiments with two-photon fluorescence microscopy (TPFM), two-day-old Drosophila brains were treated with **APNP** and successfully imaged with good resolution at a depth of 100 μm (Supplementary Fig. S7). We thus conclude that these novel self-reporting, natural product-inspired imaging probes are capable of sensitive, real-time detection of **WT**’s cellular activities in live cells and tissues.

### Cellular Targets Validation

**Figure 4:**
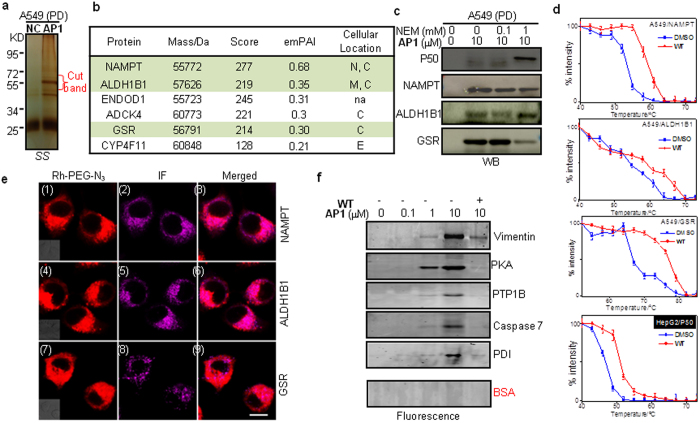
Targets validation. **a**, Silver-stained (SS) PD samples of *in situ* **AP1** (10 μM, 3 h)-labeled A549 cells. The cut band was subjected to LC-MS/MS analysis and results are summarized in **b**, Note: N, C, M and E represent nucleus, cytoplasm, mitochondrion and Endoplasmic reticulum, respectively. n/a = Not available. **c**, PD/WB target validation of A549 cells labeled by **AP1** (10 μM, 3 h) with or without NEM treatment. **d**, Graphical summary of CETSA results of A549 cells treated with **WT** (100 μM, 3 h). **WT**/p50 interaction in HepG2 cells were used as a positive control. **e**, Cell imaging of A549 cells labeled by **AP1** (10 μM, 3 h) followed by click chemistry with Rh-PEG-N_3_. IF = immunofluorescence (λ_ex/em_ = 630/650–750 nm). (Insets) DIC images. Scale bar = 10 μm. **f** Concentration-dependent *in vitro* labeling of recombination proteins (~50 ng) by **AP1** (1 h), with or without **WT** (100 μM). Full size WB can be found in Supplementary Fig. S13.

With a long history of remedial applications in TCM, *Andrographis paniculata* is currently a World Health Organization (WHO)-listed herb^33^. Its active ingredient, **WT**, despite having relatively poor cellular activities (in potency and selectivity), is being pursued by many research labs as a promising drug candidate^18^,^19^. We were intrigued by this natural product because most of its reported cellular and pharmacological properties point to the likelihood that it belongs to a group of molecules called pan-assay interference compounds (PAINS), which are highly promiscuous and appear active in many biological assays^34^. The *in situ* proteome profiling capability of **AP1** made it possible to test this hypothesis (Fig. 4). We were also interested to positively identify the 60-kDa labeled band, which appeared to be the most prominent cellular target of **WT** in A549 cells (a human lung adenocarcinoma epithelial cell line). Large-scale PD/LC-MS/MS experiments were performed with **AP1**-labeled cells and the gel slice at ~60-kDa region was cut, tryptically digested and analyzed (Fig. 4a,b); a total of six high-confidence candidate proteins were identified, and three cytosolic proteins were further confirmed to be true cellular targets of **WT** (Fig. 4c–e). NAMPT is a pleiotropic enzyme involved in a number of human diseases^35^. ALDH1B1 and GSR are two key proteins required to balance endogenous reactive oxygen species (ROS)^36^,^37^. PD/WB results indicate all three proteins were endogenously labeled in A549 cells even without NEM treatment. p50 on the other hand was positively labeled only in NEM-treated A549 cells. In HepG2 cells, they were labeled only after NEM treatment (Supplementary Fig. S10). In a cellular thermal shift assay (CETSA)^38^, all three proteins were efficiently stabilized by **WT** in A549 cells, indicating they were physically engaged in binding to **WT** in intake cells (Fig. 4d). p50 was similarly stabilized by **WT** in HepG2 cells. Cellular imaging of **AP1**-labeled A549 cells further indicates fluorescence signals from all three newly identified **WT** targets completely colocalized with those from the Rh-PEG-N_3_ channel (Fig. 4e, panels 3/6/9), but not *vice versa*, indicating there were additional endogenous **AP1** targets. Finally, 6 recombinant proteins known to possess nucleophilic cysteine residues were randomly chosen and labeled by **AP1** (Fig. 4f); results showed all but one were positively labeled at 1–10 μM (a concentration lower than most published **WT** protocols^18^,^19^), but negated in the presence of excess **WT**. These results thus unequivocally confirm **WT** is indeed a highly promiscuous compound.

## Discussion

In conclusion, the development of natural product-inspired dual-purpose probes has led to the successful reaction-based, real-time imaging of andrographolide activities in live mammalian cells and subsequent *in situ* proteome profiling and target identification. Our finding indicates andrographolide is a highly promiscuous compound, and engaged in covalent interactions with numerous previously unknown cellular targets at its pharmacologically relevant concentrations in cell type-specific manner. Because of this, the potential of these probes for live-cell imaging of specific cellular targets might be limited. The mechanism of cell type-specific targeting by andrographolide remains unknown as well, and is the subject of our ongoing investigation. Notwithstanding, we caution this bioactive compound should be further scrutinized before being seriously considered as a potential drug candidate.

## Methods

### Chemical synthesis

Information in detail was provided in Supporting Information.

### *In Situ* Proteome Profiling

These experiments were carried out mostly based on previously published protocols^8^,^9^, with necessary modifications as shown below. Briefly, cells were grown to >90% confluence in 6-well cell culture plates. After washing with PBS, cells were treated with different probes in 0.8 mL growth medium with a final DMSO concentration of <0.1%. Probes were stored in DMSO at −20 °C. **AP1** and other probes were added into cell medium and incubated for 3 h or other indicated time. After incubation, the medium was removed and cells were washed with PBS for three times to remove excessive probes. In competitive labeling experiments, cells were pre-incubated for 1 h at 37 °C with **WT** (1 ~ 10-fold excess) before addition of 10 μM probe. In NEM-competitive labeling experiments, cells were pre-incubated with NEM (1 mM or indicated concentrations) for 20 min at 37 °C, before addition of the probe (0 ~ 10 μM). After treatments, cells were detached and lysed in 300 μL PBS containing 0.02% Triton X-100. A freshly premixed click cocktail (4 eq. of Rh-PEG-N_3_, 4 eq. of TBTA (tris[(1-benzyl-1H-1,2,3-triazol-4-yl)methyl]amine), 40 eq. of TCEP (tris(2-carboxyethyl)phosphine) and 40 equivalents of CuSO_4_) was added. The reaction was incubated at room temperature for 2 h with gentle shaking before termination by addition of five-fold volumes of pre-chilled acetone. Incubation was continued overnight at −20 °C. Precipitated proteins were subsequently collected by centrifugation (13,000 rpm × 10 min at 4 °C). The supernatant was discarded and the residue pellet was washed with pre-chilled methanol, air-dried until the pellet started to shrink. Then the residue was resuspended in 1× standard SDS-loading buffer, sonicated for 10 min, heated for 10 min at 95 °C with gentle mixing, separated by SDS-PAGE, followed by in-gel fluorescence scanning and/or coomassie staining.

### General procedures for one- and two-photon fluorescence imaging of live cells and tissue

Cells were seeded in glass-bottom dishes (Mattek) and grown till 70 ~ 80% confluence. Subsequently, for imaging results shown in Fig. 3d & Supplementary Fig. S7, cells were incubated with **APNP/AP1NP/AP2NP** (1 or 10 μM in fresh growth medium) for 3 h. For experiments where **G2** was used to confirm successful blocking of in-cell GSH activitie^32^, **G2** was incubated with the cells for 20 min. Cells were next washed once with PBS, and then imaged with the Leica TCS SP5X Confocal Microscope System (panels 1/5/9/13 in Fig. 3d), as previously described^39^,^40^,^41^. **NC** or DMSO was used as imaging background controls (Supplementary Fig. 6 & 7). For imaging of cells pre-treated with NEM, cells were incubated with NEM (1 mM) for 20 min prior to addition of the probes. Subsequently, for probes-treated cells, they were fixed with 3.7% formaldehyde in PBS for 1 h at 37 °C in CO_2_, washed twice again and permeabilized with 0.02% Triton X-100 in PBS for 10 min at room temperature, washed twice with PBS again. Subsequently, cells were treated with a freshly premixed click chemistry reaction in 200 μL (1 eq. of Rh-PEG-N_3_, 2 eq. of TBTA, 20 eq. of TCEP, 20 eq. of CuSO_4_) for 1 h at room temperature with gentle shaking. Cells were washed with 2× PBS, several times PBS containing 0.02% Triton X-100 and 0.1 mM EDTA (until there was no small crystal under microscopy), 2× PBS, 2× methanol, 2× PBS. The cells were incubated with nucleus stain (Hoechst, 0.2 μg/mL final concentration) for 10 min at 37 °C and washed twice with PBS. Images were taken where indicated, as previously described^22^. For Drosophila 2P imaging, whole brains were prepared from a series of 2-day-old wild type Drosophila. Brains were incubated with **APNP** (50 μM) in DMEM under a humidified atmosphere of 5:95 (v/v) of CO_2_/air at 37 °C for 3 h. Treated brains were subsequently transferred to poly-L-lysine-coated coverslips. The images were taken at 100-μm depth by changing the z-axis thickness (Supplementary Fig. 8)^22^. For immunofluorescence of NAMPT, ALDH1B1 and GSR, **AP1**-treated A549 cells were fixed with 3.7% formaldehyde and permeabilized with 0.02% Triton X-100, blocked with 2% BSA in PBS for 30 min at room temperature, followed by incubation with primary antibody anti-NAMPT (1:200), anti-ALDH1B1 (1:500) and anti-GSR (1:500), respectively, for 4 h at room temperature or 4 °C overnight. Upon washing with PBST (with 0.02% Triton X-100) twice for 5 min each and incubation with Cy5-conjugated Goat anti-Rabbit IgG (1:1000) for 2 h at room temperature, the cells were then washed with PBST (with 0.02% Triton X-100) three times for 5 min each and washed with PBS for 5 min with gentle agitation and a final wash with deionized water (1 ~ 2 min with gentle agitation) before images were acquired (Fig. 4e). All images were processed with Leica Application Suite Advanced Fluorescence (LAS AF)^22^. A real-time imaging video clip (time: 0 to 3 h) of HepG2 cells upon treatment with **AP1NP** (10 μM; 3 h) are shown in SI_IV.

### Target Identification/Validation

For cellular thermal shift assay (CETSA), experiments were carried out similarly as published protocols^38^. A549 cells were seeded in 10 cm cell culture dishes and grown till ~90% confluence. Subsequently, the cells were incubated with **WT** (100 μM in fresh growth medium) for 3 h in the CO_2_ incubator at 37 °C. The same volume of DMSO was used to serve as negative control in a separate dish. The medium was subsequently aspirated, and cells were washed with PBS (10.0 mL), then harvested by scraping in fresh PBS (20.0 mL). Cell pellets were isolated by centrifugation (1200 rpm, 5 min, 4 °C), resuspended in 1.2 mL PBS and distributed into 12 different 1.5 mL tubes with 100 μL of cell suspension in each tube for both DMSO control and **WT**-treated cells. The tubes were heated at their designated temperature endpoints (40 ~ 73 or 52 ~ 85 °C) for 3 min on a heating block. Immediately after heating, the tubes were removed and incubated at room temperature for another 3 min. After that, cells were freeze-thawed twice by using liquid nitrogen and a heating block set at 25 °C in order to ensure a uniform temperature between tubes after keeping at −80 °C overnight. 50 μL of supernatant were collected by centrifugation (eppendorf centrifuge 5415R, 13, 000 rpm × 1 h) from each resulting cell lysate at 4 °C. Then the residue was mixed with 10 μL 6× standard SDS-loading buffer, sonicated for 10 min, heated for 10 min at 95 °C with gentle mixing, separated by SDS-PAGE, followed by transfer to a PVDF membrane for WB analysis (Supplementary Fig. S12).

### Other methods

Additional experimental information and results (chemistry and biology) are provided in Supplementary Methods.

## Additional Information

**How to cite this article**: Li, L. *et al*. *In situ* imaging and proteome profiling indicate andrographolide is a highly promiscuous compound. *Sci. Rep*. **5**, 11522; doi: 10.1038/srep11522 (2015).

## Supplementary Material

Supplementary Information

Supplementary Movie S1

## Figures and Tables

**Figure 1:**
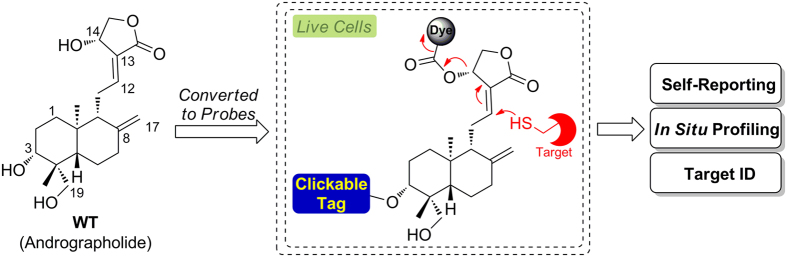
Overall strategy of dual-purpose probes for real-time imaging of target-drug interaction and *in situ* proteome profiling/target identification.

**Table 1:**
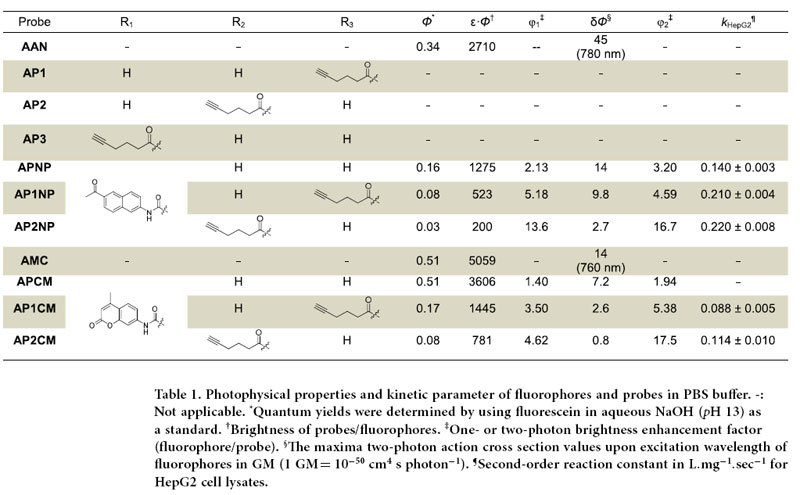
Photophysical properties and kinetic parameter of fluorophores and probes in PBS buffer.
