# Supplementary material for: In situ imaging and proteome profiling indicate andrographolide is a highly promiscuous compound
Source: Sci Rep. 2015 Jun 24;5:11522. doi: 10.1038/srep11522 (PMC4478469; doi:10.1038/srep11522)
Supplement: Supplementary Information [file srep11522-s1.pdf]

Supporting Information (I)

In situ imaging and proteome profiling indicate andrographolide is a highly promiscuous compound

Lin Li,[1,2] Hadhi Wijaya,[1] Sanjay Samanta,[1] Yulin Lam[1] and Shao Q. Yao[1*]*

[1]Department of Chemistry, National University of Singapore, Singapore 117543.

[2]Key Laboratory of Flexible Electronics (KLOFE) & Institute of Advanced Materials (IAM), National Jiangsu Synergistic Innovation Center for Advanced Materials (SICAM), Nanjing Tech University (NanjingTech), Nanjing, P. R. China 211816.

**Correspondence to Yulin Lam (chmlamyl@nus.edu.sg); Shao Q. Yao (chmyaosq@nus.edu.sg)*

1. General methods

All chemicals were purchased from commercial vendors and used without further purification, unless otherwise stated. *N,N'*-Dimethylformamide (DMF) and dichloromethane (CH_2Cl_2, DCM) were distilled over CaH_2. All non-aqueous reactions were carried out under nitrogen atmosphere in oven-dried glasswares. Reaction progress was monitored by TLC on pre-coated silica plates (Merck 60 F_{254}, 250 µm thickness) and spots were visualized by ceric ammonium molybdate, basic $KMnO_4$, UV light or iodine. Flash column chromatography was carried out using Merck silica gel (0.040-0.063). ^{1}H NMR and ^{13}C NMR spectra were recorded on a Bruker model DPX-500 MHz NMR spectrometer. Chemical shifts are reported in parts per million relative to internal standard tetramethylsilane ($Si(CH_3)_4$ = 0.00 ppm) or residual solvent peaks ($CDCl_3$ = 7.26 ppm, DMSO-d_6 = 2.50 ppm, MeOD = 3.31 ppm). ^{1}H NMR coupling constants (J) are reported in Hertz (Hz) and multiplicity is indicated as follows: s (singlet), d (doublet), t (triplet), q (quartet), m (multiplet), br s (broad singlet), br d (broad doublet), dd (doublet of doublet), dt (doublet of triplet), dq (doublet of quartet), tq (triplet of quartet). Mass spectra were recorded on a Finnigan LCQ mass spectrometer, a Shimadzu LC-IT-TOF spectrometer or a Shimadzu LC-ESI spectrometer. UV-vis absorption and fluorescence spectra were measured by using a Shimadzu UV-vis spectrophotometer and a Perkin Elmer LS50 spectrofluorometer, respectively. The two-photon excited fluorescence measurements were performed by using a Spectra Physics femtosecond Ti: sapphire oscillator (Tsunami) as the excitation source. The output laser pulses have a tunable center wavelength from 750 nm to 840 nm with pulse duration of 40 fs and a repetition rate of 76 MHz. The laser beam was focused onto the samples that were contained in a cuvette with path length of 1 cm. The emission from the samples was collected at 90 °angle by a pair of lenses and an optical fiber that was connected to a monochromator (Acton, Spectra Pro 2300i) coupled with CCD (Princeton Instruments, Pixis 100B) system. A short pass filter with cut-off wavelength at 700 nm was placed before the spectrometer to minimize the scattering from the pump beam. All images were acquired on Leica TCS SP5X Confocal Microscope System equipped with Leica HCX PL APO 63x/1.20 W CORR CS, as previously described[1-3]. Images were processed with Leica Application Suite Advanced Fluorescence (LAS AF). All the measurements were performed at room temperature.

2. Synthesis and Characterizations

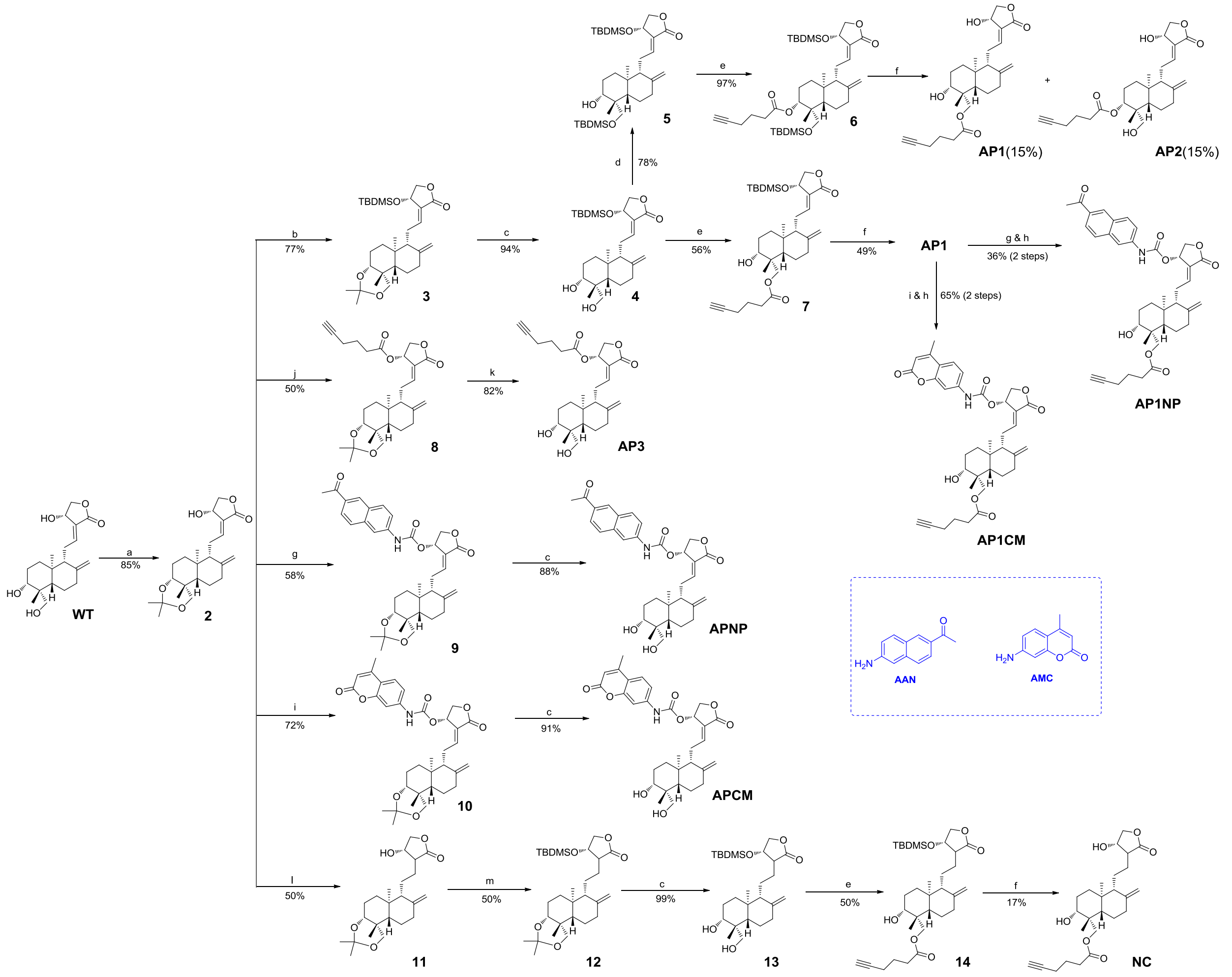

Scheme S1. Synthesis scheme of **AP1**/**AP2**/**AP3**/**NC**, **APNP**/**APCM** and **AP1NP**/**AP1CM** from **WT**. a) 2,2-dimethoxypropane, *p*-toluenesulfonic acid, THF, microwave, 80 °C, 10 min; b) *tert*-butyldimethylsilyl chloride (TBDMSCl), imidazole, DMF, r.t., 30 min; c) trifluoroacetic acid, THF/H_2O, 0 °C ~ r.t., 1 h; d) TBDMSCl, pyridine, r.t., 1 h; e) 5-hexynoic acid, *N,N'*-dicyclohexylcarbodiimide, *N,N*-dimethyl-4-aminopyridine, DCM, r.t., 10 h; f) cerium (IV) ammonium nitrate, MeOH, r.t., 20 h; g) 2-acetyl-6-amino-napthalene (**AAN**), triphosgene, saturated $NaHCO_3$ (aq.)/DCM, r.t., 30 min; h) triethylamine, DCM, r.t., 2.5 h; i) 7-amino-4-methylcoumarin (**AMC**), triphosgene, saturated $NaHCO_3$ (aq.)/DCM, r.t.; j) 5-hexynoic acid, *N,N'*-dicyclohexylcarbodiimide, DCM, r.t., 4 days; k) AcOH/H_2O, r.t., 30 min; l) $NaBH_4$, MeOH, r.t., 1 h; m) TBDMSCl, imidazole, DMF, 80 °C, 30 min. r.t. = room temperature.

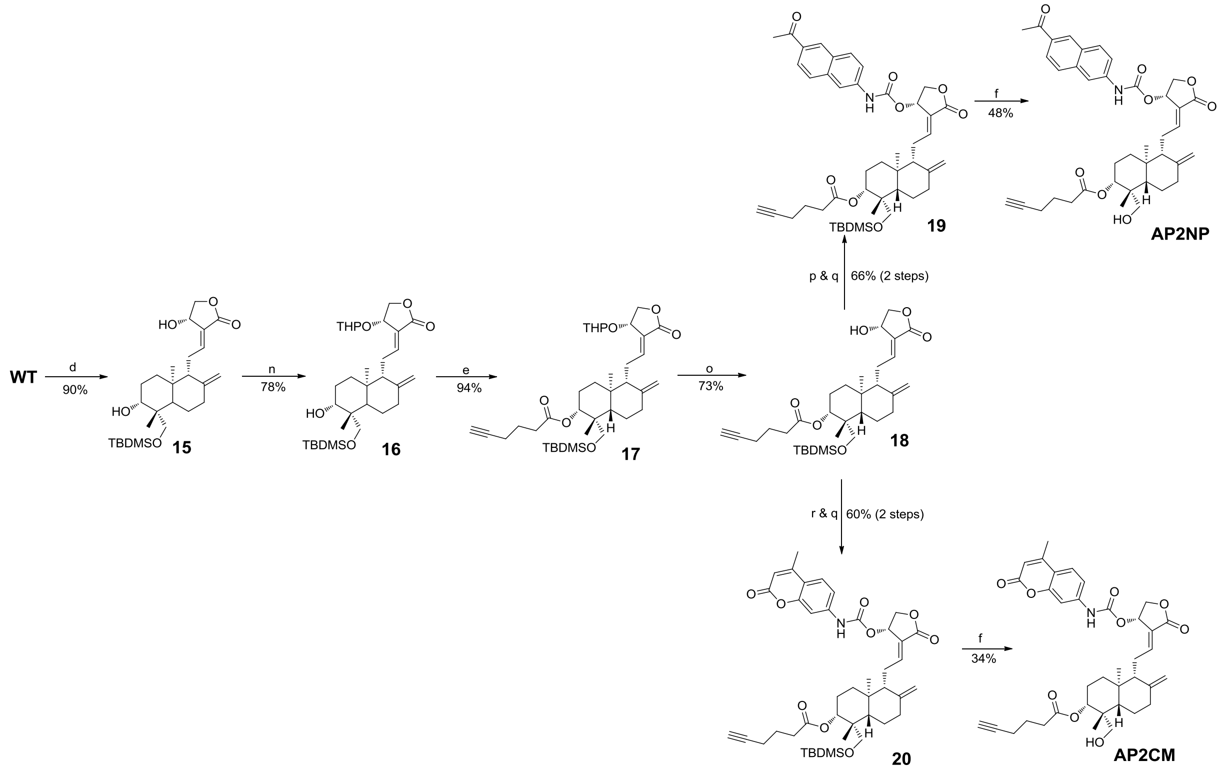

Scheme S2. Synthesis of **AP2NP** and **AP2CM.** d) TBDMSCl, r.t., pyridine, 1 h; e) 5-hexynoic acid, *N*,*N*'-dicyclohexylcarbodiimide, *N*,*N*-dimethyl-4-aminopyridine, DCM, r.t., 10 h; f) cerium (IV) ammonium nitrate, MeOH, r.t., 20 h; n) 3,4-dihydro-2H-pyran, *p*-toluenesulfonic acid, DCM, r.t., 40 min; o) BF_3•Et_2O, $HSCH_2CH_2SH$/DCM, -40 °C, 30 min; p) **AAN**, triphosgene, saturated $NaHCO_3$ (aq.)/DCM, -20 °C, 30 min; q) triethylamine, DCM, -20 °C, 2.5 h; r) **AMC**, triphosgene, saturated $NaHCO_3$ (aq.)/DCM, -20 °C, 30 min. r.t. = room temperature.

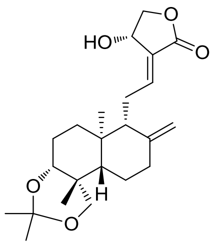

3,19-Isopropylidine androgapholide (**2**): A stirred mixture of andrographolide (**WT**) (0.5 g, 1.43 mmol), tetrahydrofuran (10 mL), *p*-toluenesulfonic acid (25 mg, 0.143 mmol) and 2,2-dimethoxypropane (3.51 mL, 28.53 mmol) was microwave irradiated at 80 ^{o}C for 10 min. The reaction mixture was then concentrated and purified by flash chromatography (ethyl acetate/hexane = 1/3 to 1/2) to yield **2** as a white solid (Yield: 85%). ^{1}H NMR (500 MHz, $CDCl_3$) 6.92 (t, *J* = 6.5 Hz, 1H), 5.01 (t, *J* = 5.7 Hz, 1H), 4.89 (s, 1H), 4.61 (s, 1H), 4.43 (dd, *J* = 10.4, 6.1 Hz, 1H), 4.24 (dd, *J* = 10.4, 1.6 Hz, 1H), 3.95 (d, *J* = 11.6 Hz, 1H), 3.48 (dd, *J* = 8.6, 3.4 Hz, 1H), 3.17 (d, *J* = 11.6 Hz, 1H), 2.98 (d, *J* = 7.1 Hz, 1H), 2.56 (t, *J* = 6.5 Hz, 2H), 2.41 (d, *J* = 13.5 Hz, 1H), 2.01 ~ 1.93 (m, 2H), 1.87 ~ 1.67 (m, 4H), 1.40 (s, 3H), 1.35 (s, 3H), 1.32 ~ 1.22 (m, 3H), 1.19 (s, 3H), 0.94 (s, 3H). ^{13}C NMR (125 MHz, $CDCl_3$) 170.29, 149.05, 146.93, 127.94, 108.91, 99.11, 76.23, 74.44, 66.01, 63.83, 55.95, 52.17, 38.37, 37.84, 37.60, 34.50, 27.04, 26.07, 25.28, 24.95, 24.93, 23.14, 16.12. HRMS (FAB, $[M+H]^+$): calcd. for $C_{23}H_{35}O_5$: 391.2479; found 391.2495.

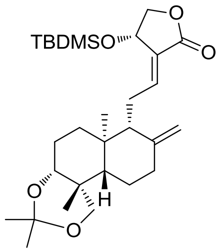

3,19-Isopropylidine-14-*t*-butyldimethylsily andrographolide (3): To a solution of **2** (50 mg, 0.13 mmol) in anhydrous DMF (0.2 mL) were added imidazole (44 mg, 0.64 mmol) and *t*-butyldimethylsilyl chloride (TBDMSCl, 48 mg, 0.32 mmol). The resulting mixture was stirred at room temperature for 30 min. The reaction mixture was then diluted with diethyl ether and washed with water (2×) and brine (2×). The organic layer was dried over anhydrous $MgSO_4$, filtered and the crude mixture was purified by flash chromatography (ethyl acetate/hexane = 1/5 to 1/4) to afford **3** as a white solid (Yield: 77%). ^{1}H NMR (500 MHz, $CDCl_3$) 6.83 (t, *J* = 6.3 Hz, 1H), 5.05 (s, 1H), 4.87 (s, 1H), 4.59 (s, 1H), 4.41 (dd, *J* = 9.8, 6.4 Hz, 1H), 4.08 (dd, *J* = 9.8, 2.9

Hz, 1H), 3.95 (d, *J* = 11.6 Hz, 1H), 3.50 (dd, *J* = 8.2, 3.8 Hz, 1H), 3.17 (d, *J* = 11.6 Hz, 1H), 2.63 ~ 2.56 (m, 1H), 2.44 ~ 2.39 (m, 2H), 1.98 ~ 1.94 (m, 2H), 1.85 (d, *J* = 11.2 Hz, 1H), 1.82 ~ 1.77 (m, 1H), 1.69 ~ 1.66 (m, 2H), 1.41 (s, 3H), 1.36 (s, 3H), 1.29 ~ 1.25 (m, 3H), 1.19 (s, 3H), 0.95 (s, 3H), 0.91 (s, 9H), 0.16 (s, 3H), 0.11 (s, 3H). ^{13}C NMR (125 MHz, $CDCl_3$) 169.94, 148.19, 146.71, 127.38, 109.73, 99.20, 75.94, 73.91, 66.95, 63.99, 56.09, 51.91, 38.16, 37.95, 37.54, 34.48, 26.82, 26.07, 25.59, 25.21, 24.83, 24.79, 23.10, 17.81, 16.51, -4.21, -4.67. HRMS (ESI, M + Na): calcd. for $C_{29}H_{48}NaO_5Si$, 527.3169; found 527.3177.

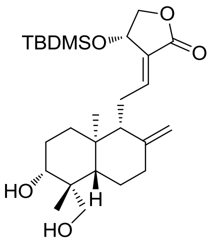

14-*t*-Butyldimethylsilyl andrographolide (**4**): To a solution of **3** (80 mg, 0.16 mmol) in THF/H_2O (4/1, 0.84 mL) at 0 ^{o}C was added trifluoroacetic acid (29 μL, 0.38 mmol). The resulting mixture was then warmed up to room temperature and stirred for 1 h. Thereafter, saturated sodium bicarbonate was added to neutralize the acid. The aqueous layer was then extracted with ethyl acetate (4×) and the combined organic layer was dried over anhydrous $MgSO_4$, filtered and concentrated to yield **4** as a white solid (Yield: 94%). ^{1}H NMR (500 MHz, $CDCl_3$) 6.78 (t, *J* = 5.7 Hz, 1H), 5.03 (s, 1H), 4.83 (s, 1H), 4.54 (s, 1H), 4.40 (dd, *J* = 9.7, 6.4 Hz, 1H), 4.16 (d, *J* = 11.1 Hz, 1H), 4.06 (dd, *J* = 9.8, 2.9 Hz, 1H), 3.47 ~ 3.44 (m, 1H), 3.31 (d, *J* = 11.0 Hz, 1H), 2.57 ~ 2.50 (m 1H), 2.38 (d, *J* = 13.6 Hz, 2H), 1.95 (t, *J* = 9.9 Hz, 1H), 1.83 ~ 1.78 (m, 4H), 1.68 (d, *J* = 13.0 Hz, 1H), 1.30 ~ 1.17 (m, 6H), 0.89 (s, 9H), 0.65 (s, 3H), 0.14 (s, 3H), 0.10 (s, 3H). ^{13}C NMR (125 MHz, $CDCl_3$) 170.05, 148.24, 146.32, 127.22, 109.67, 80.23, 73.93, 66.90, 63.98, 55.95, 55.06, 42.67, 38.59, 37.58, 37.12, 27.96, 25.54, 24.64, 23.56, 22.68, 17.75, 15.32, -4.27, -4.74. HRMS (ESI, $[M-H]^-$) calcd. for $C_{26}H_{43}O_5Si$: 463.2885; found 463.2890.

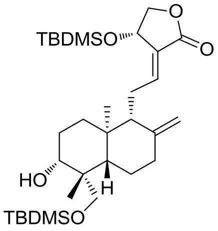

14,19-Di-*t*-butyldimethylsilyl andrographolide (**5**): To a solution of **4** (852 mg, 1.80 mmol) in pyridine (13 mL) was added *t*-butyldimethylsilyl chloride (3.20 g, 21 mmol). The resulting mixture was stirred at room temperature for 1 h. Ethyl acetate was then added to dilute the reaction mixture and the organic layer was subsequently washed with 1 M copper sulfate solution (4×), dried over $MgSO_4$, filtered and concentrated. The crude mixture was purified by flash chromatography (ethyl acetate/hexane = 1/5 to 1/4) to afford **5** as a white solid (Yield: 78%). ^{1}H NMR (500 MHz, $CDCl_3$) 6.83 ~ 6.79 (m, 1H), 5.05 ~ 5.04 (m, 1H), 4.84 (s, 1H), 4.55 (s, 1H), 4.41 (dd, *J* = 9.8, 6.4 Hz, 1H), 4.34 (d, *J* = 7.2 Hz, 1H), 4.19 (d, *J* = 10.0 Hz, 1H), 4.07 (dd, *J* = 9.8, 3.0 Hz, 1H), 3.38 (d, *J* = 10.0 Hz, 1H), 3.34 ~ 3.28 (m, 1H), 2.57 ~ 2.50 (m, 1H), 2.43 ~ 2.36 (m, 2H),1.99 ~ 1.79 (m, 4H), 1.74 ~ 1.65 (m, 3H), 1.28 ~ 1.25 (m 2H), 1.22 (s, 3H), 0.90 (s, 9H), 0.88 (s, 9H), 0.68 (s, 3H), 0.15 (s, 3H), 0.11 (s, 3H), 0.06 (s, 3H), 0.05 (s, 3H). ^{13}C NMR (125 MHz, $CDCl_3$) 169.89, 148.07, 146.36, 127.31, 109.59, 80.04, 73.88, 66.96, 65.18, 56.10, 55.13, 42.48, 38.72, 37.66, 37.31, 28.57, 25.75, 25.59, 24.55, 23.72, 23.08, 18.05, 17.79, 15.71, -4.21, -4.71, -5.78, -5.83. HRMS (ESI, $[M+H]^+$) calcd. for $C_{32}H_{59}O_5Si_2$: 579.3896; found 579.3913.

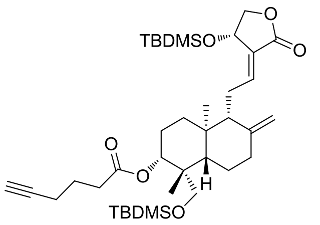

14,19-Di-*t*-butyldimethylsilyl 3-hexynoate andrographolide (**6**): To a solution of **5** (300 mg, 0.5 mmol) in dichloromethane (5 mL) were added 5-hexynoic acid (192 μL, 1.55 mmol), coupling reagent *N,N'*-dicyclohexylcarbodiimide (352 mg, 1.71 mmol) and *N,N*-dimethyl-4-aminopyridine (19 mg, 0.16 mmol). The resulting mixture was stirred at room temperature for 10 h. The suspension was then filtered and the crude mixture was purified by flash chromatography (ethyl acetate/hexane = 1/10 to 1/8) to afford **6** as a white solid (Yield: 97%). ^{1}H NMR (500 MHz, $CDCl_3$) 6.85 ~ 6.82 (m, 1H), 5.04 ~ 5.03 (m, 1H), 4.86 (s, 1H), 4.59 (dd, *J* = 10.8, 5.3 Hz, 1H), 4.55 (s, 1H), 4.40 (dd, *J* = 9.8, 6.3 Hz, 1H), 4.08 (m, 1H), 3.83 (d, *J* = 10.5 Hz, 1H), 3.61 (d, *J* = 10.5 Hz, 1H), 2.58 ~ 2.51 (ddd, *J* = 17.9, 11.4, 7.0 Hz, 1H), 2.44 (td, *J* = 7.4, 2.1 Hz, 2H), 2.39 ~ 2.36 (m, 1H), 2.25 (td, *J* = 6.9, 2.6 Hz, 2H), 1.96 (t, *J* = 2.6 Hz, 1H), 1.93 ~ 1.61 (m, 10H), 1.35 ~ 1.25 (m, 2H), 0.94 (s, 3H), 0.90 (s, 9H), 0.88 (s, 9H), 0.80 (s, 3H), 0.15 (s, 3H), 0.11 (s,

3H), 0.02 (s, 6H). ^{13}C NMR (125 MHz, $CDCl_3$) 172.64, 169.89, 148.10, 146.80, 127.35, 109.19, 83.15, 80.15, 73.89, 69.10, 66.95, 63.65, 56.18, 55.41, 42.48, 38.87, 38.16, 37.51, 33.25, 25.86, 25.58, 25.24, 24.59, 24.36, 23.72, 23.18, 18.22, 17.83, 17.80, 14.55, -4.21, -4.70, -5.64, -5.72. HRMS (ESI, $[M+Na]^+$) calcd. for $C_{38}H_{64}NaO_6Si_2$: 695.4134; found 695.4164.

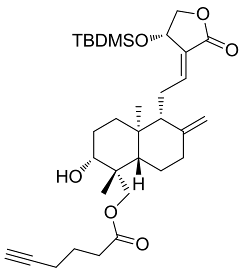

Compound (**7**): To a solution of **4** (1.44 g, 3.1 mmol) in dichloromethane (25 mL) was added 5-hexynoic acid (351 μL, 3.1 mmol), coupling reagent *N,N'*-dicyclohexylcarbodiimide (831 mg, 4.03 mmol) and *N*,*N*-dimethyl-4-aminopyridine (113 mg, 0.92 mmol). The resulting mixture was stirred at room temperature for 10 h. The suspension was then filtered and the crude mixture was purified by flash chromatography (ethyl acetate/hexane = 3/7 to 2/3) to afford **7** as a white solid (Yield: 56%). ^{1}H NMR (500 MHz, $CDCl_3$) δ 6.81 (t, J = 5.6 Hz, 1H), 5.10 ~ 4.99 (m, 1H), 4.88 (s, 1H), 4.67 (dd, J = 11.5, 4.3 Hz, 1H), 4.58 (s, 1H), 4.41 (dd, J = 9.7, 6.4 Hz, 1H), 4.13 (d, J = 11.7 Hz, 1H), 4.08 (dd, J = 9.8, 3.0 Hz, 1H), 3.38 (t, J = 11.1 Hz, 1H), 2.60 ~ 2.34 (m, 4H), 2.27 (m, 2H), 2.05 ~ 1.92 (m, 3H), 1.89 ~ 1.69 (m, 6H), 1.46 ~ 1.27 (m, 3H), 1.08 (s, 3H), 0.90 (d, J = 6.4 Hz, 9H), 0.70 (s, 3H), 0.14 (s, 3H), 0.11 (s, 3H). ^{13}C NMR (125 MHz, $CDCl_3$) 172.26, 170.16, 148.07, 146.31, 127.74, 110.24, 83.12, 82.66, 74.16, 69.80, 67.27, 63.77, 56.12, 55.68, 42.90, 38.88, 37.85, 37.33, 33.37, 25.87, 24.86, 24.49, 24.10, 23.81, 22.71, 18.09, 17.97, 15.42, -3.92, -4.40. HRMS (ESI, $[M-H]^-$) calcd. for $C_{32}H_{49}O_6Si$: 557.3304; found 557.3285.

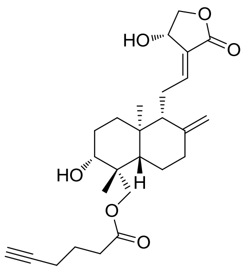

19-Hexynoate andrographolide (**AP1**): To a solution of **6** (20 mg, 0.03 mmol) in methanol (0.2 mL) was added cerium (IV) ammonium nitrate (20 mg, 0.036 mmol). The resulting mixture was stirred at room temperature for 20 h. Thereafter, the crude reaction mixture was purified by flash

chromatography (ethyl acetate/hexane = 1/1 to 2/1) to afford **AP1** as a white solid (Yield: 15%). Alternatively, **AP1** could be obtained from the **7** (Yield: 49%). ^{1}H NMR (500 MHz, $CDCl_3$) 6.95 (t, *J* = 6.7 Hz, 1H), 5.03 (d, *J* = 5.7 Hz, 1H), 4.91 (s, 1H), 4.60 (s, 1H), 4.46 (dd, *J* = 10.5, 6.1 Hz, 1H), 4.36 (d, *J* = 11.7 Hz, 1H), 4.26 (dd, *J* = 10.3, 1.6 Hz, 1H), 4.12 (d, *J* = 11.6 Hz, 1H), 3.33 (dd, *J* = 11.9, 4.0 Hz, 1H), 2.55 (t, *J* = 6.7 Hz, 2H), 2.47 ~ 2.43 (m, 3H), 2.27 (dt, *J* = 6.9, 3.4 Hz, 2H), 2.00 ~ 1.95 (m, 2H), 1.86 ~ 1.79 (m, 6H), 1.74 ~ 1.69 (m, 1H), 1.46 (dd, *J* = 13.0, 4.1 Hz, 1H), 1.29 ~ 1.23 (m, 4H), 1.16 (s, 3H), 0.73 (s, 3H). ^{13}C NMR (125 MHz, $CDCl_3$) 173.04, 169.78, 148.75, 146.46, 128.01, 108.91, 83.10, 78.76, 74.23, 69.27, 66.24, 64.82, 55.96, 55.30, 42.51, 39.06, 37.77, 37.28, 33.00, 27.75, 24.77, 24.24, 23.51, 22.49, 17.79, 14.80. HRMS (ESI, $[M-H]^-$) calcd. for $C_{26}H_{35}O_6$: 443.2439; found 443.2421.

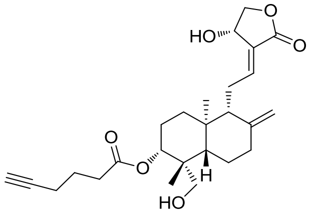

3-Hexynoate andrographolide (**AP2**): The crude reaction mixture in same reaction of **AP1** from **6** was subsequently purified by flash chromatography (ethyl acetate/hexane = 1/1) to afford **AP2** as a white solid (Yield: 15%). ^{1}H NMR (500 MHz, $CDCl_3$) 6.97 ~ 6.94 (m, 1H), 5.02 (s, 1H), 4.91 (s, 1H), 4.67 (dd, *J* = 11.9, 4.2 Hz, 1H), 4.60 (s, 1H), 4.45 (dd, *J* = 10.5, 6.1 Hz, 1H), 4.25 (dd, *J* = 10.5, 2.0 Hz, 1H), 4.12 (d, *J* = 11.9 Hz, 1H), 3.39 (t, *J* = 10.6 Hz, 1H), 2.55 ~ 2.52(m, 2H), 2.48 (t, *J* = 7.4 Hz, 2H), 2.44 (d, *J* = 13.0 Hz, 1H), 2.35 (d, *J* = 6.7 Hz, 1H), 2.27 (td, *J* = 6.9, 2.6 Hz, 2H), 1.99 (t, *J* = 2.6 Hz, 2H), 1.88 ~ 1.73 (m, 8H), 1.44 ~ 1.31 (m, 3H), 1.08 (s, 3H), 0.72 (s, 3H). ^{13}C NMR (125 MHz, $CDCl_3$) 172.01, 169.80, 148.55, 146.32, 128.11, 109.03, 82.83, 82.36, 74.27, 69.53, 66.22, 63.48, 55.84, 55.49, 42.64, 38.83, 37.67, 36.92, 33.12, 24.73, 24.21, 23.95, 23.55, 22.43, 17.71, 14.93. HRMS (ESI, $[M-H]^-$) calcd. for $C_{26}H_{35}O_6$: 443.2439; found 443.2435.

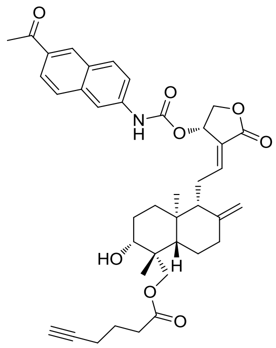

(**AP1NP**): 2-acetyl-6-amino-napthalene (30 mg, 0.162 mmol) was dissolved in dichloromethane (2 mL) and saturated sodium bicarbonate (2 mL) was added to the reaction mixture. The biphasic mixture was stirred at room temperature for 5 min and then the stirring was stopped so that a solution of triphosgene (29 mg, 0.097 mmol) in dichloromethane could be added into the dichloromethane layer. After the addition, stirring was resumed for another 10 ~ 30 min before the dichloromethane layer was removed *via* a syringe, dried over anhydrous $MgSO_4$ and concentrated to afford the corresponding crude isocyanate, which was used without further purification. The crude isocyanate and **AP1** (49 mg, 0.11 mmol) were dissolved in dichloromethane (4 mL) followed by the addition of triethylamine (34 µL, 0.243 mmol). The reaction mixture was stirred at room temperature for 2.5 h. The product precipitated out from the reaction mixture and was filtered and washed with MeOH/DCM (2/98 to give **AP1NP** as a white solid (Yield: 36%). ^{1}H NMR (500 MHz, $CDCl_3$) 8.44 (s, 1H), 8.13 ~ 8.01 (m, 2H), 7.96 (d, *J* = 8.8 Hz, 1H), 7.85 (d, *J* = 8.6 Hz, 1H), 7.52 (d, *J* = 8.5 Hz, 1H), 7.20 (s, 1H), 7.11 (t, *J* = 6.6 Hz, 1H), 6.06 (d, *J* = 5.5 Hz, 1H), 4.92 (s, 1H), 4.63 (dd, *J* = 11.3, 5.9 Hz, 1H), 4.55 (s, 1H), 4.45 (d, *J* = 11.4 Hz, 1H), 4.36 (d, *J* = 11.7 Hz, 1H), 4.13 (d, *J* = 11.8 Hz, 1H), 3.40 ~ 3.27 (m, 1H), 2.74 (s, 3H), 2.67 ~ 2.36 (m, 5H), 2.27 (m, 2H), 2.01 (m, 3H), 1.94 ~ 1.78 (m, 5H), 1.76 ~ 1.67 (m, 1H), 1.53 ~ 1.39 (m, 1H), 1.37 ~ 1.24 (m, 3H), 1.17 (s, 3H), 0.72 (s, 3H). ^{13}C NMR (125 MHz, DMSO-d_6) δ 197.50, 172.34, 169.03, 152.75, 149.36, 147.73, 138.89, 135.82, 132.79, 130.53, 130.16, 128.41, 127.45, 124.81, 124.20, 120.05, 113.51, 113.49, 107.81, 83.60, 76.51, 71.60, 68.14, 65.07, 59.74, 55.18, 53.91, 41.69, 37.52, 36.53, 32.58, 27.55, 26.59, 24.75, 24.58, 23.41, 22.73, 17.06, 13.85. HRMS (ESI $[M-H]^-$): calcd for $C_{39}H_{44}NO_8$: 654.3072; found 654.3092.

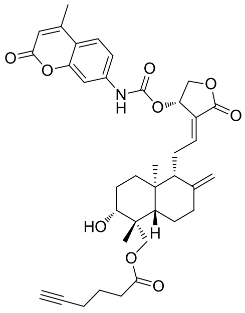

(**AP1CM**) was synthesized according to the similar procedure described in **AP1NP** by using 7-amino-4-methylcoumarin instead of 2-acetyl-6-amino-napthalene (white solid, Yield: 65%). ^{1}H NMR (500 MHz, DMSO-d_6) δ 10.39 (s, 1H), 7.71 (d, *J* = 8.7 Hz, 1H), 7.53 (s, 1H), 7.41 (dd, *J* = 8.6, 1.3 Hz, 1H), 6.91 (t, *J* = 6.7 Hz, 1H), 6.25 (s, 1H), 6.07 (d, *J* = 5.5 Hz, 1H), 4.82 (s, 1H), 4.70 (d, *J* = 4.7 Hz, 1H), 4.61 (dd, *J* = 11.0, 5.8 Hz, 1H), 4.51 (s, 1H), 4.44 (d, *J* = 11.1 Hz, 1H), 4.16 (d, *J* = 11.7 Hz, 1H), 4.05 (d, *J* = 11.7 Hz, 1H), 3.22 ~ 3.09 (m, 1H), 2.76 (t, *J* = 2.5 Hz, 1H), 2.66 ~ 2.55 (m, 1H), 2.48 ~ 2.41 (m, 1H), 2.39 (s, 3H), 2.32 (m, 2H), 2.16 (m, 2H), 2.00 ~ 1.88 (m, 2H), 1.79 (d, *J* = 13.3 Hz, 1H), 1.74 ~ 1.60 (m, 3H), 1.57 ~ 1.38 (m, 3H), 1.24 (m, 3H), 1.01 (s, 3H), 0.64 (s, 3H). ^{13}C NMR (125 MHz, DMSO-d_6) 172.33, 168.97, 159.95, 153.76, 153.13, 152.50, 149.52, 147.75, 142.28, 126.09, 124.70, 114.65, 114.38, 112.11, 107.75, 104.67, 83.60, 76.51, 71.61, 71.54, 68.27, 65.07, 55.19, 53.90, 41.68, 38.79, 37.51, 36.52, 32.58, 27.55, 24.74, 24.58, 23.42, 22.73, 17.96, 17.06, 13.84. HRMS (ESI [M-H]$^-$): calcd for $C_{37}H_{42}NO_9$: 644.2865; found: 644.2868.

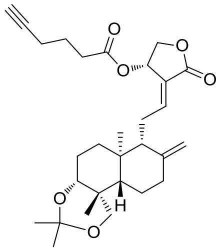

3,19-Isopropylidne 14-hexynoate andrographolide (**8**): To a solution of **2** (40 mg, 0.1 mmol) in dichloromethane (0.3 mL) were added 5-hexynoic acid (35 μL, 0.3 mmol) and coupling reagent *N,N'*-dicyclohexylcarbodiimide (69 mg, 0.33 mmol). The resulting mixture was stirred at room temperature for 4 days. The suspension was then filtered and the crude mixture was purified by flash chromatography (ethyl acetate/hexane = 1/4 to 1/2) to afford **8** as a white solid (Yield: 50%). ^{1}H NMR (500 MHz, $CDCl_3$) 7.02 (td, *J* = 6.8, 1.7 Hz, 1H), 5.94 (d, *J* = 6.1 Hz,

1H), 4.89 (s, 1H), 4.55 (dd, *J* = 11.2, 6.2 Hz, 1H), 4.52 (s, 1H), 4.23 (dd, *J* = 11.2, 1.9 Hz, 1H), 3.95 (d, *J* = 11.6 Hz, 1H), 3.49 (dd, *J* = 8.6, 4.0 Hz, 1H), 3.17 (d, *J* = 11.6 Hz, 1H), 2.52 (t, *J* = 7.4 Hz, 2H), 2.46 ~ 2.39 (m, 3H), 2.28 (td, *J* = 6.9, 2.6 Hz, 2H), 1.98 (t, *J* = 2.7 Hz, 2H), 1.86 (t, *J* = 7.1 Hz, 3H), 1.82 ~ 1.75 (m, 1H), 1.72 ~ 1.67 (m, 2H), 1.40 (s, 3H), 1.36 (s, 3H), 1.29 ~ 1.23 (m, 4H), 1.19 (s, 3H), 0.93 (s, 3H). ^{13}C NMR (125 MHz, $CDCl_3$) 172.60, 169.02, 150.66, 147.01, 123.75, 108.90, 99.13, 82.65, 76.10, 71.58, 69.58, 67.80, 63.89, 55.85, 52.13, 38.32, 37.88, 37.55, 34.48, 32.53, 26.99, 26.10, 25.45, 25.27, 24.89, 23.33, 23.10, 17.74, 16.14. HRMS (ESI, $[2M+Na]^+$) calcd. for $C_{58}H_{80}NaO_{12}$: 991.5542; found: 991.5537.

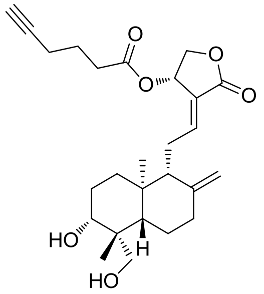

14-Hexynoate andrographolide (**AP3**): Compound **8** (20 mg, 0.04 mmol) was added to a mixture of acetic acid/water (7/3, 0.52 mL). The resulting mixture was stirred at room temperature for 30 min. Saturated sodium bicarbonate was then added to neutralize the acid. The aqueous layer was extracted with ethyl acetate (4×) and the combined organic layer was dried over anhydrous $MgSO_4$, filtered and the crude product obtained was purified by flash chromatography (ethyl acetate/hexane = 1/2 to 1/1) to obtain **AP3** as a white solid (82%). ^{1}H NMR (500 MHz, $CDCl_3$) 7.00 (t, *J* = 6.8 Hz, 1H), 5.93 (d, *J* = 5.6 Hz, 1H), 4.87 (s, 1H), 4.55 (dd, *J* = 10.9, 6.5 Hz, 1H), 4.48 (s, 1H), 4.23 (d, *J* = 11.3 Hz, 1H), 4.17 (d, *J* = 11.1 Hz, 1H), 3.49 ~ 3.46 (m, 1H), 3.31 (d, *J* = 10.8 Hz, 1H), 2.58 (s, 2H), 2.51 (t, *J* = 7.4 Hz, 2H), 2.47 ~ 2.33(m, 3H), 2.27 (td, *J* = 6.7, 2.5 Hz, 2H), 1.98 ~ 1.93 (m, 2H), 1.88 ~ 1.79 (m, 6H), 1.72 (dt, *J* = 12.6, 3.1 Hz, 1H), 1.33 ~ 1.26 (m, 2H), 1.24 (s, 3H), 1.20 ~ 1.17 (m, 1H), 0.66 (s, 3H). ^{13}C NMR (125 MHz, $CDCl_3$) 172.64, 169.03, 150.55, 146.63, 123.77, 108.83, 82.63, 80.34, 71.58, 69.59, 67.74, 64.06, 55.76, 55.13, 42.83, 38.78, 37.65, 36.96, 32.51, 28.12, 25.29, 23.64, 23.31, 22.68, 17.73, 15.10. HRMS (ESI, $[M-H]^-$) calcd. for $C_{26}H_{35}O_6$: 443.2439; found: 443.2435.

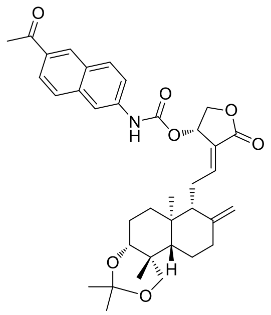

Compound (**9**): 2-acetyl-6-amino-napthalene (30 mg, 0.162 mmol) was dissolved in dichloromethane (2 mL) and saturated sodium bicarbonate (2 mL) was added to the reaction mixture. The biphasic mixture was stirred at room temperature for 5 min and then the stirring was stopped so that a solution of triphosgene (29 mg, 0.097 mmol) in dichloromethane could be added into the dichloromethane layer. After the addition, stirring was resumed for another 10 ~ 30 minutes before the dichloromethane layer was removed *via* a syringe, dried over anhydrous $MgSO_4$ sulphate and concentrated to afford the corresponding crude isocyanate, which was used without further purification. The crude isocyanate and **2** (43 mg, 0.11 mmol) were dissolved in dichloromethane (4 mL) followed by the addition of triethylamine (34 μL, 0.243 mmol). The reaction mixture was stirred at room temperature for 2.5 h. The product precipitated out from the reaction mixture and was filtered and washed with MeOH/DCM (2/98) to afford **9** as a white solid (Yield: 58%). ^{1}H NMR (500 MHz, DMSO-d_6) 10.33 (s, 1H), 8.58 (s, 1H), 8.15 (s, 1H), 8.08 (d, *J* = 8.9 Hz, 1H), 7.94 (d, *J* = 8.5 Hz, 1H), 7.88 (d, *J* = 8.6 Hz, 1H), 7.65 (d, *J* = 8.6 Hz, 1H), 6.92 (t, *J* = 6.6 Hz, 1H), 6.09 (d, *J* = 5.3 Hz, 1H), 4.85 (s, 1H), 4.65 (dd, *J* = 11.0, 5.8 Hz, 1H), 4.57 (s, 1H), 4.46 (d, *J* = 10.9 Hz, 1H), 3.84 (d, *J* = 11.6 Hz, 1H), 3.38 (dd, *J* = 8.9, 3.1 Hz, 1H), 3.08 (d, *J* = 11.6 Hz, 1H), 2.68 (s, 3H), 2.59 (d, *J* = 6.9 Hz, 1H), 2.33 (d, *J* = 12.6 Hz, 1H), 2.03 ~ 1.85 (m, 3H), 1.67 (t, *J* = 10.1 Hz, 3H), 1.36 ~ 1.16 (m, 9H), 1.11 (s, 3H), 0.83 (s, 3H). ^{13}C NMR (125 MHz, DMSO-d_6) 197.46, 168.99, 152.70, 149.21, 147.68, 138.88, 135.79, 132.77, 130.50, 130.13, 128.39, 127.42, 124.82, 124.17, 120.01, 113.45, 108.24, 98.12, 75.73, 71.59, 68.10, 62.72, 54.85, 51.41, 38.03, 37.12, 36.92, 33.94, 27.41, 26.57, 25.73, 25.16, 24.86, 24.81, 22.61, 15.57. HRMS (ESI) [M-H]$^-$: calcd for $C_{36}H_{42}NO_7$, 600.2967; found 600.2965.

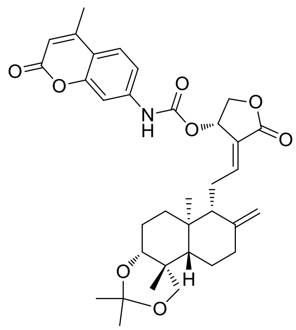

Compound (**10**) was synthesized according to the similar procedure described in **9** by using 7-amino-4-methylcoumarin instead of 2-acetyl-6-amino-napthalene (white solid, Yield: 72%). ^{1}H NMR (500 MHz, DMSO-d_6) 10.42 (s, 1H), 7.73 (d, *J* = 8.5 Hz, 1H), 7.55 (s, 1H), 7.42 (d, *J* = 8.7 Hz, 1H), 6.91 (t, *J* = 6.9 Hz, 1H), 6.27 (s, 1H), 6.09 (d, *J* = 5.2 Hz, 1H), 4.85 (s, 1H), 4.62 (dd, *J* = 11.1, 5.7 Hz, 1H), 4.55 (s, 1H), 4.45 (d, *J* = 11.1 Hz, 1H), 3.85 (d, *J* = 11.6 Hz, 1H), 3.09 (d, *J* = 11.6 Hz, 1H), 2.40 (s, 3H), 2.34 (d, *J* = 12.8 Hz, 1H), 2.13 ~ 1.83 (m, 3H), 1.68 (m, 3H), 1.28 (m, 9H), 1.12 (s, 3H), 0.83 (s, 3H). HRMS (ESI $[M+Na]^+$): calcd for $C_{34}H_{41}NNaO_8$: 614.2724; found 614.2738.

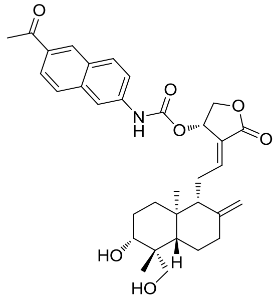

(**APNP**): Compound **9** (48 mg, 0.08 mmol) was dissolved in a mixture of THF/H_2O (7/3, 3.2 mL) and stirred at 0 ℃. trifluoroacetic acid (230 μL, 3 mmol) was then added dropwise to the reaction mixture. After the addition of trifluoroacetic acid, the reaction mixture was allowed to stir at room temperature for 1 h. Thereafter, the mixture was cooled to 0 ℃, neutralized with saturated $NaHCO_3$ and extracted with ethyl acetate. The combined organic layer was dried over anhydrous $MgSO_4$, concentrated and the crude product obtained was purified by flash chromatography (DCM to DCM/MeOH = 98/2) to afford **APNP** as a white solid (Yield: 88%). ^{1}H NMR (500 MHz, $CDCl_3$) 8.45 (s, 1H), 8.06 (d, *J* = 8.6 Hz, 2H), 7.97 (d, *J* = 8.8 Hz, 1H), 7.86 (d, *J* = 8.6 Hz, 1H), 7.51 (d, *J* = 8.9 Hz, 1H), 7.16 (s, 1H), 7.11 (t, *J* = 6.9 Hz, 1H), 6.06 (d, *J* = 5.6 Hz, 1H), 4.90 (s, 1H), 4.63 (dd, *J* = 11.5, 5.8 Hz, 1H), 4.54 (s, 1H), 4.45 (d, *J* = 11.4 Hz, 1H),

4.18 (d, J = 11.3 Hz, 1H), 3.57 ~ 3.43 (m, 1H), 3.33 (d, J = 10.8 Hz, 1H), 2.54 (m, 4H), 2.05 ~ 1.96 (m, 1H), 1.93 ~ 1.75 (m, 6H), 1.36 ~ 1.19 (m, 8H), 0.68 (s, 3H). ^{13}C NMR (125 MHz, DMSO-d_6) 197.99, 169.51, 153.23, 149.87, 148.28, 139.38, 136.30, 133.28, 131.01, 130.65, 128.90, 127.94, 125.27, 124.70, 120.53, 108.26, 78.88, 72.10, 68.60, 63.05, 55.62, 54.82, 42.72, 39.12, 37.84, 36.94, 28.35, 27.09, 25.17, 24.38, 23.51, 15.05. HRMS (ESI [M-H]$^-$): calcd for $C_{33}H_{38}NO_7$, 560.2654; found 560.2665.

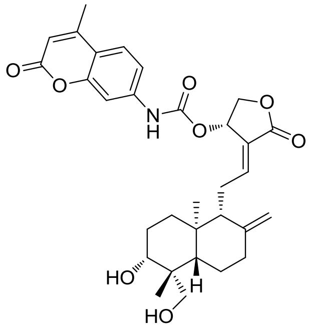

(**APCM**): was synthesized according to the similar procedure described in **APNP** by using **10** instead of **9** (white solid, Yield: 91%). ^{1}H NMR (500 MHz, DMSO-d_6) 10.40 (s, 1H), 7.81 ~ 7.68 (m, 1H), 7.54 (s, 1H), 7.41 (dd, J = 8.7, 1.6 Hz, 1H), 6.90 (t, J = 6.6 Hz, 1H), 6.26 (s, 1H), 6.07 (d, J = 5.5 Hz, 1H), 4.80 (s, 1H), 4.61 (dd, J = 11.1, 5.8 Hz, 1H), 4.49 (s, 1H), 4.44 (d, J = 10.6 Hz, 1H), 3.80 (d, J = 11.0 Hz, 1H), 3.27 ~ 3.15 (m, 2H), 2.46 ~ 2.37 (m, 5H), 2.35 ~ 2.24 (m, 1H), 2.01 ~ 1.85 (m, 2H), 1.79 ~ 1.52 (m, 4H), 1.41 ~ 1.14 (m, 4H), 1.06 (s, 3H), 0.61 (s, 3H).^{13}C NMR (125 MHz, DMSO-d_6) 168.99, 159.97, 153.77, 153.13, 152.51, 149.58, 147.81, 142.30, 126.07, 124.69, 114.64, 114.39, 112.11, 107.75, 104.66, 78.41, 71.56, 68.26, 62.59, 55.16, 54.33, 42.23, 38.63, 37.35, 36.44, 27.87, 24.72, 23.90, 23.02, 17.97, 14.55. HRMS (ESI [M+Na]$^+$): calcd for $C_{31}H_{37}NNaO_8$: 574.2411; found 574.2416.

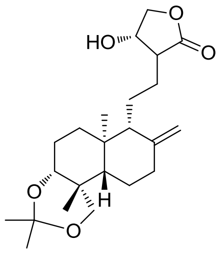

Compound (**11**): To a solution of compound **2** (100 mg, 0.26 mmol) in anhydrous methanol (1 mL) was added sodium borohydride ($NaBH_4$, 12 mg, 0.31 mmol). The resulting mixture was stirred at room temperature for 1 h. The crude mixture was directly purified by flash

chromatography (ethyl acetate/hexane = 1/2 to 2/3) to afford **11** as a white solid (Yield: 50%). ^{1}H NMR (500 MHz, $CDCl_3$) 4.84 (s, 1H), 4.63 (s, 1H), 4.58 ~ 4.56 (m, 1H), 4.30 ~ 4.24 (m, 2H), 3.95 (d, *J* = 11.6 Hz, 1H), 3.48 (dd, *J* = 8.6, 3.6 Hz, 1H), 3.15 (d, *J* = 11.6 Hz, 1H), 2.47 ~ 2.44 (m, 1H), 2.40 ~ 2.36 (m, 2H), 2.03 ~ 1.99 (m, 2H), 1.85 ~ 1.69 (m, 4H), 1.65 ~ 1.55 (m, 4H), 1.40 (s, 3H), 1.35 (s, 3H), 1.31 ~ 1.21 (m, 3H), 1.18 (s, 3H), 0.89 (s, 3H). ^{13}C NMR (125 MHz, $CDCl_3$) 177.75, 147.57, 107.29, 99.07, 76.39, 74.44, 69.12, 63.93, 56.65, 52.30, 45.65, 38.53, 38.06, 37.88, 34.44, 27.10, 26.08, 25.29, 25.00, 23.45, 22.91, 22.23, 16.28. HRMS (ESI, $[M-H]^-$) calcd. for $C_{23}H_{35}O_5$: 391.2490; found 391.2496.

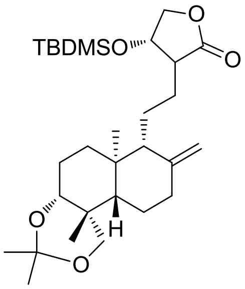

Compound (**12**): To a solution of **11** (80 mg, 0.20 mmol) in anhydrous DMF (0.3 mL) were added imidazole (68 mg, 1.00 mmol) and *t*-butyldimethylsilyl chloride (TBDMSCl, 77 mg, 0.50 mmol). The resulting mixture was heated to 80 ^{o}C and stirred for 30 min. The reaction mixture was then diluted with diethyl ether and washed with water (2 ×) and brine (2 ×). The organic layer was dried over anhydrous $MgSO_4$, filtered and the crude mixture was purified by flash chromatography (ethyl acetate/hexane = 1/6 to 1/4) to afford **12** as a white solid (Yield: 50%). ^{1}H NMR (500 MHz, $CDCl_3$) 4.84 (s, 1H), 4.55 (s, 1H), 4.51 ~ 4.49 (m, 1H), 4.22 (dd, *J* = 9.9, 3.6 Hz, 1H), 4.11 (d, *J* = 9.9 Hz, 1H), 3.95 (d, *J* = 11.6 Hz, 1H), 3.47 (dd, *J* = 8.6, 3.6 Hz, 1H), 3.15 (d, *J* = 11.6 Hz, 1H), 2.38 (d, *J* = 10.4 Hz, 2H), 2.01 ~ 1.90 (m, 2H), 1.85 ~ 1.67 (m, 4H), 1.62 ~ 1.52 (m, 4H), 1.39 (s, 3H), 1.35 (s, 3H), 1.27 ~ 1.23 (m, 3H), 1.18 (s, 3H), 0.88 (s, 3H), 0.87 (s, 9H), 0.07 (s, 6H).^{13}C NMR (125 MHz, $CDCl_3$) 177.81, 147.30, 107.57, 98.97, 76.38, 74.02, 69.92, 63.88, 56.35, 52.29, 45.84, 38.47, 37.95, 37.83, 34.55, 27.15, 26.04, 25.57, 25.20, 24.99, 23.39, 22.77, 21.89, 17.90, 16.26, -4.56, -4.86. HRMS (ESI, $[2M+Na]^+$) calcd. for $C_{58}H_{100}NaO_{10}Si_2$: 1035.6747; found 1035.6768.

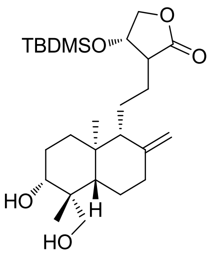

Compound (**13**): To a solution of **12** (50 mg, 0.16 mmol) in THF/H_2O (4/1, 0.50 mL) at 0 ^oC was added trifluoroacetic acid (18 μL, 0.23 mmol). The resulting mixture was then warmed up to room temperature and stirred for 1 h. Thereafter, saturated sodium bicarbonate was added to neutralize the acid. The aqueous layer was then extracted with ethyl acetate (4×) and the combined organic layer was dried over anhydrous $MgSO_4$, filtered and concentrated to yield **13** as a white solid (Yield: 99%). ^1H NMR (500 MHz, $CDCl_3$) 4.82 (s, 1H), 4.51 (s, 1H), 4.49 ~ 4.47 (m, 1H), 4.21 (dd, *J* = 9.9, 3.6 Hz, 1H), 4.15 (d, *J* = 10.8 Hz, 1H), 4.10 (d, *J* = 9.9 Hz, 1H), 3.45 - 3.42 (m, 1H), 3.28 (d, *J* = 10.8 Hz, 1H), 2.36 (t, *J* = 10.9 Hz, 2H), 1.95 - 1.90 (m, 1H), 1.80 - 1.77 (m, 4H), 1.64 ~ 1.42 (m, 4H), 1.28 ~ 1.23 (m, 2H), 1.21 (s, 3H), 1.19 ~ 1.16 (m, 2H), 0.86 (s, 9H), 0.61 (s, 3H), 0.07 (s, 3H), 0.06 (s, 3H). ^{13}C NMR (125 MHz, $CDCl_3$) 177.97, 146.98, 107.34, 80.44, 74.07, 69.97, 64.12, 56.47, 55.23, 45.99, 42.71, 38.96, 38.07, 36.95, 28.14, 25.57, 23.92, 22.81, 22.70, 21.77, 17.90, 15.19, -4.59, -4.86. HRMS (ESI, $[2M+Na]^+$) calcd. for $C_{52}H_{92}NaO_{10}Si_2$: 955.6121; found 955.6139.

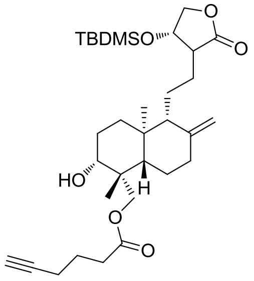

Compound (**14**): To a solution of **13** (50 mg, 0.11 mmol) in dichloromethane (1.2 mL) were added 5-hexynoic acid (15 μL, 0.13 mmol), coupling reagent *N,N'*-dicyclohexylcarbodiimide (29 mg, 1.32 mmol) and 4-dimethylaminopyridine (2 mg, 0.01 mmol). The resulting mixture was stirred at room temperature for 10 h. The suspension was then filtered and the crude mixture was purified by flash chromatography (ethyl acetate/hexane = 1/4 to 1/2) to afford **14** as a white solid (Yield: 50%). ^1H NMR (500 MHz, DMSO-d_6) 4.82 (s, 1H), 4.68 (d, *J* = 4.5 Hz, 1H), 4.58 ~ 4.56 (m, 1H), 4.53 (s, 1H), 4.27 (dd, *J* = 10.0, 2.9 Hz, 1H), 4.17 (d, *J* = 11.6 Hz, 1H), 4.07 (d, *J* = 11.7

Hz, 1H), 4.02 (d, J = 10.0 Hz, 1H), 3.19 ~ 3.15 (m, 1H), 2.78 (t, J = 2.3 Hz, 1H), 2.68 ~ 2.64 (m, 1H), 2.35 (t, J = 7.3 Hz, 2H), 2.18 (td, J = 7.1, 2.5 Hz, 2H), 1.91 - 1.79 (m, 2H), 1.72 ~ 1.63 (m, 5H), 1.57 ~ 1.50 (m, 3H), 1.45 ~ 1.33 (m, 2H), 1.23 (t, J = 14.3 Hz, 3H), 1.03 (s, 3H), 0.84 (s, 9H), 0.63 (s, 3H), 0.09 (s, 3H), 0.07 (s, 3H). ^{13}C NMR (125 MHz, DMSO-d_6) 177.93, 172.32, 147.62, 106.67, 83.59, 76.55, 73.98, 71.60, 69.86, 65.13, 55.37, 54.10, 44.90, 41.71, 38.06, 36.72, 33.32, 32.62, 27.60, 25.53, 25.05, 23.45, 22.74, 22.00, 20.76, 17.60, 17.09, 14.13, -4.66, -5.05. HRMS (ESI, M + Na) calcd. for C32H52NaO6Si, 583.3431; found 583.3439.

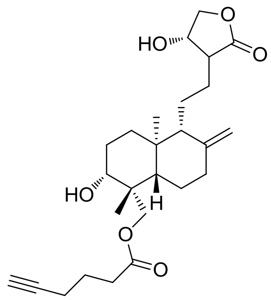

(**NC**): To a solution of **14** (30 mg, 0.05 mmol) in methanol (0.3 mL) was added cerium (IV) ammonium nitrate (35 mg, 0.06 mmol). The resulting mixture was stirred at room temperature for 20 h. The crude reaction mixture was subsequently purified by flash chromatography (ethyl acetate/hexane = 1/1 to 1/2) to afford **NC** as a white solid (Yield: 17%). ^{1}H NMR (500 MHz, $CDCl_3$) 4.84 (s, 1H), 4.62 (s, 1H), 4.56 ~ 4.55 (m, 1H), 4.34 (d, J = 11.7 Hz, 1H), 4.30 ~ 4.24 (m, 2H), 4.09 (d, J = 11.7 Hz, 1H), 3.31 (dd, J = 11.8, 3.8 Hz, 1H), 2.56 (s, 1H), 2.45 - 2.41 (m, 3H), 2.39 (s, 1H), 2.24 (td, J = 6.9, 2.5 Hz, 2H), 1.97 (t, J = 2.3 Hz, 1H), 1.92 (dd, J = 12.8, 4.6 Hz, 1H), 1.85 ~ 1.78 (m, 6H), 1.68 ~ 1.49 (m, 6H), 1.46 ~ 1.37 (m, 1H), 1.25 ~ 1.20 (m, 2H), 1.13 (s, 3H), 0.66 (s, 3H).^{13}C NMR (125 MHz, $CDCl_3$) 178.00, 173.16, 146.84, 107.32, 83.08, 78.81, 74.59, 69.24, 68.93, 64.97, 56.50, 55.26, 45.59, 42.39, 39.11, 38.16, 37.07, 32.96, 27.71, 24.42, 23.43, 22.67, 22.39, 21.84, 17.71, 14.78. HRMS (ESI, $[M+Na]^+$) calcd. for $C_{26}H_{38}NaO_6$: 469.2561; found 469.2575.

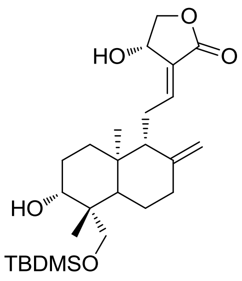

19-*t*-Butyldimethylsilyl andrographolide (**15**): To a stirred solution of **WT** (1.0 g, 2.85 mmol) in pyridine (15 mL) was added *t*-butyldimethylsilyl chloride (TBDMSCl, 2.58 g, 17.1 mmol) at room temperature. After the stirring was continued at room temperature for 1 h, the reaction mixture was diluted with ethyl acetate (20 mL) and poured into 1 M $CuSO_4$ (150 mL) solution and extracted with ethyl acetate (3×). The combined organic layer was washed with brine (20 mL), dried over anhydrous $MgSO_4$ and concentrated. The residue was purified by column chromatography (ethyl acetate/hexane = 1/1) to give **15** as a white solid (Yield: 90%). ^{1}H NMR (500 MHz, $CDCl_3$) 6.96 (t, *J* = 6.5 Hz, 1H), 5.02 (t, *J* = 6.1 Hz, 1H), 4.88 (s, 1H), 4.59 (s, 1H), 4.49 ~ 4.41 (m, 2H), 4.25 (dd, *J* = 10.4, 1.9 Hz, 1H), 4.19 (d, *J* = 10.0 Hz, 1H), 3.38 (d, *J* = 10.1 Hz, 1H), 3.31 (m, 1H), 2.67 ~ 2.47 (m, 3H), 2.41 (dd, *J* = 13.2, 3.3 Hz, 1H), 2.01 ~ 1.69 (m, 6H), 1.24 (d, *J* = 13.6 Hz, 6H), 0.89 (s, 9H), 0.70 (s, 3H), 0.06 (d, *J* = 3.6 Hz, 6H). ^{13}C NMR (125 MHz, $CDCl_3$) 170.45, 149.26, 147.14, 128.59, 109.25, 80.68, 74.84, 66.70, 65.74, 56.63, 55.80, 43.03, 39.51, 38.31, 37.71, 29.12, 26.30, 25.23, 24.39, 23.70, 18.59, 16.01, -5.20, -5.28. HRMS (ESI, $[M-H]^-$) calcd. for $C_{26}H_{43}O_5Si$: 463.2885; found 463.2885.

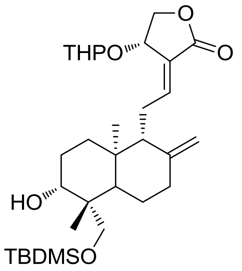

19-*t*-Butyldimethylsilyl-14-tetrahydropyranyl andrographolide (**16**): To a stirred solution of **15** (0.8 g, 1.72 mmol) and 3,4-dihydro-2H-pyran (235 μL, 2.58 mmol) in dichloromethane (30 ml) at room temperature was added *p*-toluenesulfonic acid (0.144 g, 0.76 mmol). The mixture was stirred at room temperature for 40 min. The reaction mixture was then directly purified by flash chromatography (ethyl acetate/hexane = 3/7) to give **16** as a white solid (Yield: 78%). The racemic compound was used for the next step without further characterization.

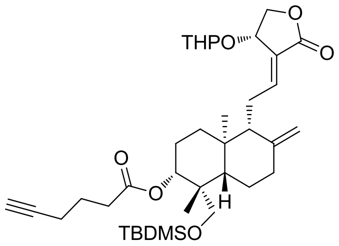

19-*t*-Butyldimethylsilyl-14-tetrahydropyranyl-3-hexynoate andrographolide (**17**): To a solution of **16** (700 mg, 1.28 mmol) in dichloromethane (15 mL) were added 5-hexynoic acid (722 μL, 6.38 mmol), coupling reagent *N,N'*-dicyclohexylcarbodiimide (1.45 g, 7.02 mmol) and 4-dimethylaminopyridine (78 mg, 0.64 mmol). The resulting mixture was stirred at room temperature for 10 h. The suspension was then filtered and the crude mixture was purified by flash chromatography (ethyl acetate/hexane = 1/10 to 1/18) to afford **17** as a white solid (Yield: 94%). The racemic compound was used for the next step without further characterization.

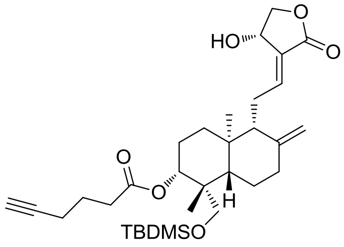

19-*t*-Butyldimethylsilyl-3-hexynoate andrographolide (**18**): To a solution of **17** (85 mg, 0.13 mmol) in dichloromethane (1.2 mL) containing 5% (v/v) of 1, 2-ethanedithiol at -40 ℃ was added Lewis acid BF_3•Et_2O (8 μL, 0.07 mmol) slowly and the resulting solution is allowed to stir for 30 min. Thereafter, the reaction mixture was poured into saturated aqueous sodium bicarbonate solution and extracted with dichloromethane. The combine organic extract was dried over anhydrous $MgSO_4$ and concentrated. The crude product was purified by flash column chromatography (ethyl acetate/hexane = 3/7 to 1/3) to provide **18** as a white solid (Yield: 73%). ^{1}H NMR (500 MHz, $CDCl_3$) 6.99 (t, *J* = 6.4 Hz, 1H), 5.03 (s, 1H), 4.90 (s, 1H), 4.58 (dd, *J* = 10.2, 6.0 Hz, 2H), 4.45 (dd, *J* = 10.4, 6.1 Hz, 1H), 4.26 (dd, *J* = 10.5, 1.8 Hz, 1H), 3.84 (d, *J* = 10.5 Hz, 1H), 3.60 (d, *J* = 10.5 Hz, 1H), 2.62 ~ 2.48 (m, 2H), 2.48 ~ 2.36 (m, 3H), 2.26 (m, 3H), 1.97 (t, *J* = 2.5 Hz, 1H), 1.94 ~ 1.78 (m, 6H), 1.78 ~ 1.66 (m, 3H), 1.38 ~ 1.21 (m, 2H), 0.94 (s, 3H), 0.88 (s, 9H), 0.84 (s, 3H), 0.03 (s, 6H). ^{13}C NMR (125 MHz, $CDCl_3$) δ 173.03, 170.16, 149.27, 147.43, 128.27, 108.65, 83.45, 80.50, 74.55, 69.43, 66.51, 64.04, 56.51, 55.83, 42.78,

39.41, 38.58, 37.66, 33.57, 26.17, 25.70, 25.03, 24.66, 24.02, 23.54, 18.52, 18.15, 14.66, -5.33, -5.42. HRMS (ESI, $[M-H]^-$) calcd. for $C_{32}H_{49}O_6Si$: 557.3304; found 557.3302.

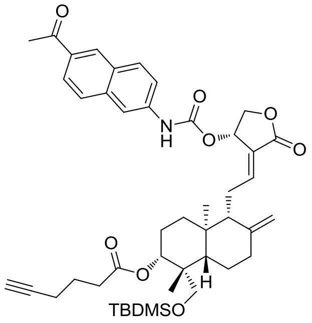

Compound (**19**): 2-acetyl-6-amino-napthalene (60 mg, 0.324 mmol) was dissolved in dichloromethane (4 mL) and saturated sodium bicarbonate (4 mL) was added to the solution. The biphasic mixture was stirred at room temperature for 5 min and momenterily stopped the stirring procedure to add the triphosgene (58 mg, 0.194 mmol) solution in dichloromethane into the dichloromethane layer. Thereafter stirriring was continued for another 10 ~ 30 min. The dichloromethane layer was removed and dried over anhydrous $MgSO_4$. The filtrate was evaporated to afford the corresponding crude isocyanate. The crude isocyanate and **17** (85 mg, 0.15 mmol) were dissolved in dichloromethane (4 mL) and stirred at -20 ℃ for 4 ~ 5 min. After which, diluted TEA (67.5 μL, 0.486 mmol) in dichloromethane (500 μL) was added very slowly to the reaction mixture which was then stirred at -20 ℃ for 2.5 h. The crude reaction mixture was directly purified by flash column chromatography (ethyl acetate/hexane = 1/4) to afford **19** as a white solid (Yield: 66%). ^{1}H NMR (500 MHz, $CDCl_3$) 8.41 (s, 1H), 8.14 ~ 7.98 (m, 2H), 7.93 (d, J = 8.8 Hz, 1H), 7.83 (d, J = 8.7 Hz, 1H), 7.50 (dd, J = 8.8, 1.6 Hz, 1H), 7.10 (dd, J = 6.7, 5.9 Hz, 1H), 6.04 (d, J = 5.6 Hz, 1H), 4.88 (s, 1H), 4.60 (m, 2H), 4.51 (s, 1H), 4.42 (dd, J = 11.4, 0.9 Hz, 1H), 3.82 (d, J = 10.6 Hz, 1H), 3.57 (d, J = 10.5 Hz, 1H), 2.72 (s, 3H), 2.63 ~ 2.54 (m, 1H), 2.53 ~ 2.34 (m, 4H), 2.26 (m, 2H), 1.96 (t, J = 2.6 Hz, 1H), 1.94 ~ 1.77 (m, 6H), 1.76 ~ 1.63 (m, 3H), 1.42 ~ 1.20 (m, 3H), 0.93 (s, 3H), 0.86 (s, 9H), 0.80 (s, 3H), -0.00 (d, J = 3.9 Hz, 6H). ^{13}C NMR (125 MHz, $CDCl_3$) 197.85, 172.68, 169.31, 152.46, 151.24, 147.37, 137.17, 136.34, 133.74, 130.87, 129.90, 129.33, 127.92, 124.92, 123.90, 119.72, 114.7, 108.30, 83.18, 80.10, 71.85, 69.16, 68.74, 63.80, 56.07, 55.54, 42.48, 39.15, 38.28, 37.38, 33.28, 26.63, 25.88, 25.45, 25.38, 24.40, 23.75, 23.28, 18.23, 17.87, 14.37, -5.62, -5.72. HRMS (ESI, $[M-H]^-$) calcd. for $C_{45}H_{58}NO_8Si$: 768.3937; found 768.3911.

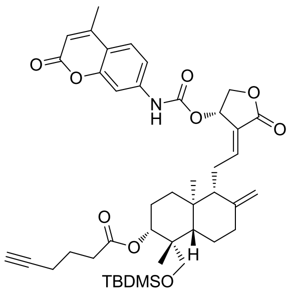

Compound (**20**) was synthesized according to the similar procedure described in **19** by using 7-amino-4-methylcoumarin instead of 2-acetyl-6-amino-napthalene (white solid, Yield: 60%). ^{1}H NMR (500 MHz, $CDCl_3$) 7.81 (s, 1H), 7.61 ~ 7.51 (m, 2H), 7.42 (d, *J* = 8.5 Hz, 1H), 7.08 (t, *J* = 6.5 Hz, 1H), 6.20 (s, 1H), 6.01 (d, *J* = 5.3 Hz, 1H), 4.85 (s, 1H), 4.58 (m, 2H), 4.48 (s, 1H), 4.40 (d, *J* = 11.3 Hz, 1H), 3.83 (d, *J* = 10.5 Hz, 1H), 3.58 (d, *J* = 10.5 Hz, 1H), 2.58 (m, 1H), 2.51 ~ 2.32 (m, 6H), 2.25 (td, *J* = 6.9, 2.6 Hz, 2H), 1.96 (t, *J* = 2.5 Hz, 1H), 1.93 ~ 1.77 (m, 6H), 1.75 ~ 1.56 (m, 3H), 1.38 ~ 1.20 (m, 3H), 0.93 (s, 3H), 0.86 (s, 9H), 0.80 (s, 3H), 0.00 (d, *J* = 3.0 Hz, 6H). ^{13}C NMR (125 MHz, $CDCl_3$) 172.96, 169.79, 161.38, 154.67, 152.60, 152.50, 151.77, 147.60, 141.38, 125.69, 124.17, 116.09, 114.87, 113.58, 108.55, 106.52, 83.45, 80.39, 72.16, 69.43, 68.92, 64.05, 56.35, 55.80, 42.76, 39.45, 38.54, 37.65, 33.55, 26.15, 25.77, 25.65, 24.67, 24.01, 23.52, 18.85, 18.50, 18.14, 14.63, -5.35, -5.44. HRMS (ESI, $[M-H]^-$) calcd. for $C_{43}H_{56}NO_9Si$:, 758.3730; found 758.3726.

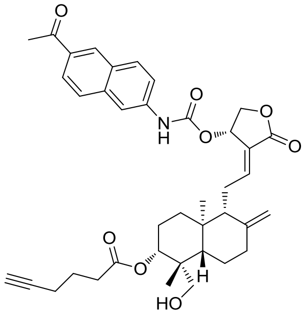

(**AP2NP**): To a solution of **19** (20 mg, 0.03 mmol) in methanol/dichloromethane (3 mL, 2/1) was added cerium (IV) ammonium nitrate (79 mg, 0.135 mmol). The resulting mixture was stirred at room temperature for 20 h. The crude reaction mixture was subsequently purified by either flash chromatography or preparative TLC (ethyl acetate/hexane = 1/1) to afford **AP2NP** as brown solid (Yield: 48%). ^{1}H NMR (500 MHz, $CDCl_3$) 8.42 (s, 1H), 8.04 (dd, *J* = 8.5, 1.5 Hz, 2H), 7.94 (d, *J* = 8.8 Hz, 1H), 7.84 (d, *J* = 8.7 Hz, 1H), 7.48 (d, *J* = 8.3 Hz, 1H), 7.08 (t, *J* = 7.3 Hz, 1H), 6.99 (s, 1H), 6.03 (d, *J* = 5.6 Hz, 1H), 4.90 (s, 1H), 4.68 (dd, *J* = 11.6, 4.2 Hz, 1H), 4.60 (dd, *J* =

11.4, 5.8 Hz, 1H), 4.53 (s, 1H), 4.43 (dd, *J* = 11.4, 1.0 Hz, 1H), 4.11 (m, 2H), 3.36 (t, *J* = 11.2 Hz, 1H), 2.72 (s, 3H), 2.57 (m, 1H), 2.52 ~ 2.38 (m, 3H), 2.27 (m, 2H), 2.02 ~ 1.92 (m, 2H), 1.91 ~ 1.70 (m, 6H), 1.36 (m, 3H), 1.26 (m, 3H), 1.07 (s, 3H), 0.69 (s, 3H). ^{13}C NMR (125 MHz, $CDCl_3$) 198.10, 172.24, 169.50, 152.70, 151.00, 146.79, 137.38, 136.59, 134.04, 131.17, 130.16, 129.61, 128.17, 125.22, 124.39, 119.95, 115.01, 109.19, 83.11, 82.53, 72.09, 69.82, 68.90, 63.73, 55.93, 55.73, 42.90, 39.13, 37.90, 37.15, 33.38, 26.89, 25.66, 24.49, 24.16, 23.82, 22.71, 17.98, 15.17. HRMS (ESI, $[M+Na]^+$) calcd. for $C_{39}H_{45}NNaO_8$: 678.3037; found 678.3047.

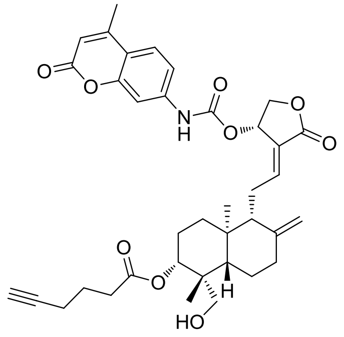

(**AP2CM**) was synthesized according to the similar procedure described in **AP2NP** by using **20** instead of **19** (white solid, Yield: 34%). ^{1}H NMR (500 MHz, $CDCl_3$) 7.69 ~ 7.51 (m, 3H), 7.47 ~ 7.35 (m, 1H), 7.09 (t, *J* = 6.6 Hz, 1H), 6.23 (s, 1H), 6.04 (d, *J* = 5.4 Hz, 1H), 4.90 (s, 1H), 4.70 (dd, *J* = 11.8, 4.1 Hz, 1H), 4.60 (dd, *J* = 11.3, 5.7 Hz, 1H), 4.52 (s, 1H), 4.43 (d, *J* = 11.3 Hz, 1H), 4.14 (d, *J* = 11.8 Hz, 1H), 3.47 ~ 3.34 (m, 1H), 2.59 (m, 1H), 2.54 ~ 2.36 (m, 6H), 2.29 (m, 2H), 2.06 ~ 1.94 (m, 2H), 1.92 ~ 1.75 (m, 6H), 1.46 ~ 1.20 (m, 6H), 1.09 (s, 3H), 0.71 (s, 3H). ^{13}C NMR (125 MHz, $CDCl_3$) 172.26, 169.57, 161.28, 154.69, 152.52, 152.39, 151.21, 146.78, 141.14, 125.74, 124.36, 116.21, 114.82, 113.71, 109.18, 106.55, 83.13, 82.53, 72.04, 69.82, 68.93, 63.74, 55.93, 55.73, 42.90, 39.16, 37.90, 37.15, 33.38, 26.16, 25.70, 24.50, 24.16, 23.82, 22.71, 18.86, 17.99, 15.18. HRMS (ESI, $[M-H]^-$) calcd. for $C_{37}H_{42}NO_8$: 644.2865; found 644.2879.

3. Fluorescence Measurements

Unless otherwise indicated, fluorescence measurements were performed as previously described[1-3]. Fluorescence emission data were recorded at 500 nm (λ_{ex} = 350 nm) for **APNP**/**AP1NP**/**AP2NP** and 450 nm (λ_{ex} = 350 nm) for **APCM**/**AP1CM**/**AP2CM** (**Supplementary Fig. S1** and **Table 1** & **Supplementary Table S1**). *In vitro* GSH assays were carried out in PBS buffer (*p*H 7.5) supplemented with 0.02% Triton X-100 at room temperature (**Fig. S1c-e**). The assays were performed in a final volume of 25 μL with 2.0 μM of the probe. For NEM inhibition assays, GSH was pretreated with NEM for 20 min prior to incubation with the probe, followed by fluorescence measurements. GSH assays in mammalian cell lysates were carried out with human hepatocellular carcinoma (HepG2) cells. Cells were cultured in DMEM medium supplemented with 10% FBS in a humidified 5% CO_2 incubator at 37 ℃, until they reached 80 ~ 90% confluence. Subsequently, the medium was carefully aspirated, and cells were washed with PBS (10.0 mL), then harvested by scraping in fresh PBS (20.0 mL). Cell pellets were isolated by centrifugation (1200 rpm, 5 min, 4 ℃), resuspended in 500 μL PBS supplemented with 0.02% Triton X-100 and lysed by sonication on ice. The soluble fraction was collected by centrifugation at 13000 rpm for 30 min at 4 ℃. Protein concentration was determined (DC Protein Bioassay Kit, Bio-Rad). GSH assays were performed in a final volume of 25 μL with 2.0 μM of the probe. For inhibition assays (with NEM or **WT**), cell lysates were pretreated with NEM (1 mM) for 20 min, or **WT** (100 μM) for 2 h, respectively, then incubated with the probe before fluorescence measurements were recorded. For GSH assays carried out in mammalian cell lysates, since the endogenous GSH concentration in mammalian cells is relatively high (> 1 mM), and a large amount of cell lysates (200 μg/mL) were used in the reaction, we fitted the reaction between the probe and the lysates to pseudo-first order kinetics, and obtained the pseudo-first order reaction constant *k*' in unit s^{-1} by using equation (1). The plot of *k*' versus concentration of cell lysate (50, 100, 150 and 200 μg/mL) was used to define reaction constant *k* (**Table 1** & **Supplementary Fig. S2e**) by using equation (2).

$$r = k[\text{probe}][\text{lysate}] = k'[\text{probe}] \qquad \text{equation (1)}$$

$$k' = k[\text{lysate}] \qquad \text{equation (2)}$$

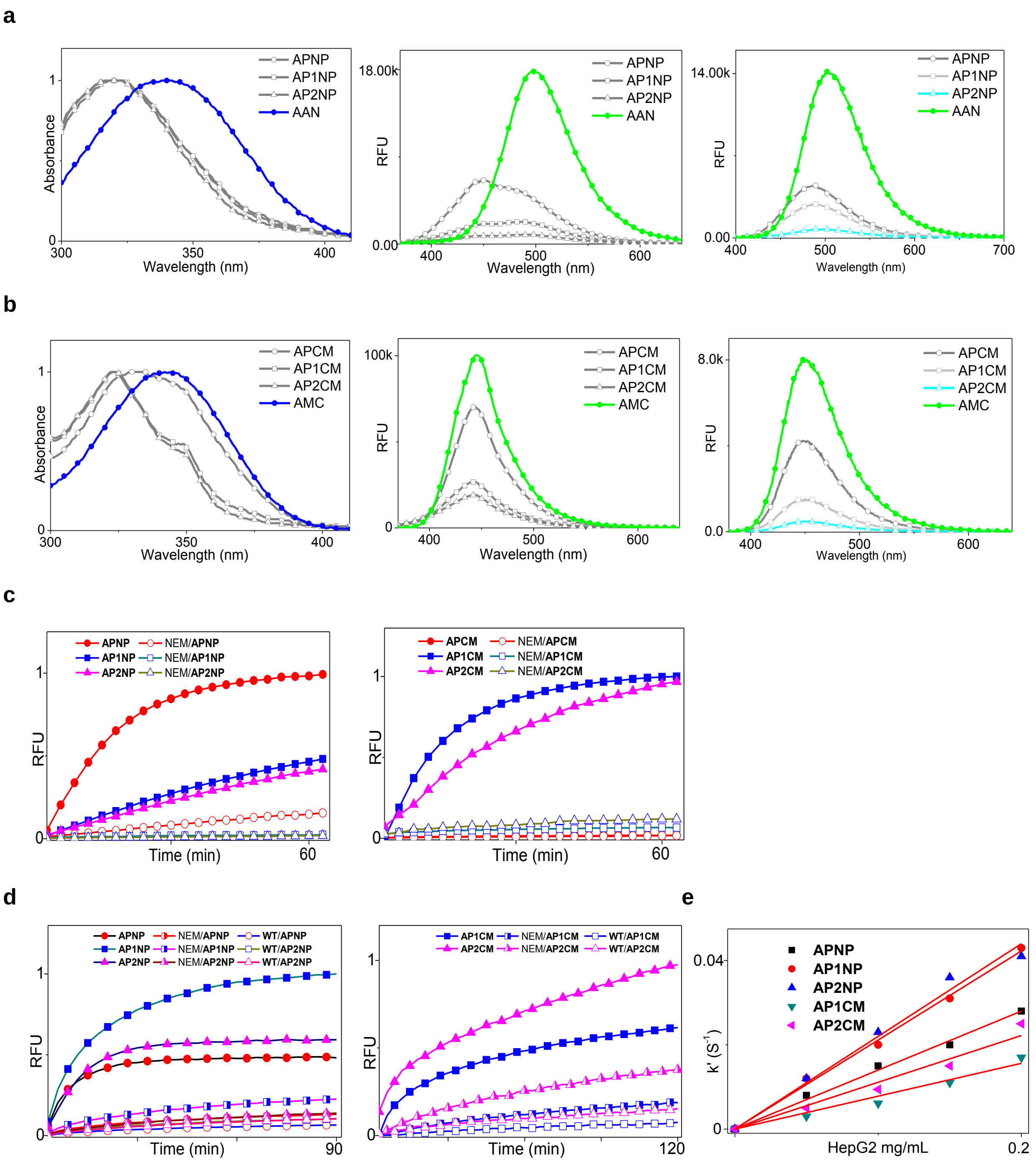

Supplementary Figure S1. **a**, Normalized spectra of 1P absorption (left), 1P fluorescence emission (middle), and 2P fluorescence emission (right) of 1.0 μM **APNP/AP1NP/AP2NP/AAN** in PBS buffer (*p*H 7.5, supplemented with 0.02 % Triton X-100. **b**, Normalized spectra of 1P absorption (left), 1P fluorescence emission (middle), and 2P fluorescence emission (right) of 1.0 μM **APCM/AP1CM/AP2CM/AMC** in the same PBS buffer. **c**, Normalized time-dependent microplate-based assay of 2.0 μM of different probes (left: **APNP**/**AP1NP**/**AP2NP**; right: **AP1CM**/**AP2CM**) against 300 μM GSH, with and without pretreatment of NEM (1 mM; 20 min), at room temperature in PBS buffer. **d**, Normalized time-dependent microplate-based assay of 2.0 μM of different probes (left:

APNP/**AP1NP**/**AP2NP**; (right: **AP1CM**/**AP2CM**) against HepG2 lysates (200 μg/mL), with/without pretreatment of NEM (1 mM; 20 min) or **WT** (100 μM; 2 h), at room temperature in PBS buffer. **e**, Plot of secondary order reaction constant *k* (*R* = 0.997/0.998/0.993/0.984/0.971, respectively) as summarized in **Table 1** in the maintext. Assays were performed with 2 μM of a probe against different amounts of HepG2 cell lysate at room temperature in PBS buffer.

Supplementary Table S1. Photophysical properties of fluorophores and probes in PBS buffer. Other data are shown in **Table 1**.

Probe	λ/nm[a]	$\varepsilon \times 10^{-3}$[b]
**AAN**	340/500	7.97
**APNP**	320/450	7.97
**AP1NP**	320/485	6.54
**AP2NP**	320/485	6.69
**AMC**	345/445	9.92
**APCM**	330/445	7.07
**AP1CM**	325/445	8.50
**AP2CM**	325/445	9.77

[a] Peak position of the longest absorption/emission bands. [b] Molar absorption coefficient of probes/fluorophores.

4. XTT Cell Proliferation Assay

The XTT colorimetric cell proliferation kit (Roche) was used with HepG2/Human lung adenocarcinoma epithelial (A549) cells, as previously described[1-3]. Briefly, cells were cultured in DMEM medium supplemented with 10% FBS in a humidified 5% CO_2 incubator at 37 ℃ until they reached 20 ~ 30% confluence (since they will reach 80 ~ 90% within 48 to 72 h in the absence of the compound) in 96-well plates under the conditions described above. The medium was aspirated, washed with PBS, then treated, in duplicate, with 0.1 mL of the medium containing different concentrations of the probes (1 ~ 100 μM). The probes were applied from DMSO stocks whereby DMSO never exceeded 1% in the final solution. The same volume of DMSO was used as a negative control. Staurosporine (STS, 200 nM) was used as a positive control. After a total treatment time of 24 h, proliferation of cells was measured (read at 450 nm, ref. at 650 nm; **Supplementary Fig. S2**).

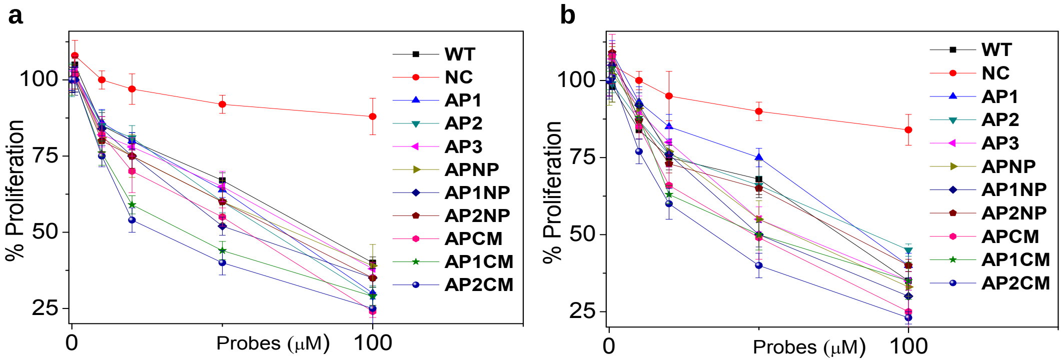

Supplementary Figure S2. Cell proliferation assay profiles of different probes against **a**, HepG2 and **b**, A549 cells.

5. *In Situ* Proteome Profiling

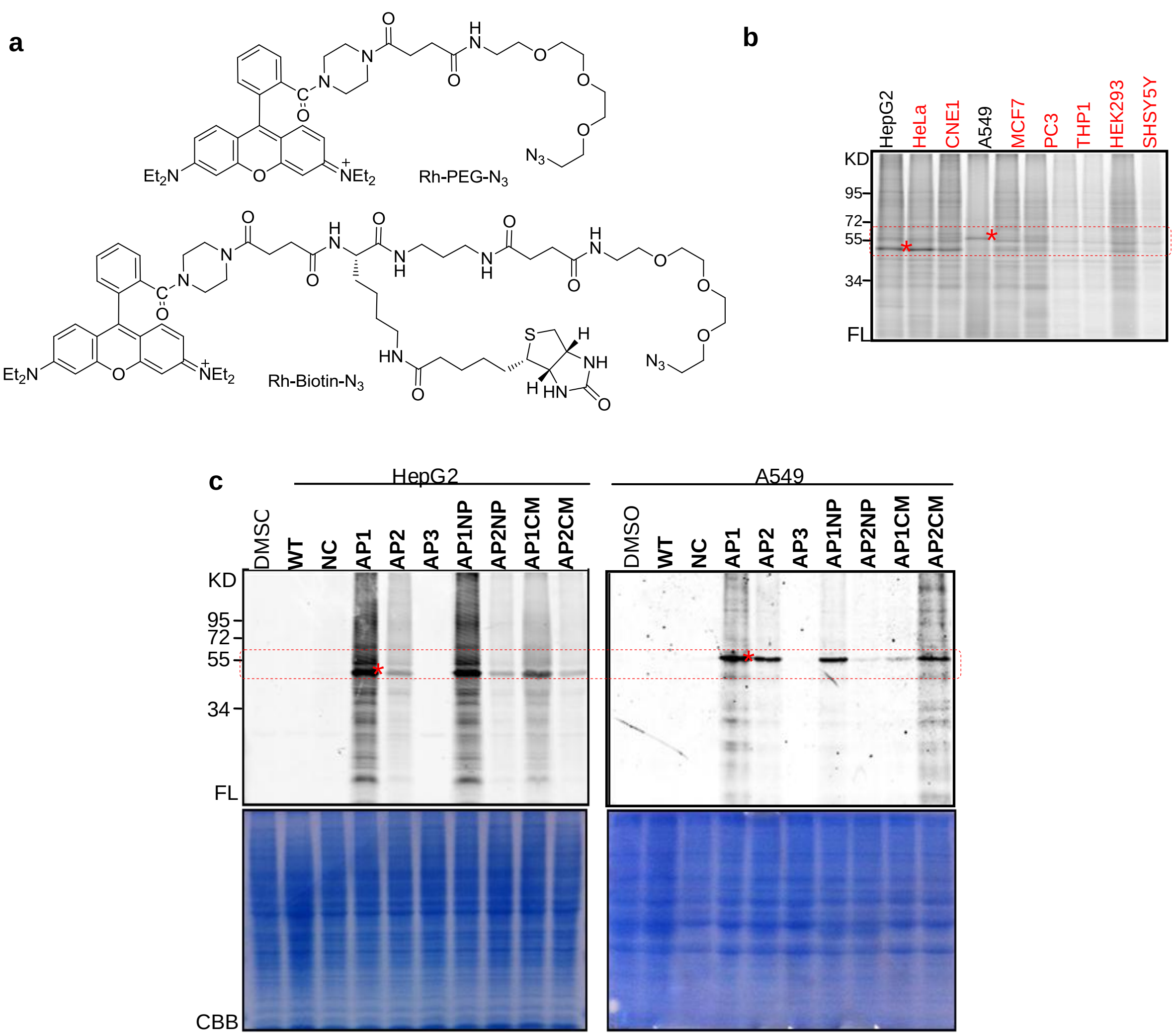

Supplementary Figure S3. a, Structures of the two different azide reporters used in the present study[4-8]. **b**, Contrast-adjusted in-gel fluorescence scanning profile of **Fig. 3a** from the maintext. **c**, In-gel fluorescence scanning

showing the proteome reactivity profiles of live HepG2 and A549 cells labeled by different probes (10 μM; 3 h at 37 ℃). FL = in-gel fluorescence scanning; CBB = coomassie gel. (*) and boxed (in red): see **Fig. 3** legend for details.

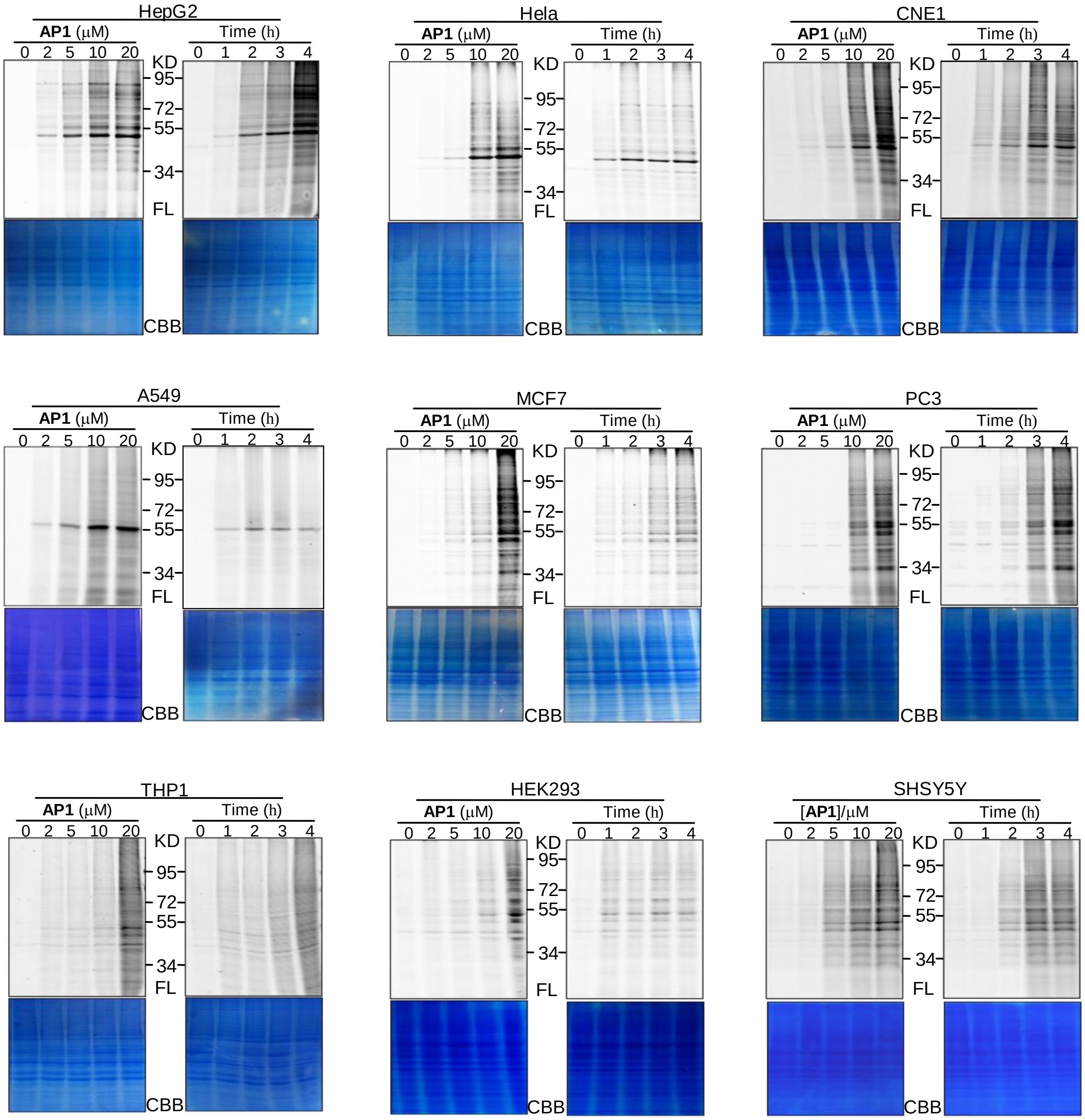

Supplementary Figure S4. In-gel fluorescence scanning images of concentration- and time-dependent labeling of 9 different live mammalian cells with **AP1**. (Left gels): different concentration of **AP1** (0 ~ 20 μM; 3 h at 37 ℃) were used. (Right gels): cells were incubated at 37 ℃ for different periods of time (0 ~ 4 h) with **AP1** (10 μM).

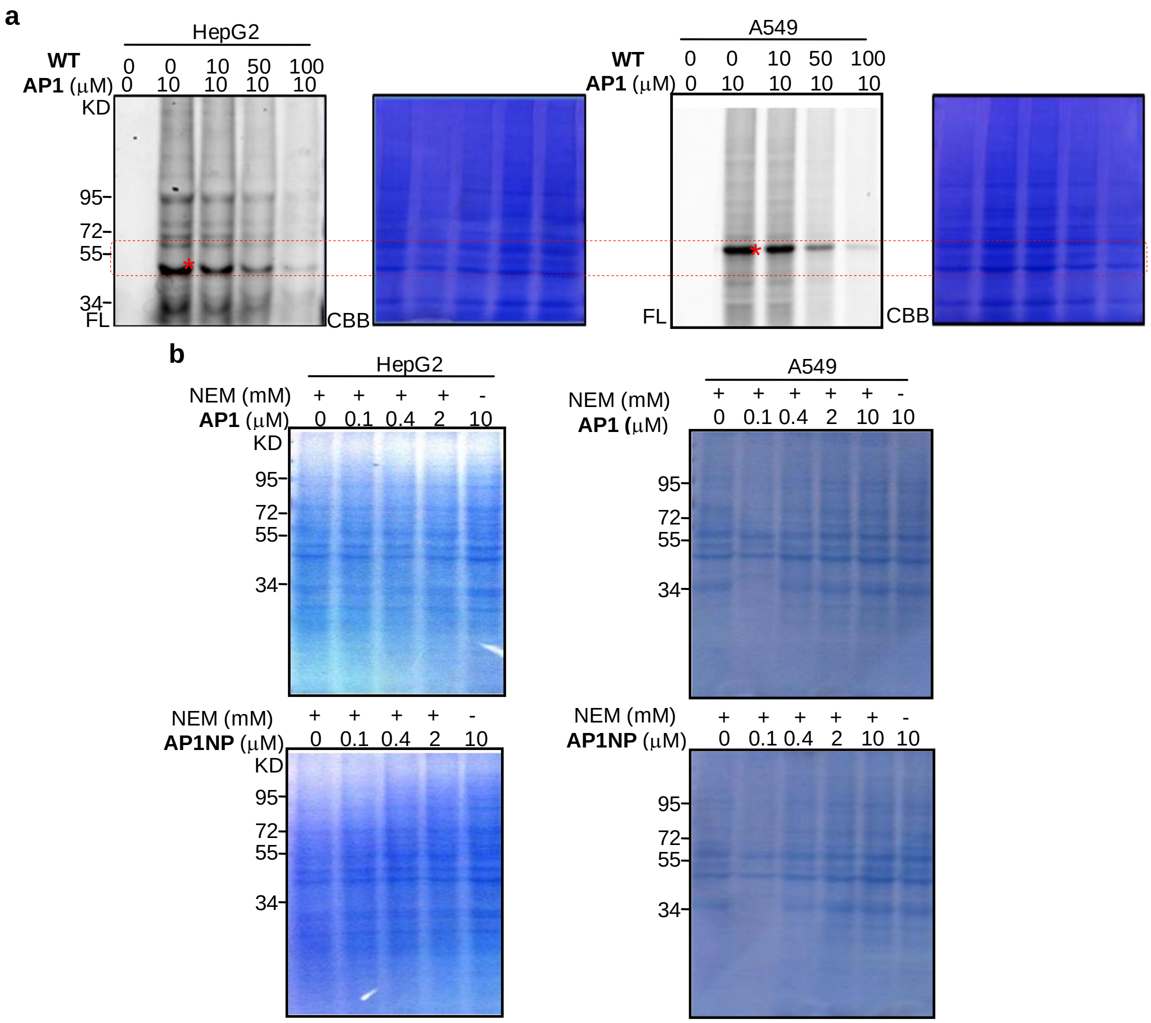

Supplementary Figure S5. a, In-gel fluorescence images showing *in situ* competitive proteome labeling profiles of **AP1** (10 μM, 3 h) in the presence of **WT** (0 ~ 100 μM; 1 h prior to incubation with probes). The corresponding coomassie gels are shown. **b**, The corresponding coomassie gels of the 4 fluorescent gels shown in **Fig. 3b/c**. (*) and boxed (in red): see **Fig. 3** legend for details.

6. One- and Two-photon Imaging of Mammalian Cells and Tissues

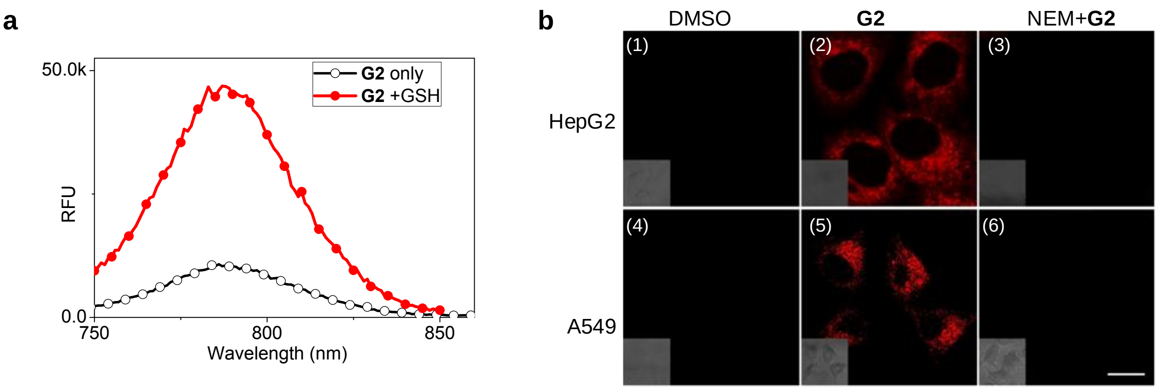

Supplementary Figure S6. a, 1P fluorescence spectra (λ_{ex} = 635 nm) of **G2** (5 μM) upon incubation with **GSH** (500 μM) at room temperature in PBS buffer (*p*H 7.5) for 20 min. **b**, 1P fluorescence images of HepG2 and A549 cells upon treatment with **G2** (20 μM; 20 min), with/without pre-treatment with NEM (1 mM; 20 min). (λ_{ex} = 635 nm; λ_{em} = 680 ~ 780 nm).

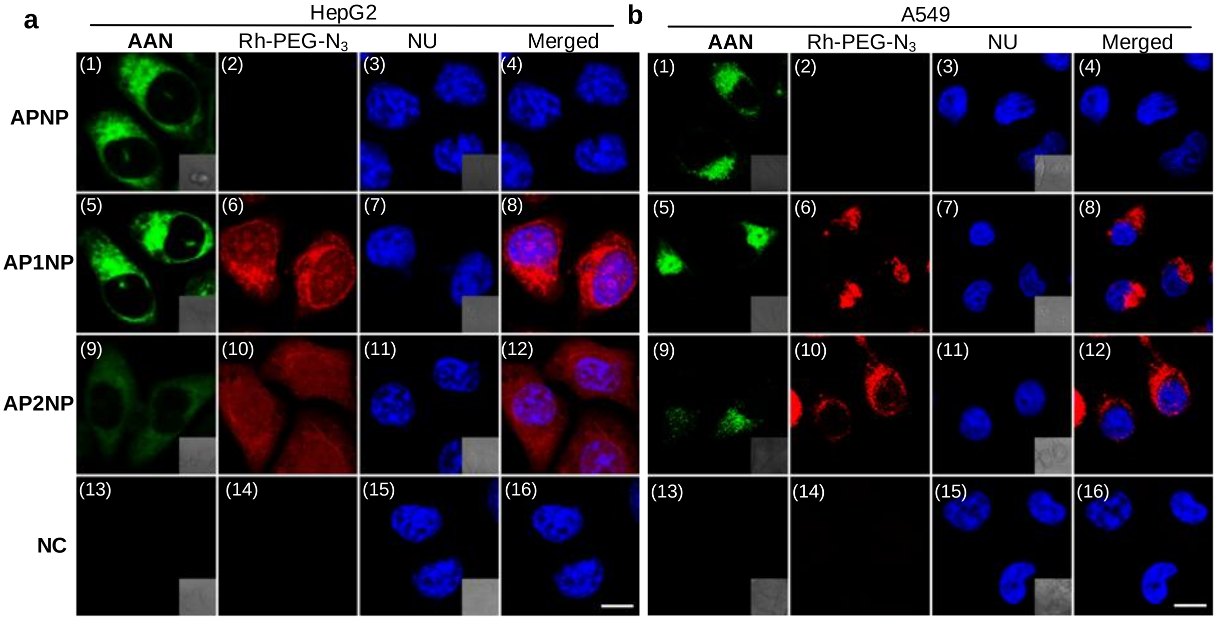

Supplementary Figure S7. 1P imaging of **a**, HepG2 and **b**, A549 cells upon pre-treatment with **APNP**/**AP1NP**/**AP2NP**/**NC** (10 μM; 3 h), followed by click chemistry with Rh-PEG-N_3 and nuclear staining (0.2 μg/mL; 10 min). The cells were imaged under **AAN** channel (λ_{ex} = 405 nm; λ_{em} = 500 ~ 550 nm), Rh-PEG-N_3 channel (λ_{ex} = 543 nm; λ_{em} = 560 ~ 600 nm), and NU channel (λ_{ex} = 405 nm; λ_{em} = 420 ~ 460 nm). Merged: merged images of panels (2)/(3), (6)/(7), (10)/(11) and (14)/(15), respectively. (Inserts): DIC images. Scale bar = 10 μm.

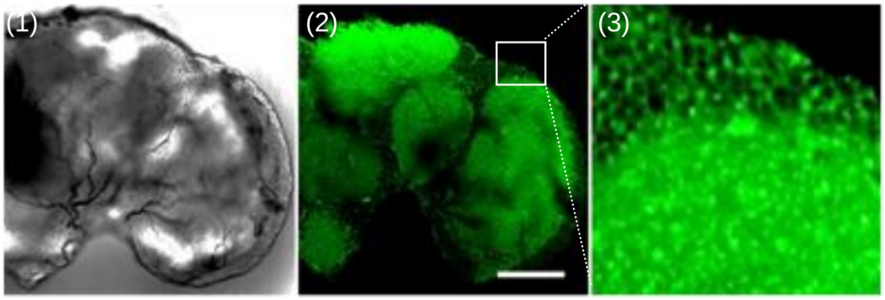

Supplementary Figure S8. 2P images of the fresh brain of 2-day-old live Drosophila treated with **APNP** (50 μM) for 3 h. The images was taken at 780 nm at a depth of ~100 μm with 20× magnifications (λ_{em} = 500 ~ 550 nm). (1) Bright- fieldimage; (2) Image of the brain 3 h after treatment with **APNP**. (3) 10 × magnified images of the boxed region in (2). Scale bar = 200 μm.

7. Target Identification/Validation

Upon *in situ* labeling of A549 cells by **AP1** (10 μM) as earlier described, pull-down (PD) experiments were similarly performed as previously described[4-8], by using Rh-Biotin-N_3 (**Supplementary Fig. S3a**). Negative control experiments were concurrently carried out on the cells by labeling with **NC** (10 μM) followed by click chemistry with Rh-Biotin-N_3. The PD samples were then separated by SDS-PAGE gels and subjected to silver staining (**Fig. 4a**). The strongly fluorescently-labeled band at ~60 kDa was excised from the gel. The same gel slice from the negative PD gel was similarly excised. The proteins were subjected to tryptic in-gel digest. Tryptic peptides were extracted from the gel and analyzed as previous described[4-8,10,11]. All peptide samples were dried in speedvac and reconstituted with 20 μL of 0.1% TFA/H_2O. The peptides were separated and analyzed on a Shimadzu UFLC system (Shimadzu, Japan) coupled to a LTQ-FT Ultra (Thermo Electron, Germany). Mobile phase A (0.1% formic acid in H_2O) and mobile phase B (0.1% formic acid in acetonitrile) were used to establish the 60 min gradient comprising 45 min of 5-35% B, 8 min of 35-50% B, and 2 min of 80% B, followed by reequilibrating at 5% B for 5 min. Peptides were then analyzed on LTQ-FT with an Advance CaptiveSpray Source (Michrom Bio Resources) at an electrospray potential of 1.5 kV. A gas flow of 2 L/min, ion transfer tube temperature of 180 ℃, and collision gas pressure of 0.85 m Torr were used. The LTQ-FT was set to perform data acquisition in the positive-ion mode as previously described[4-8,10,11], except that the m/z range of 350-1600 was used in the full MS scan. The raw data were converted to mgf format. The database search was performed with an in-

house Mascot server (version 2.2.07, Matrix Science) with MS tolerance of 10 ppm and MS/MS tolerance of 0.8 Da. Two missed cleavage sites of trypsin were allowed. Carbamidome-thylation (C) was set as a fixed modification, and oxidation (M) and phosphorylation (S, T, and Y) were set as variable modifications. The LC-MS/MS data were searched against the IPI (International Protein Index) human protein database using an in-house MASCOT server. All proteins were identified by a minimum score of 100 within the MS range 57 $\pm$ 4 kDa. Only those that appeared in triplicated samples were deemed as potential "hits". "False" hits that appeared in negative PD/LCMS experiments were further eliminated. Based on these criteria, a list of candidate hits was generated in **Fig. 3b** in the maintext. The original MS data were provided in SI_III.

For recombination protein labeling, recombinant NAMPT (from NAMPT colorimetric assay kit: CY-1251; MBL), vimentin (ab73843; abcam), PKA[12], PTP1B[13], Caspase 7[14], and PDI (Sigma, P3818) were used. ~50 ng of each protein was incubated with different concentrations of **AP1** (0 ~ 10 μM) for 1 h at room temperature with gentle mixing in a total of 50 μL PBS buffer. For competitive labeling experiments, ~50 ng of each protein was first pre-incubated with **WT** (100 μM) for 1 h, before addition of 10 μM probe. After click chemistry with Rh-PEG-N_3, the reaction was analyzed by 10% SDS-PAGE and in-gel fluorescence scanning (**Fig. 3f**) and silver staining **(Supplementary Fig. S11a**).

For western blotting (WB) analysis, the PD samples were resolved on 10% SDS-PAGE gels and transferred to a PVDF membrane and subsequently blocked with 2% BSA in PBST (0.1% Tween-20) for 1 h at room temperature. The primary antibodies ((1:1000 anti-NFκB p50; abcam), 1:2000 anti-ALDH1B1; GeneTex), (1:2000 anti-GSR; abcam,), (1:1000 anti-NAMPT; abcam)) were incubated for 1 h at room temperature followed by three times washing with PBST. Then HRP-conjugated anti-rabbit or mouse (1:5000; Pierce) was applied for 1 h at room temperature. The blot was developed by using the Dura Extended Duration Substrate (Thermo Scientific).

IC_{50} values for the inhibition of NAPTM by **WT, AP1** and **AP2** were obtained by the CycLex NAPTM colorimetric assay kit (CY-1251; MBL), based on the vendor's protocol. Recombinant NAMPT was incubated in 100 μL of the 2-step assay buffer-I solution, with varying concentrations of **WT**/**AP1**/**AP2** (or DMSO as negative control) for 3 h at 30 ℃ under gentle shaking (800 rpm). Then 20 μL of the 2-step assay buffer-II solutions were added to give a total volume of 120 μL. The activity was assayed spectrophotometrically by monitoring the

absorbance at 450 nm. All measurements were performed in triplicates. IC_{50} values were calculated from curve fitting using Boltzmann model with GraphPad Prism 5 (**Supplementary Fig. S11b**).

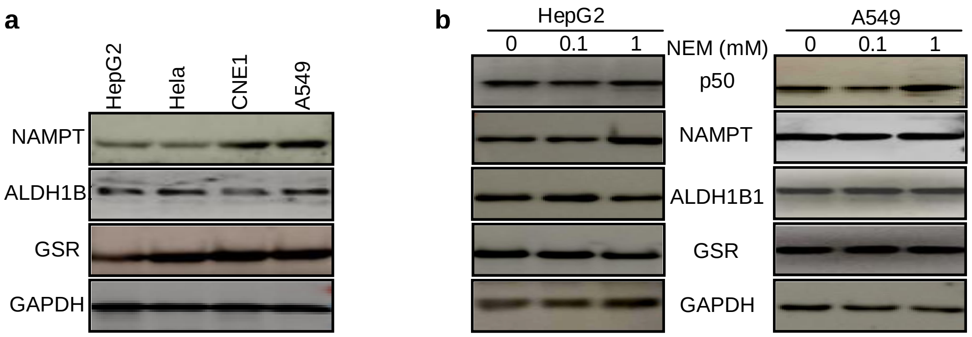

Supplementary Figure S9. a, WB (western blotting) determination of endogenous protein expression levels in 4 different mammalian cells. **b**, The same WB analysis of cells (HepG2 and A549) upon treatment with different concentrations of NEM (0, 0.1 & 1 mM; 20 min), indicating NEM treatment to cells didn't appreciably affect the endogenous expression level of these proteins. (GAPDH): loading controls.

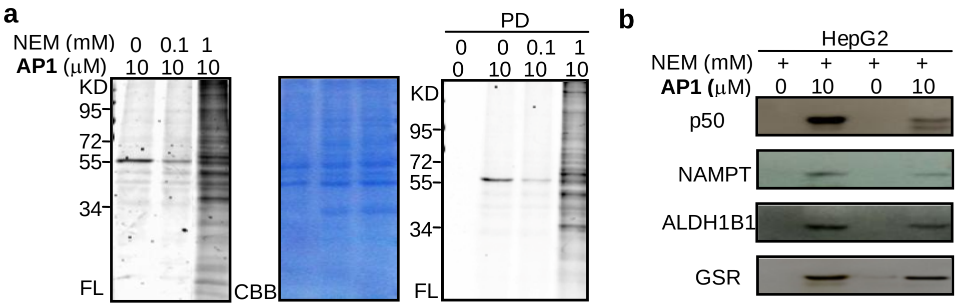

Supplementary Figure S10. a, In-gel fluorescence profiles of NEM (0, 0.1 & 1 mM; 20 min)-treated, **AP1** (10 μM; 3 h)-labeled A549 cells before (left two gels) and after PD (right). **b**, PD/WB target validation of NEM (1 mM; 20 min)-pretreated, **AP1** (10 μM; 3 h)-labeled HepG2 cells.

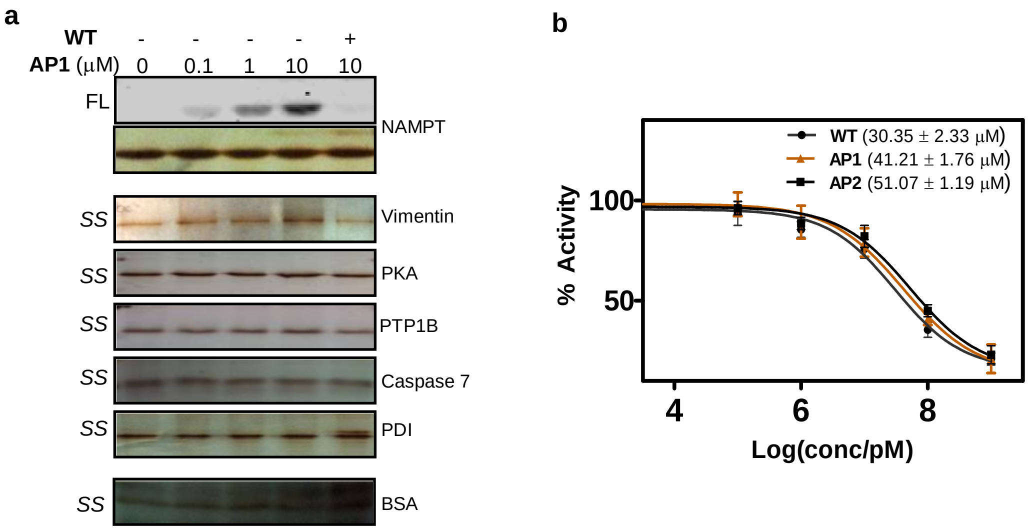

Supplementary Figure S11. a, In-gel fluorescence scanning images (FL) and silver staining (*SS*) of *in vitro* labeling of recombinant NAMPT/vimentin/PKA/PTP1B/Caspase 7/PDI/BSA (~50 ng) by different concentrations of **AP1** (0 ~ 10 μM; 1 h), with or without pretreatment of **WT** (100 μM; 1 h). Most fluorescent gels are provided in **Fig. 3f** in the maintext. **b**, IC_{50} plots of NAMPT inhibition by different amounts of **WT**/**AP1**/**AP2** (3 h at 30 °C).

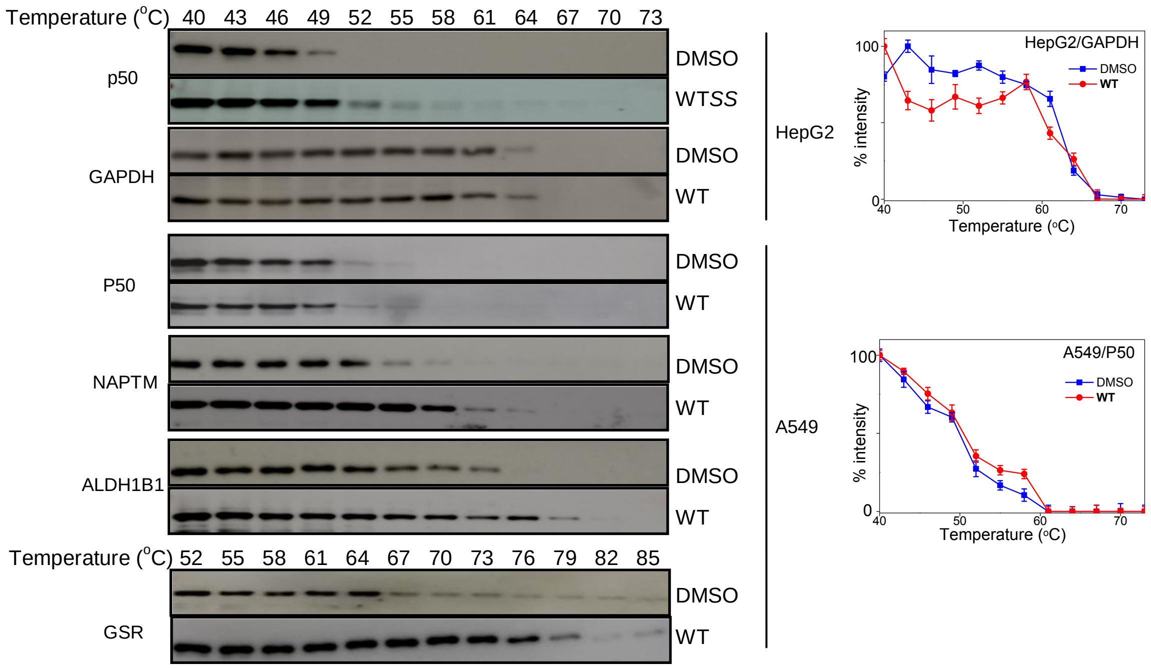

Supplementary Figure S12. WB results (left) and the corresponding graphical plots (right) of the CETSA assay carried out on HepG2/A549 cells upon treatment with **WT** (100 μM; 3 h; DMSO as negative control). Most graphical plots are provided in **Fig. 4d** in the maintext

8. Full-length blots

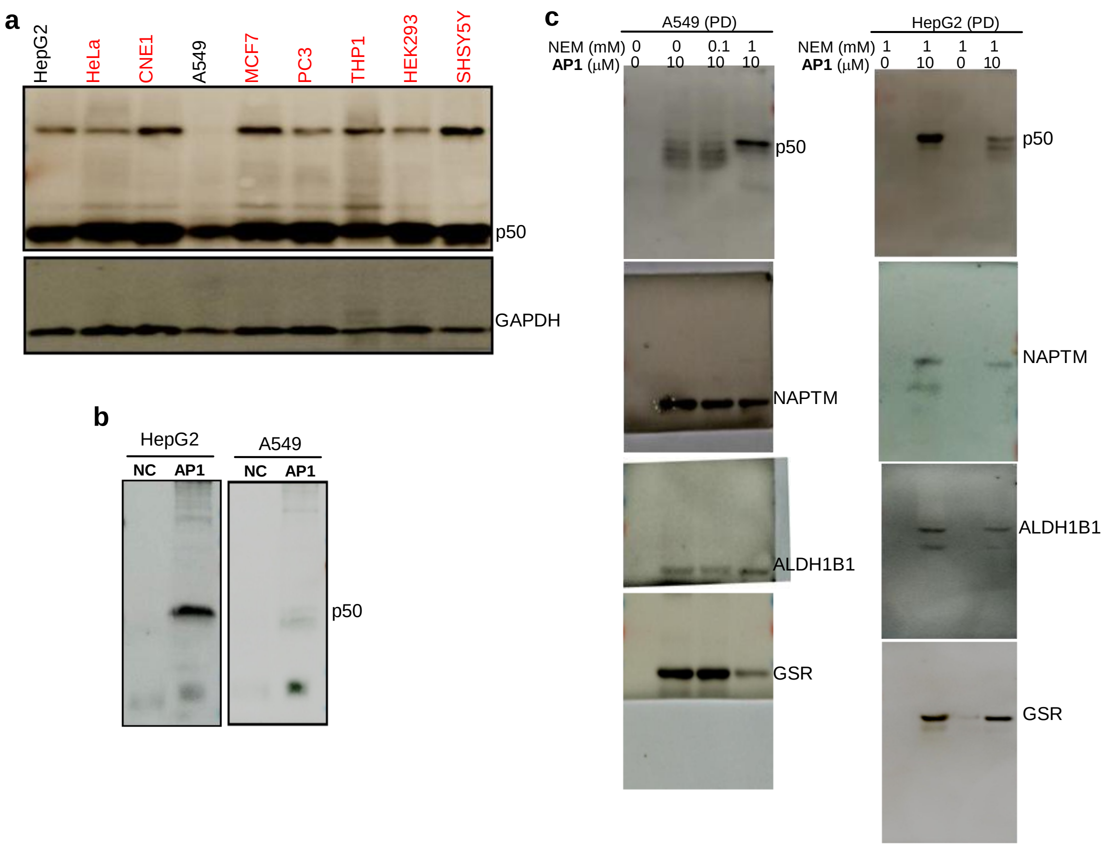

Supplementary Figure S13. Full-length blots for WB of **a**, Fig. 3a; **b**, Fig. 3b; **c**, Fig. 4c and **d**, **Supplementary Fig. 10b.**

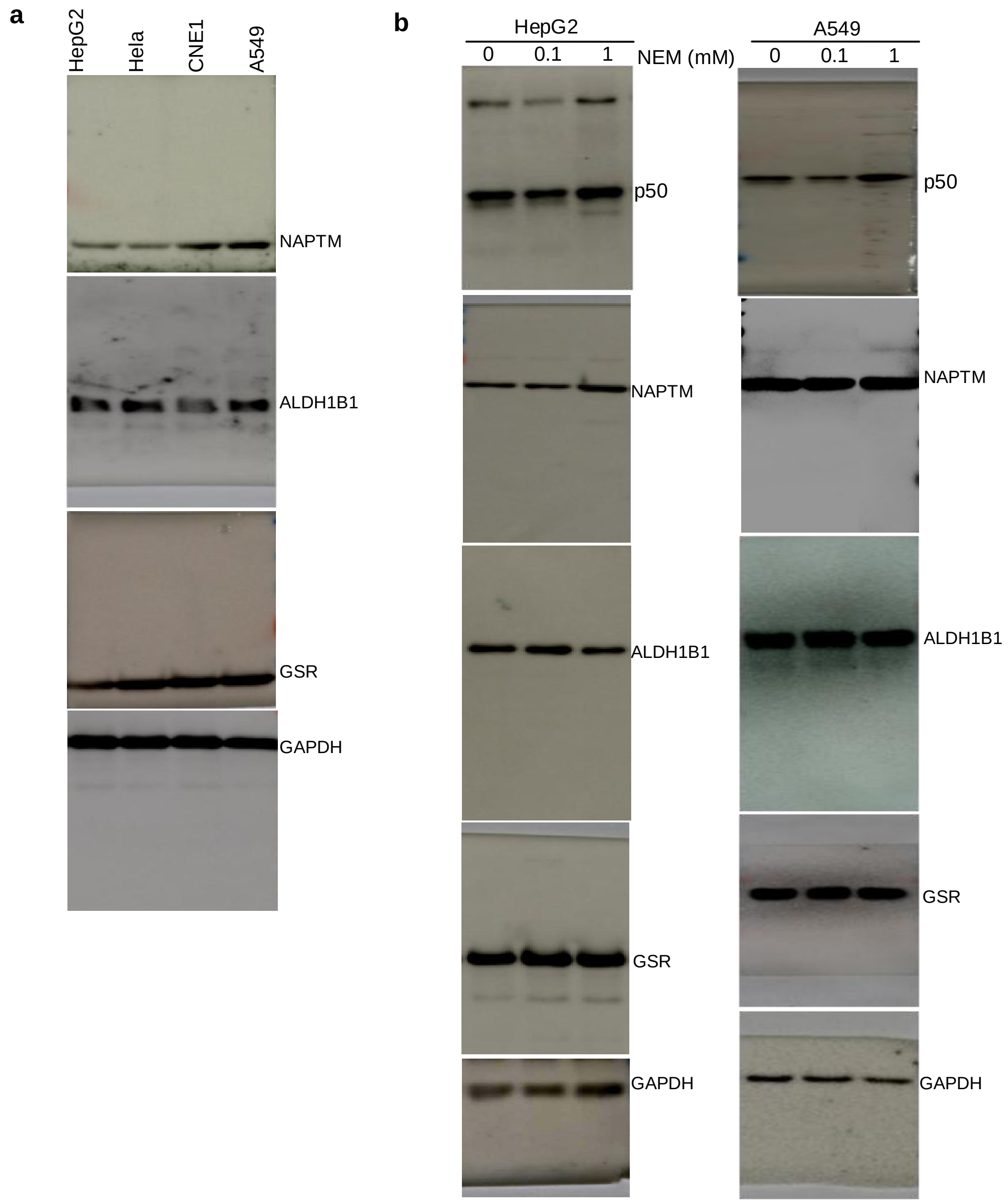

Supplementary Figure S14. Full-length blots for WB of **Supplementary Fig. S9**.

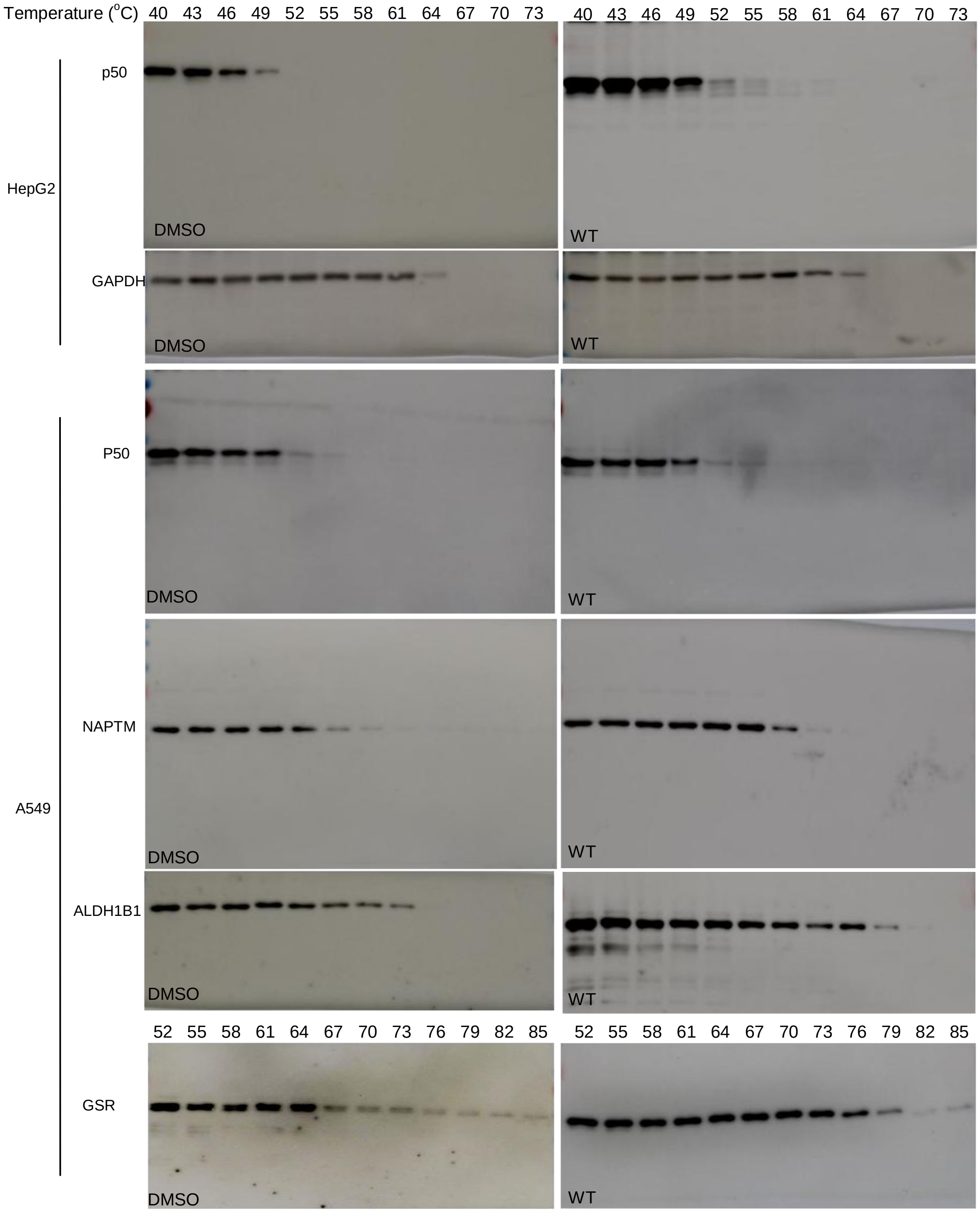

Supplementary Figure S15. Full-length blots for WB of **Supplementary Fig. S12**.

9. References

1. Li, L. *et al.* A sensitive two-photon probe to selectively detect monoamine oxidase B activity in Parkinson's disease models. *Nat. Commun.* **5,** 3276 (2014).

2. Li, L., Shen, X., Xu, Q.-H. & Yao, S. Q. A switchable two-photon membrane tracer capable of imaging membrane-associated protein tyrosine phosphatase activities. *Angew. Chem. Int. Ed.* **52,** 424-428 (2013).

3. Li, L., Ge, J., Wu, H., Xu, Q.-H. & Yao, S. Q. Organelle-specific detection of phosphatase activities with two-photon fluorogenic probes in cells and tissues. *J. Am. Chem. Soc.* **134,** 12157-12167 (2012).

4. Yang, P.-Y., Wang, M., He, C. Y. & Yao, S. Q. Proteomic profiling and potential cellular target identification of K11777, a clinical cysteine protease inhibitor, in *Trypanosoma brucei*. *Chem. Commun.* **48,** 835-837 (2012).

5. Yang, P.-Y. *et al.* Activity-based proteome profiling of potential cellular targets of Orlistat-an FDA-approved drug with anti-tumor activities. *J. Am. Chem. Soc.* **132,** 656-666 (2010).

6. Yang, P.-Y. *et al.* Design, synthesis and biological evaluation of potent azadipeptide nitrile inhibitors and activity-based probes as promising anti-*Trypanosoma brucei* agents. *Chem. Eur. J.* **18,** 6528-6541 (2012).

7. Shi, H., Cheng, X., Sze, S. K. & Yao, S. Q. Proteome profiling reveals potential cellular targets of staurosporine using a clickable cell-permeable probe. *Chem. Commun.* **47,** 11306-11308 (2011).

8. Shi, H., Zhang, C.-J., Chen, G. Y. J. & Yao, S. Q. Cell-based proteome profiling of potential dasatinib targets by use of affinity-based probes. *J. Am. Chem. Soc.* **134,** 3001-3014 (2012).

9. Yin, J. *et al.* Cyanine-based fluorescent probe for highly selective detection of glutathione in cell cultures and live mouse tissues. *J. Am. Chem. Soc.* **136,** 5351-5358 (2014).

10. Gan, C. S., Guo, T., Zhang, H., Kim, S. K. & Sze, S. K. A comparative study of electrostatic repulsion-hydrophilic interaction chromatography (ERLIC) versus SCX-IMAC-based methods for phosphopeptide isolation/enrichment. *J. Proteome Res.* **7,** 4869-4877 (2008).

11. Hao, P. *et al.* Novel application of electrostatic repulsion-hydrophilic interaction chromatography (ERLIC) in shotgun proteomics: comprehensive profiling of rat kidney proteome. *J. Proteome Res*. **9,** 3520-3526 (2010).

12. Cheng, X. Li, L. Uttamchandani, M. & Yao, S. Q. A tuned affinity-based staurosporine probe for in situ profiling of protein kinases. *Chem. Commun.* **50,** 2851-2853 (2014).

13. Srinivasan, R., Uttamchandani, M. & Yao, S. Q. Rapid assembly and in situ screening of bidentate inhibitors of protein tyrosine phosphatases. *Org. Lett.* **8,** 713-716 (2006).

14. Ng, S. L., Yang, P.-Y., Chen, K. Y.-T, Srinivasan, R. & Yao, S. Q. “Click” synthesis of small-molecule inhibitors targeting caspases. *Org. Biomol. Chem.* **6,** 844-847 (2008).

15. Jafari, R. *et al.* The cellular thermal shift assay for evaluating drug target interactions in cells. *Nat. Protoc.* **9,** 2100-2122 (2014).

^{1}H NMR and ^{13}C NMR:

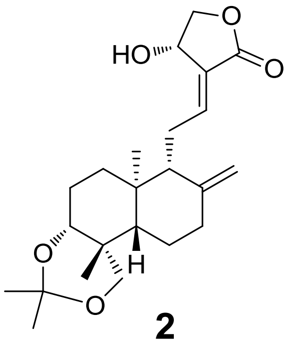

2

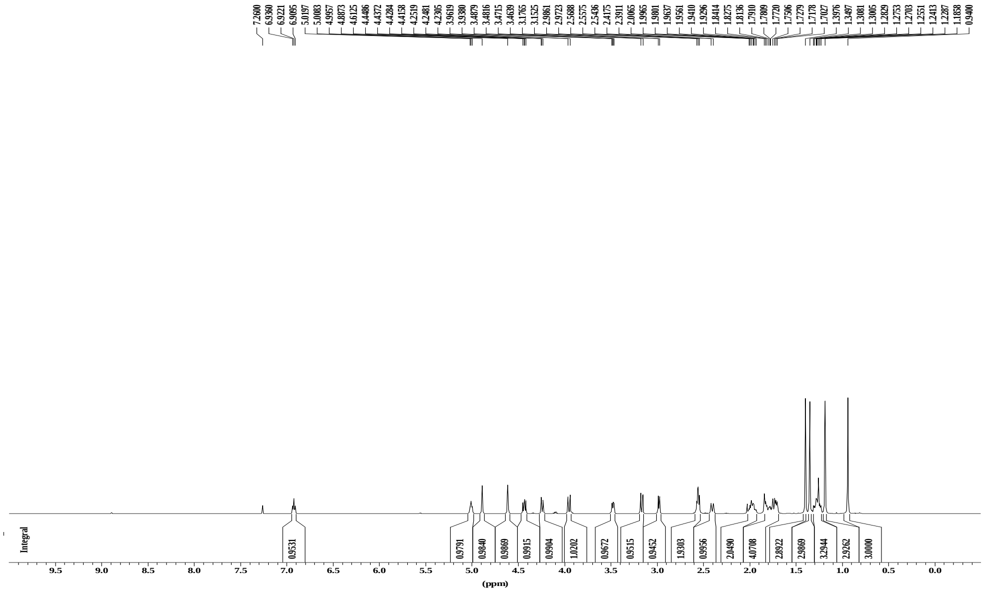

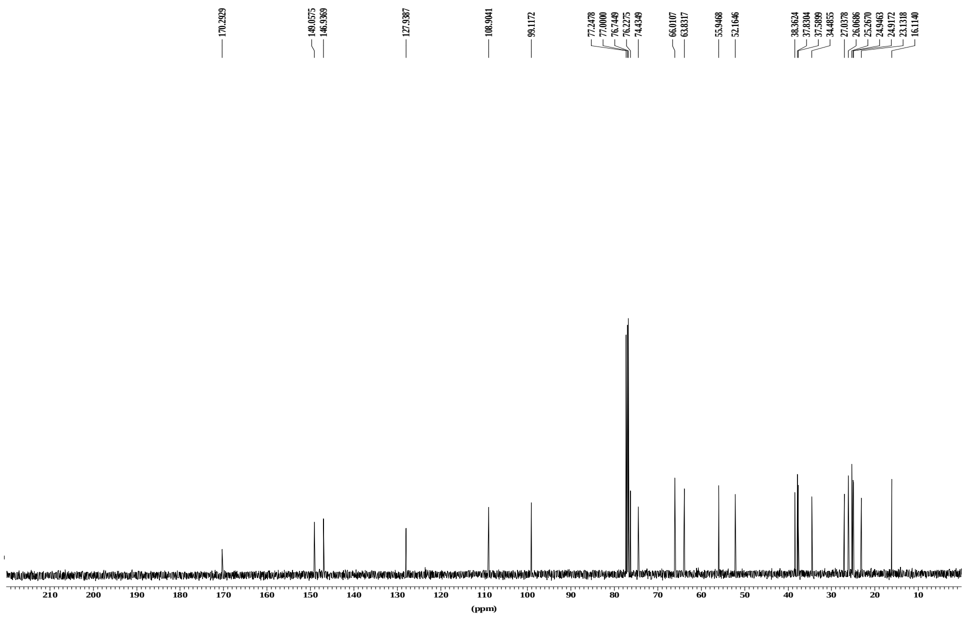

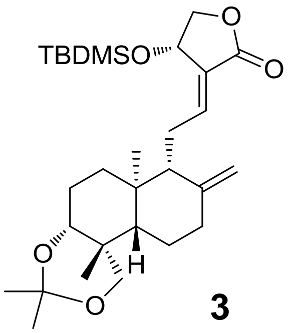

3

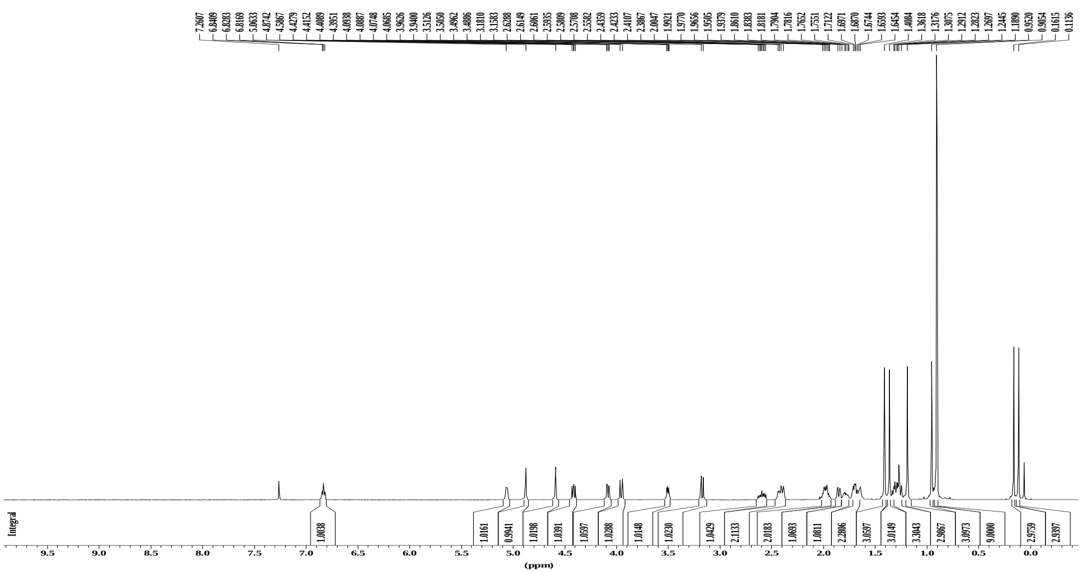

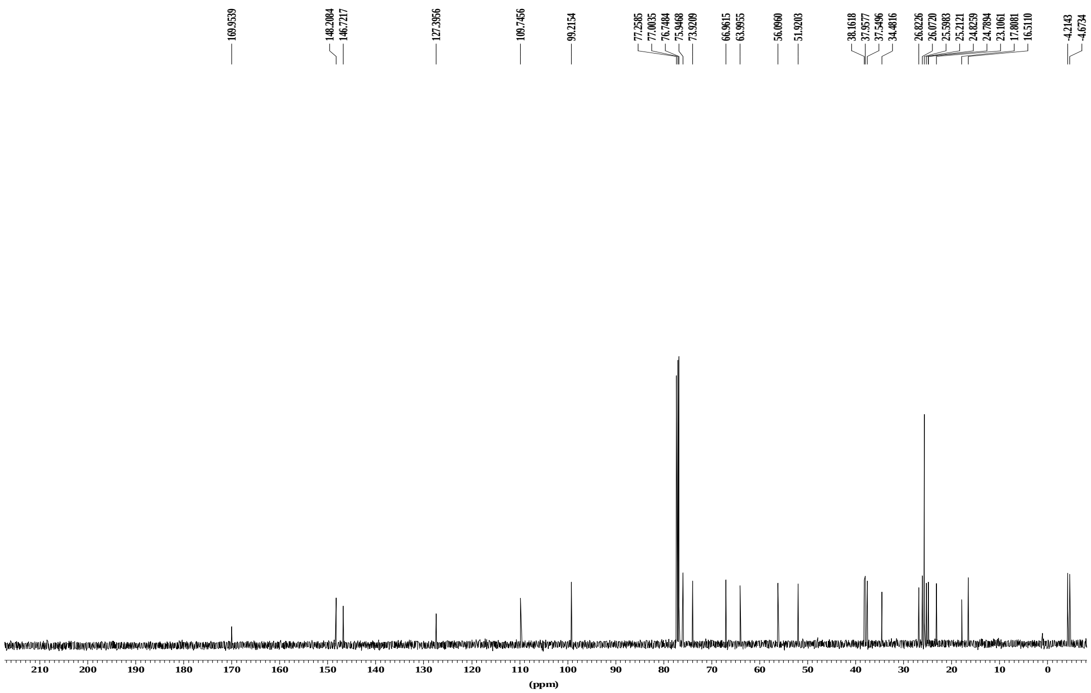

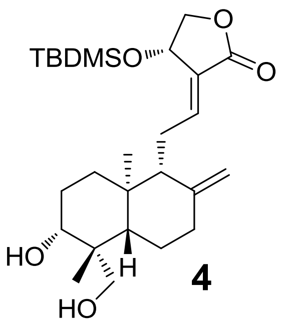

4

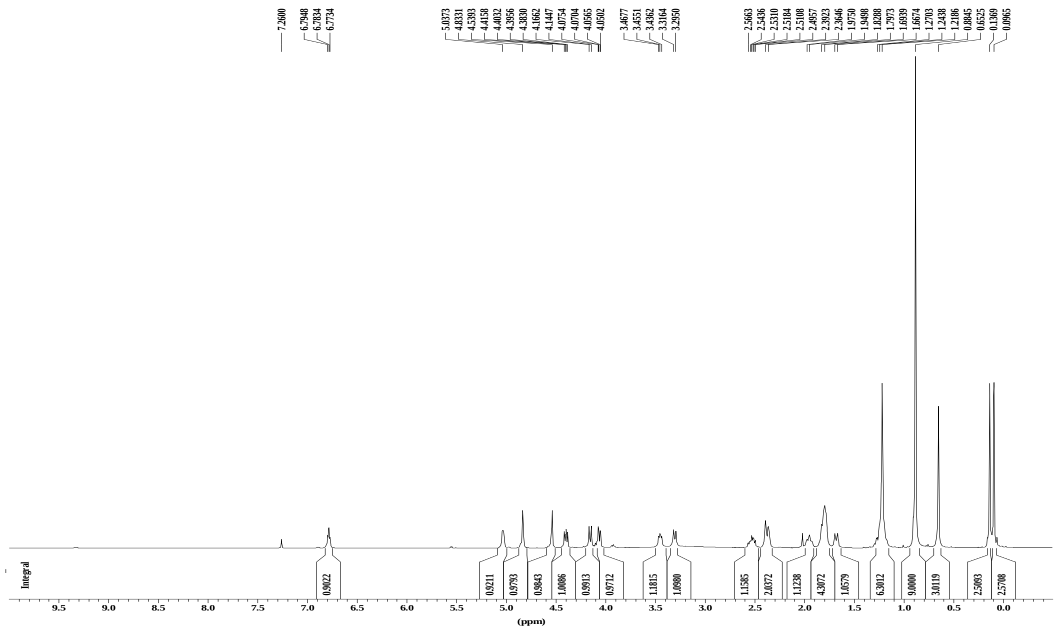

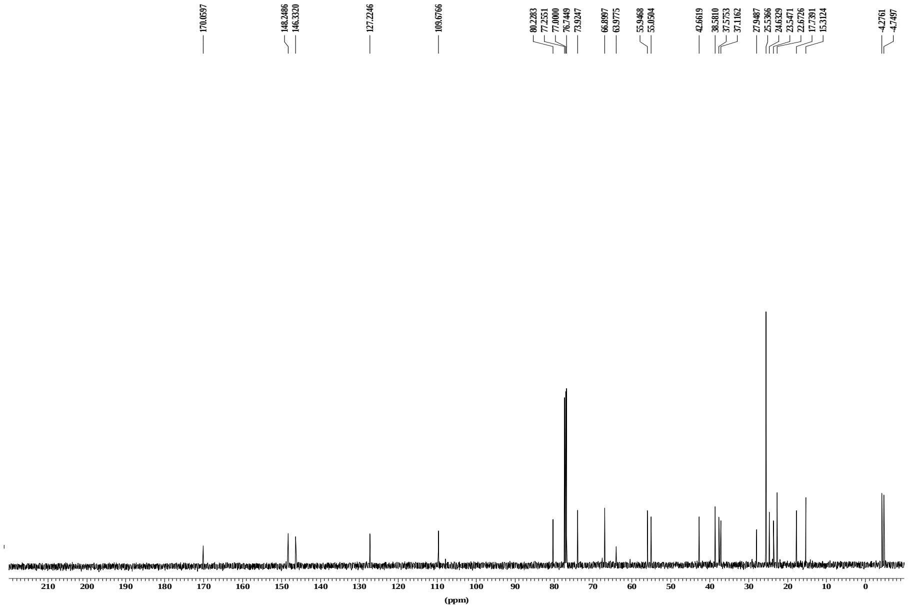

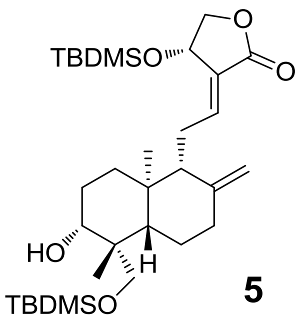

5

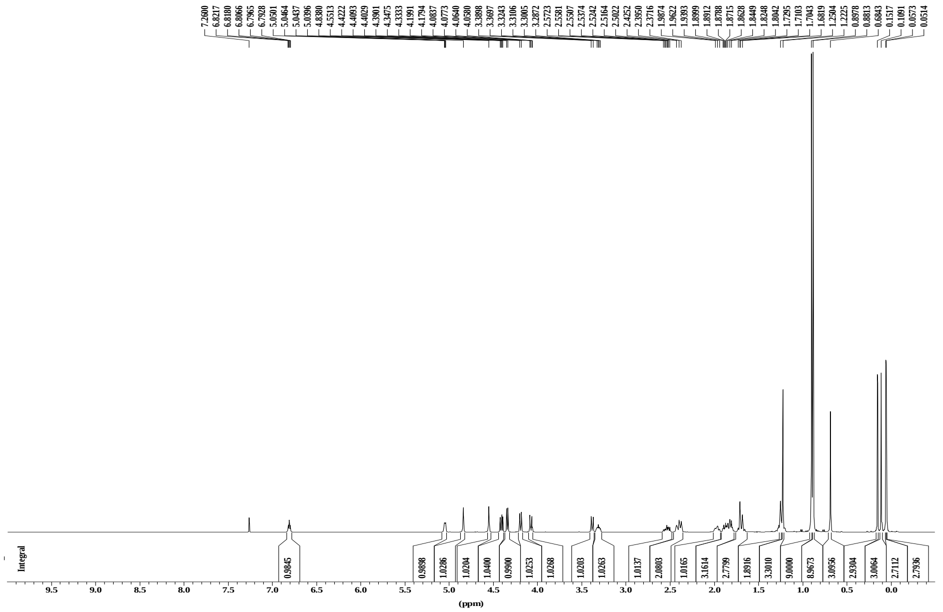

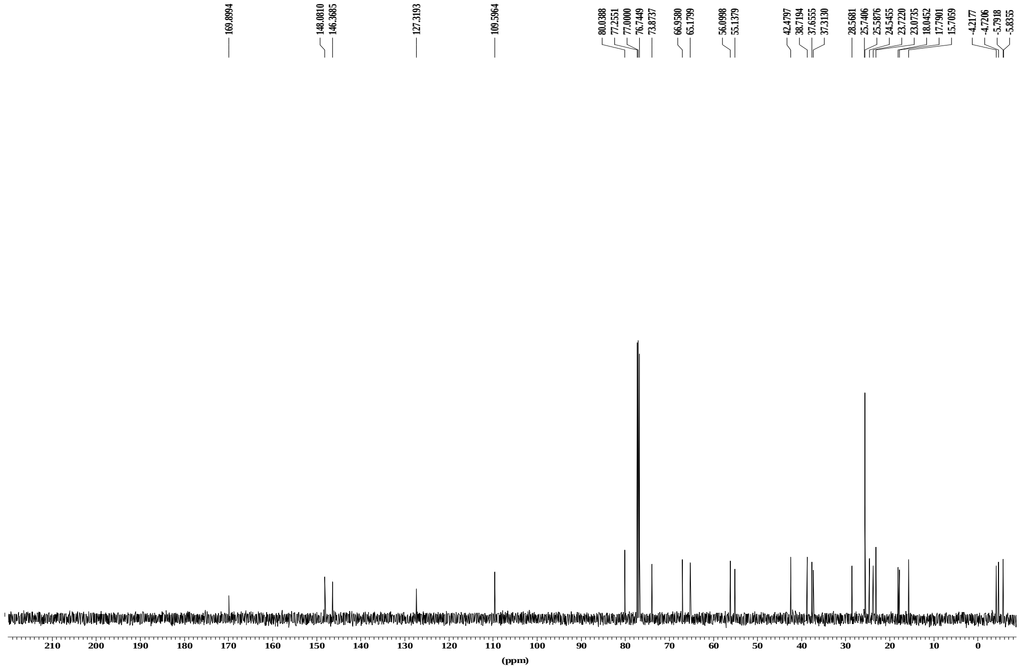

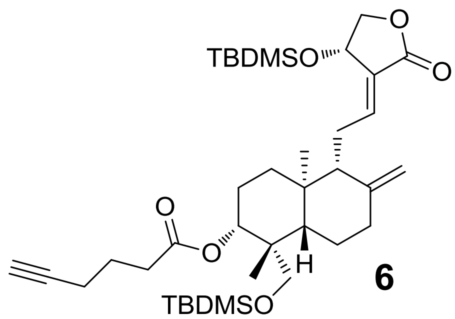
TBDMSO
TBDMSO
H
6

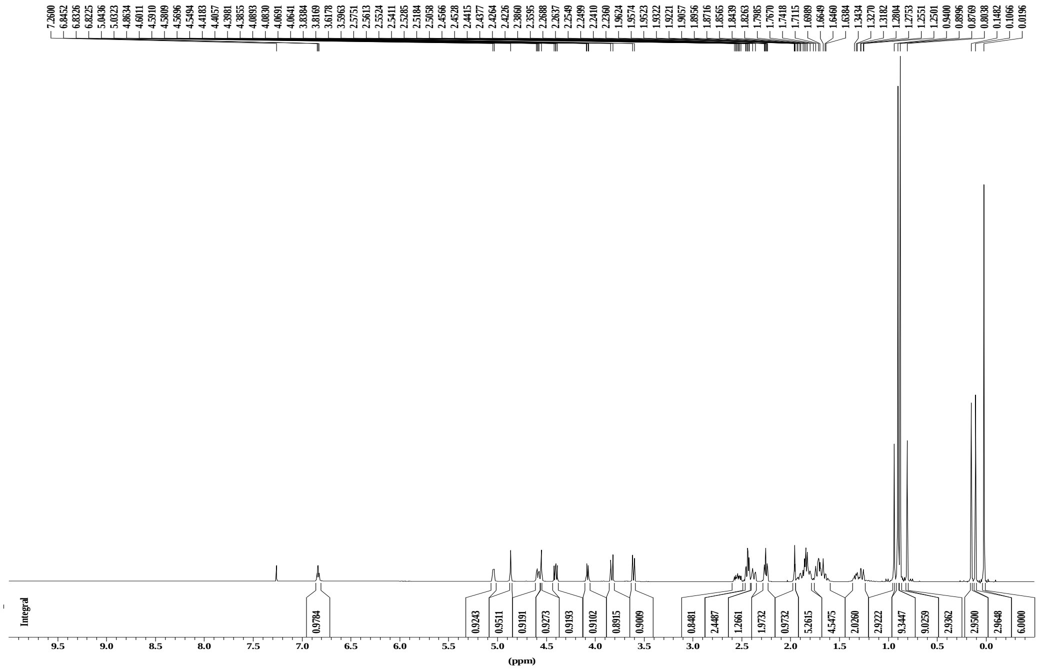
Integral
(ppm)

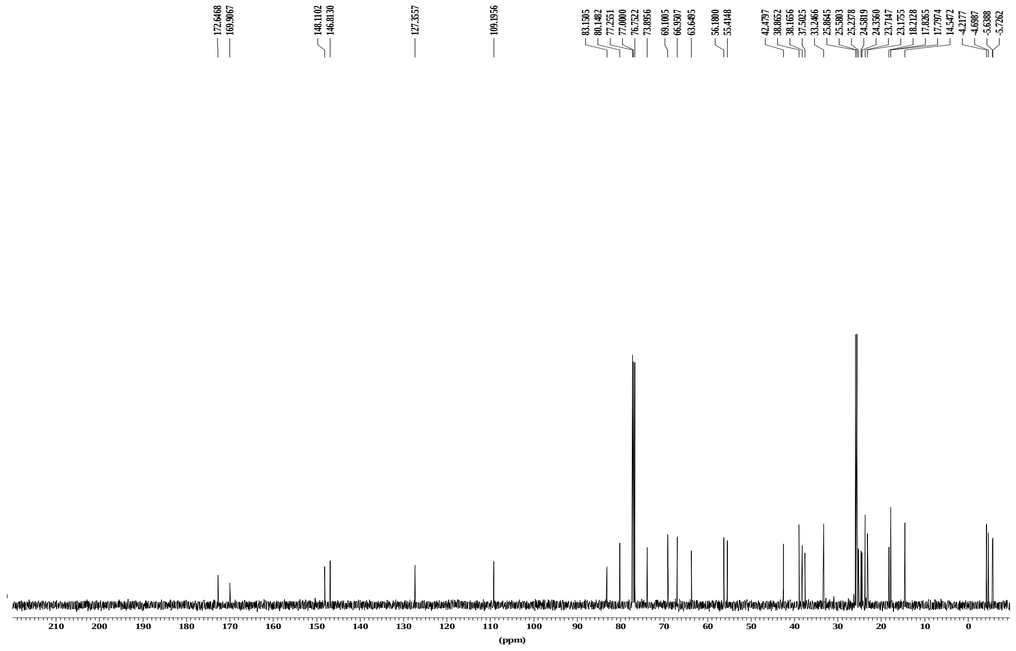
(ppm)

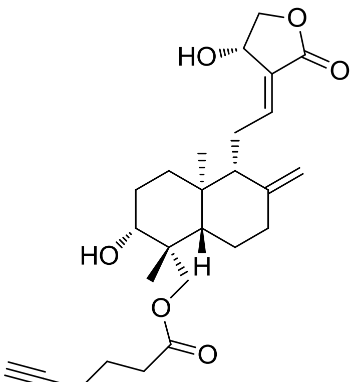

AP1

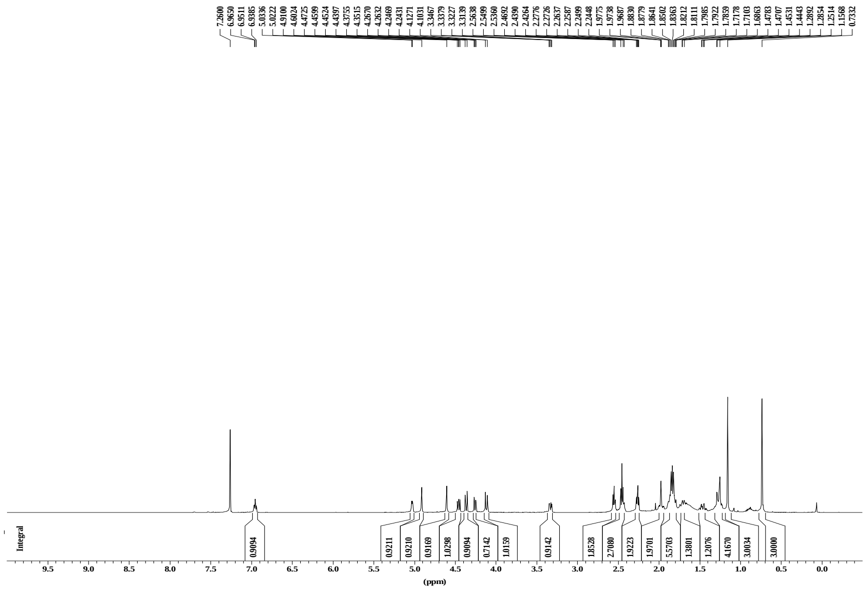

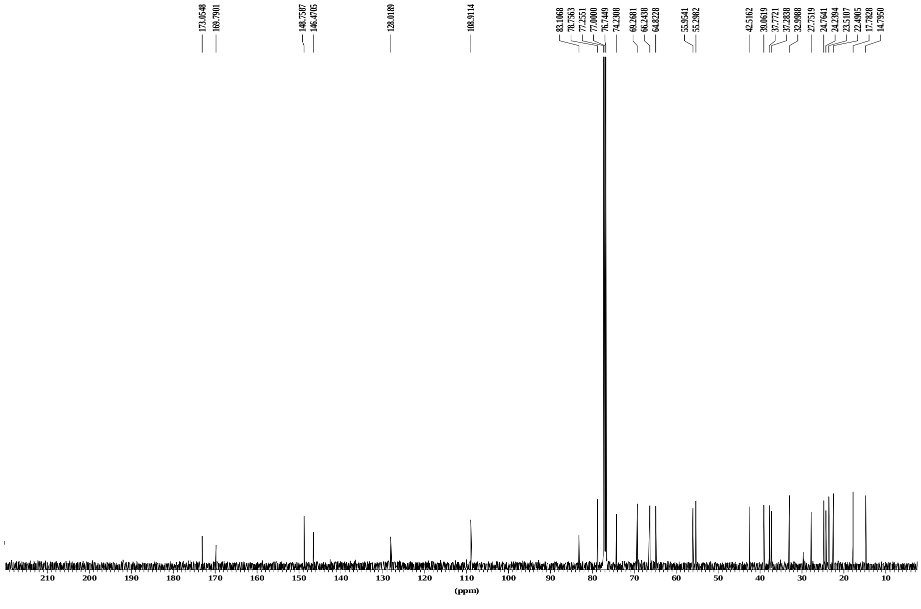

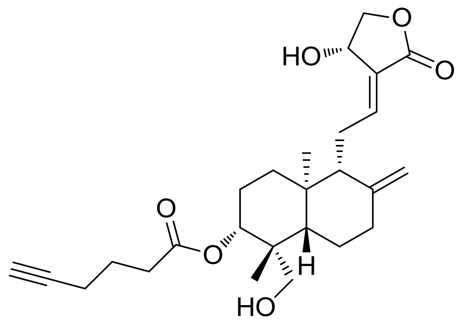
O
HO
O
O
O
H
HO

AP2

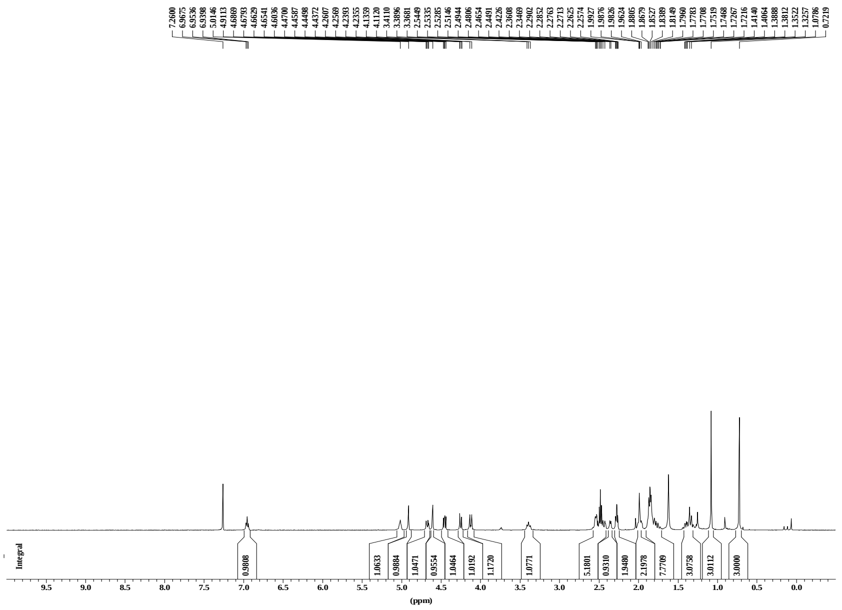
7.2600
6.9675
6.9536
6.9398
5.0146
4.9113
4.6869
4.6793
4.6629
4.6541
4.6036
4.4700
4.4587
4.4498
4.4372
4.2607
4.2569
4.2393
4.2355
4.1339
4.1120
3.4110
3.3896
3.3681
2.5449
2.5335
2.5285
2.5146
2.4944
2.4806
2.4654
2.4491
2.4226
2.3688
2.3469
2.2902
2.2852
2.2763
2.2713
2.2625
2.2574
1.9927
1.9876
1.9826
1.9624
1.8805
1.8679
1.8527
1.8389
1.8149
1.7960
1.7783
1.7708
1.7519
1.7468
1.7267
1.7216
1.4140
1.4064
1.3888
1.3812
1.3522
1.3257
1.0786
0.7219
Integral
0.9808
1.0633
0.9884
1.0471
0.9554
1.0464
1.0192
1.1720
1.0771
5.1801
0.9310
1.9480
2.1978
7.7709
3.0758
3.0112
3.0000
9.5
9.0
8.5
8.0
7.5
7.0
6.5
6.0
5.5
5.0
4.5
4.0
3.5
3.0
2.5
2.0
1.5
1.0
0.5
0.0
(ppm)

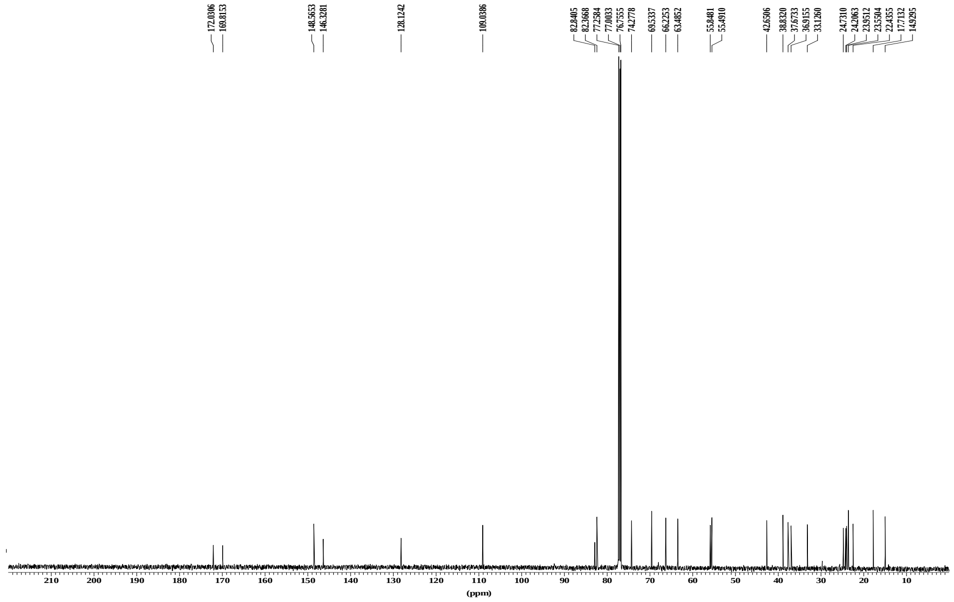
172.0306
169.8153
148.5653
146.3281
128.1242
109.0386
82.8405
82.3668
77.2584
77.0033
76.7555
74.2778
69.5337
66.2253
63.4852
55.8481
55.4910
42.6506
38.8320
37.6733
36.9155
33.1260
24.7310
24.2063
23.9312
23.5504
22.4355
17.7132
14.9295
210
200
190
180
170
160
150
140
130
120
110
100
90
80
70
60
50
40
30
20
10
(ppm)

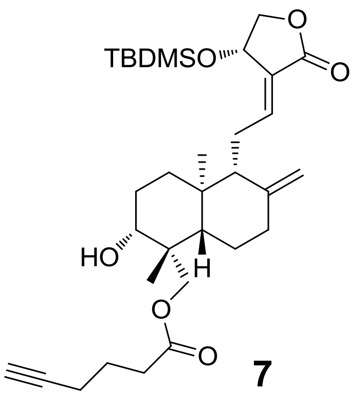

7

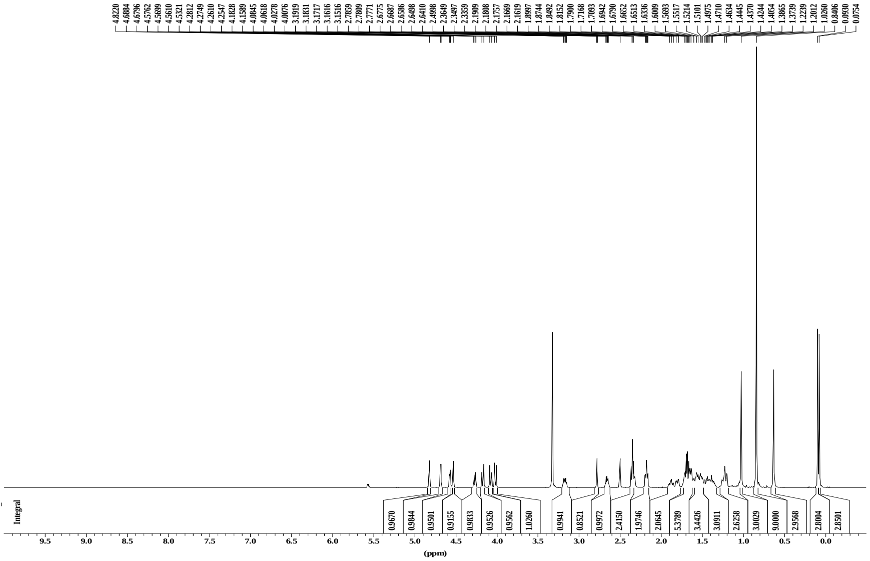

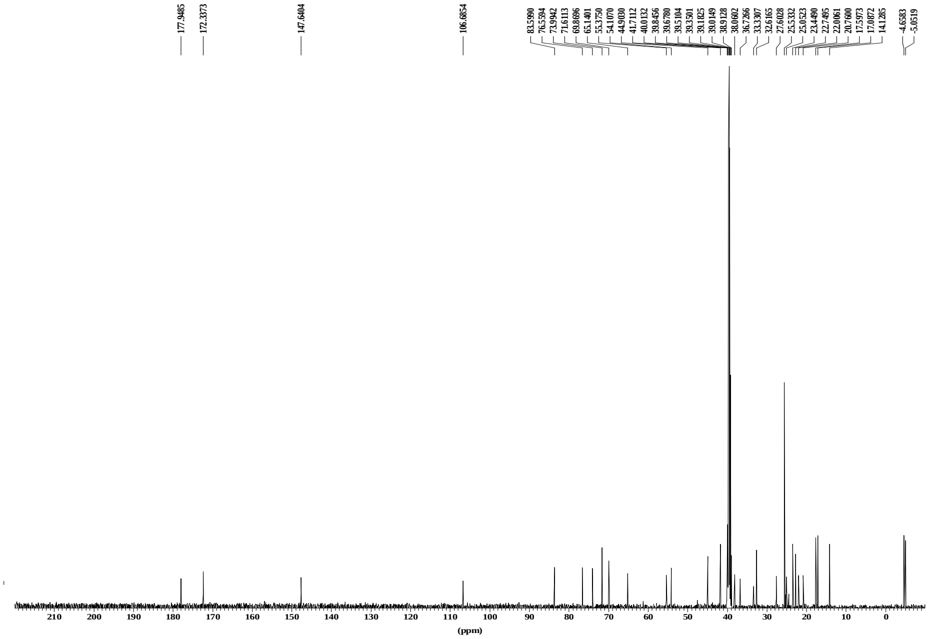

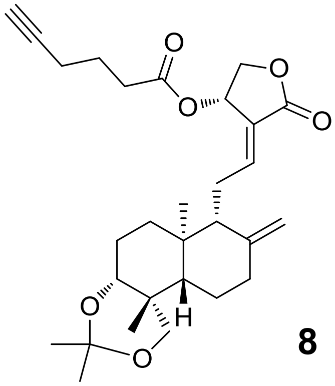

8

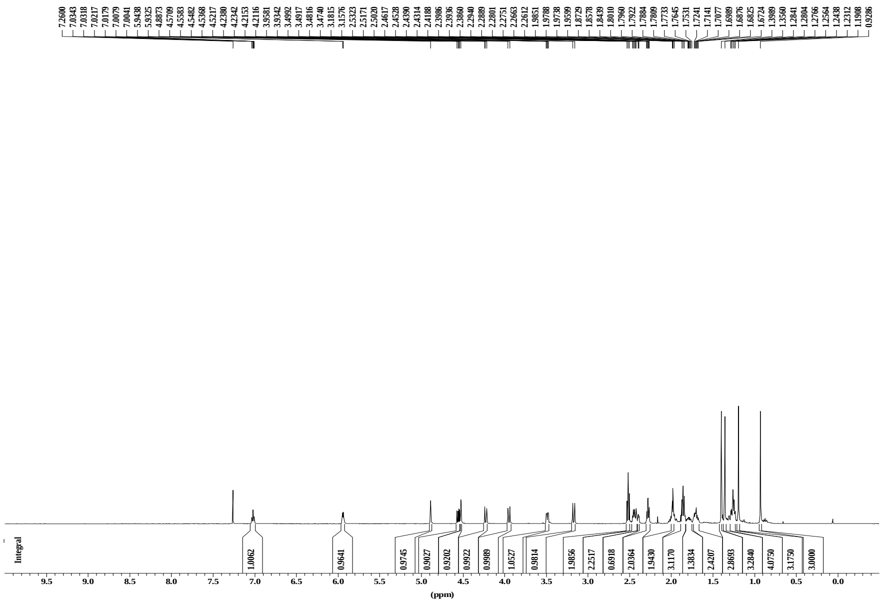

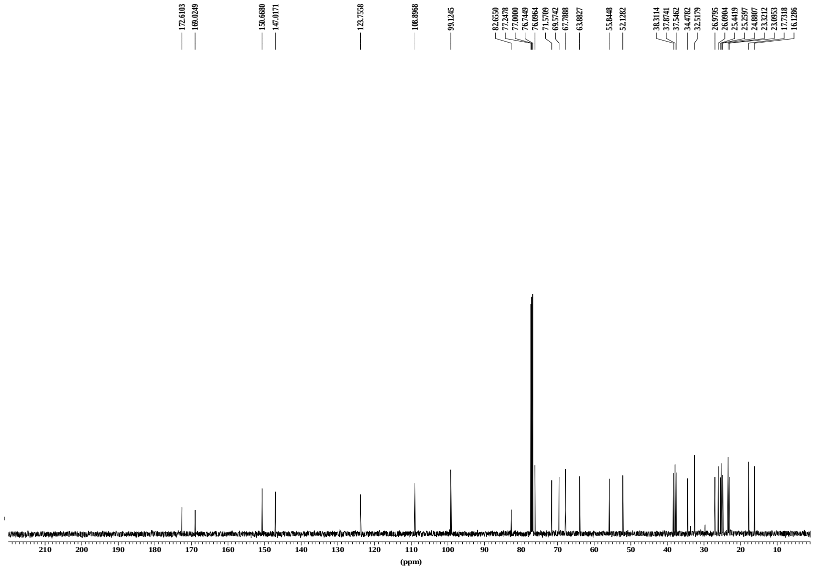

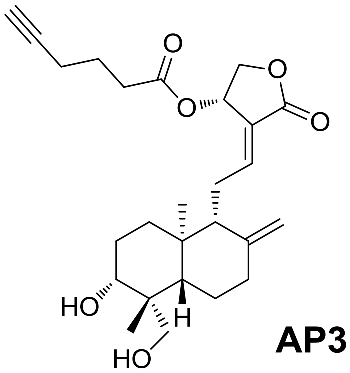

AP3

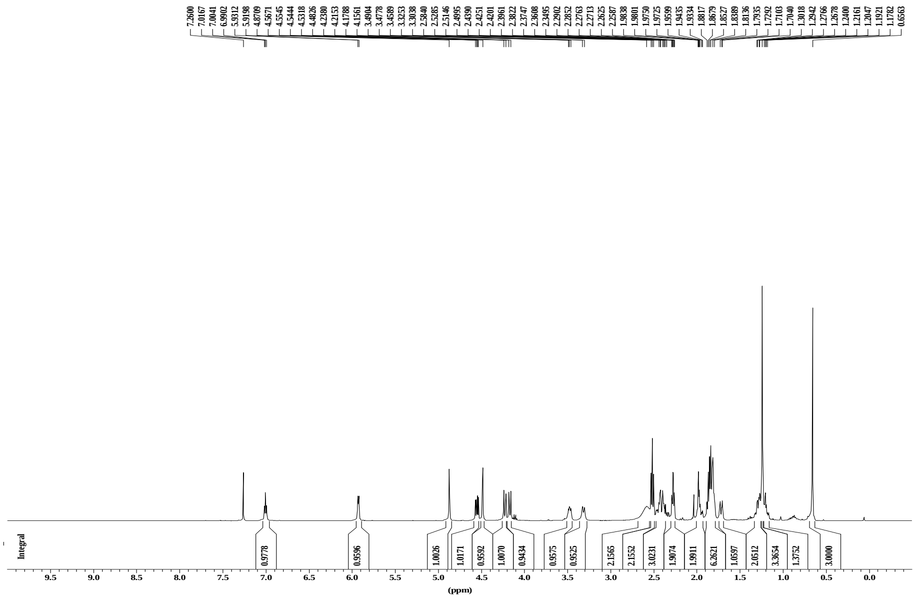

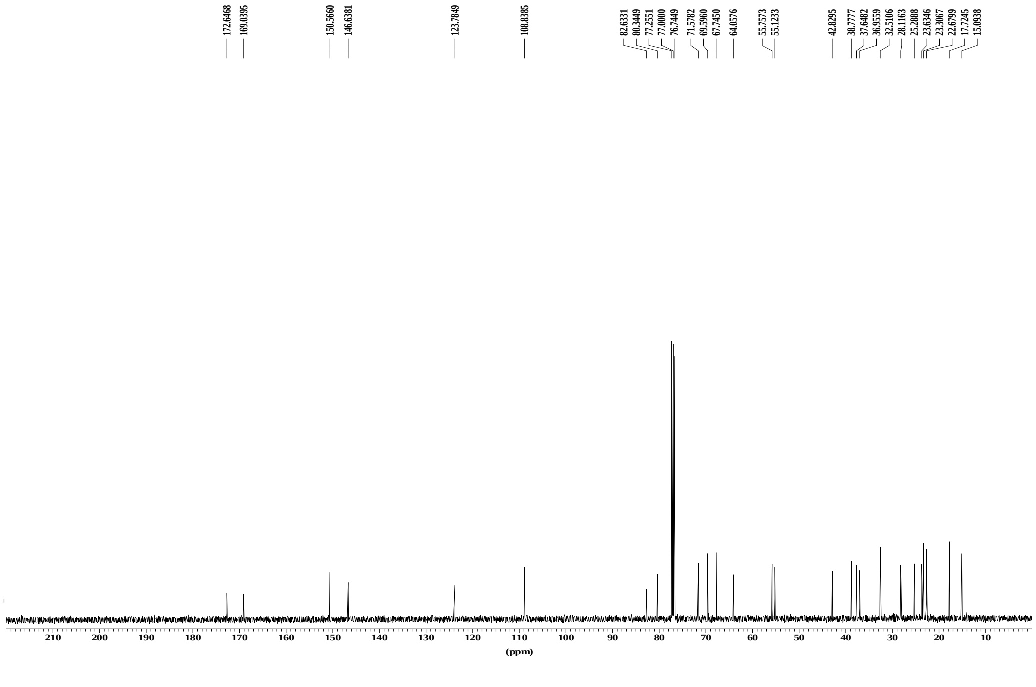

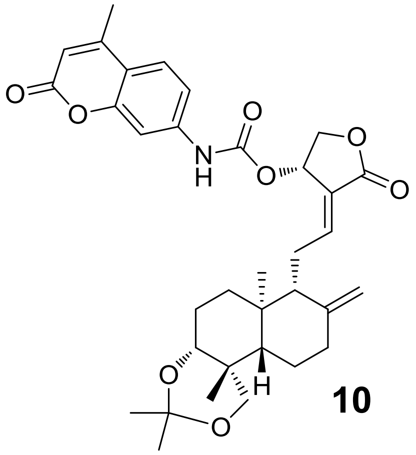

10

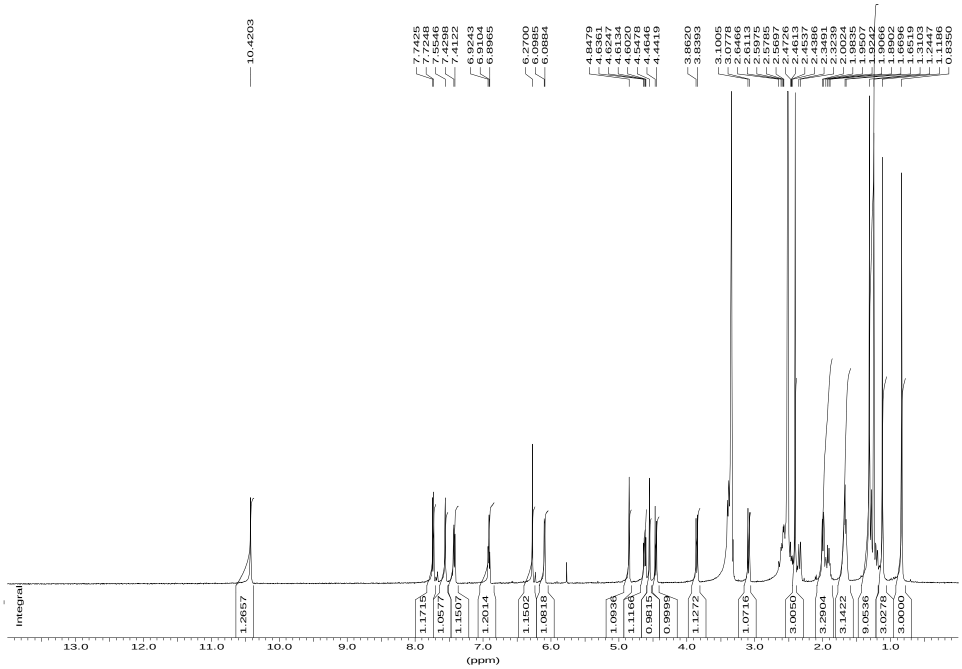

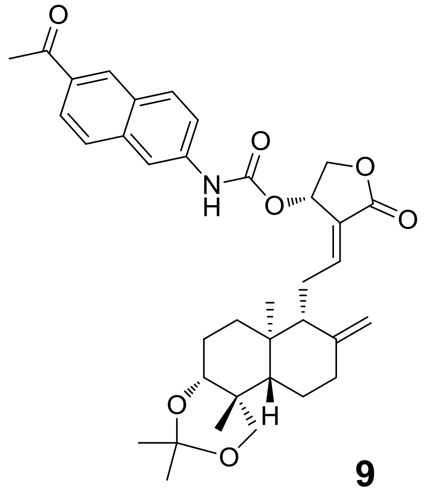
O
NH
O
O
O
O
O
H
O
O

9

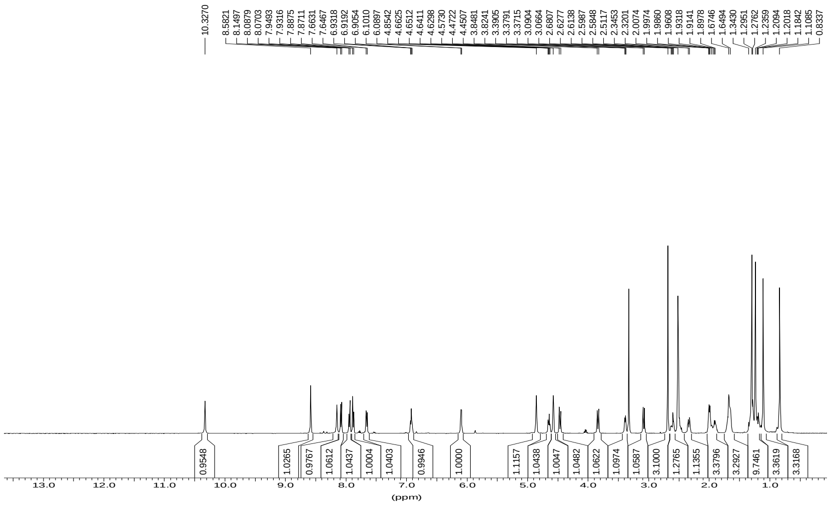
10.3270
8.5821 8.1497 8.0879 8.0703 7.9493 7.9316 7.8875 7.8711 7.6631 7.6467 6.9318 6.9192 6.9054 6.1010 6.0897 4.8542 4.6625 4.6512 4.6411 4.6298 4.5730 4.4722 4.4507 3.8481 3.8241 3.3905 3.3791 3.3715 3.0904 3.0664 2.6807 2.6277 2.6138 2.5987 2.5848 2.5117 2.3453 2.3201 2.0074 1.9974 1.9860 1.9608 1.9318 1.9141 1.8978 1.6746 1.6494 1.3430 1.2951 1.2762 1.2359 1.2094 1.2018 1.1842 1.1085 0.8337
0.9548 1.0265 0.9767 1.0612 1.0437 1.0004 1.0403 0.9946 1.0000 1.1157 1.0438 1.0047 1.0482 1.0622 1.0974 1.0587 3.1000 1.2765 1.1355 3.3796 3.2927 9.7461 3.3619 3.3168
13.0 12.0 11.0 10.0 9.0 8.0 7.0 6.0 5.0 4.0 3.0 2.0 1.0
(ppm)

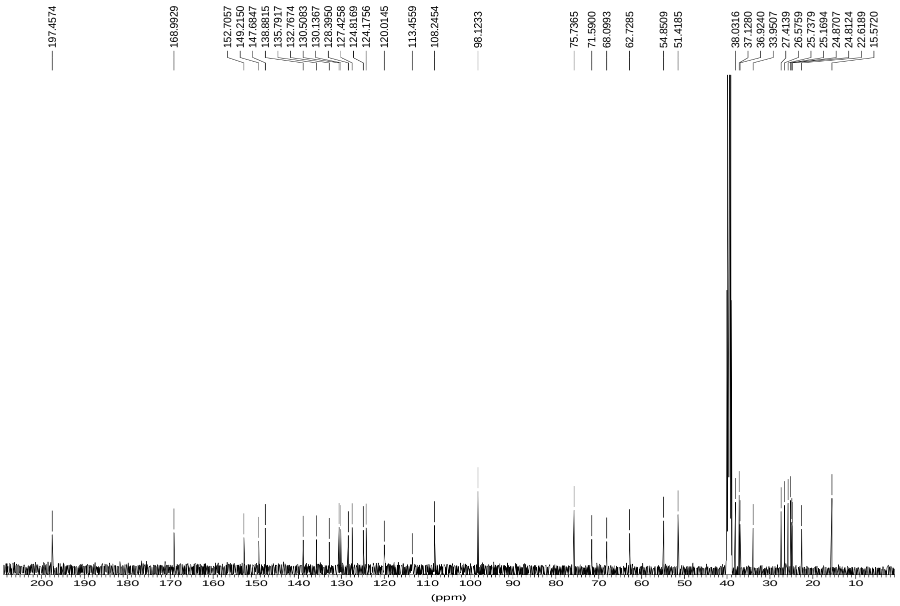
197.4574
168.9929
152.7057 149.2150 147.6847 138.8815 135.7917 132.7674 130.5083 130.1367 128.3950 127.4258 124.8169 124.1756
120.0145
113.4559
108.2454
98.1233
75.7365
71.5900
68.0993
62.7285
54.8509
51.4185
38.0316 37.1280 36.9240 33.9507 27.4139 26.5759 25.7379 25.1694 24.8707 24.8124 22.6189 15.5720
200 190 180 170 160 150 140 130 120 110 100 90 80 70 60 50 40 30 20 10
(ppm)

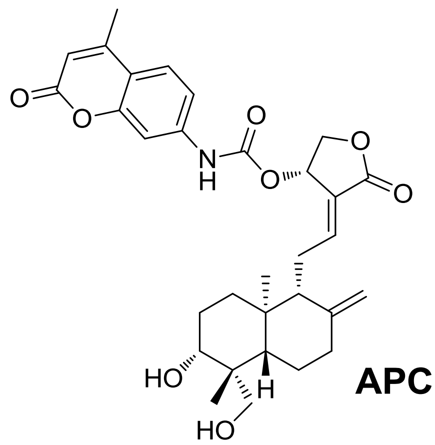

APCM

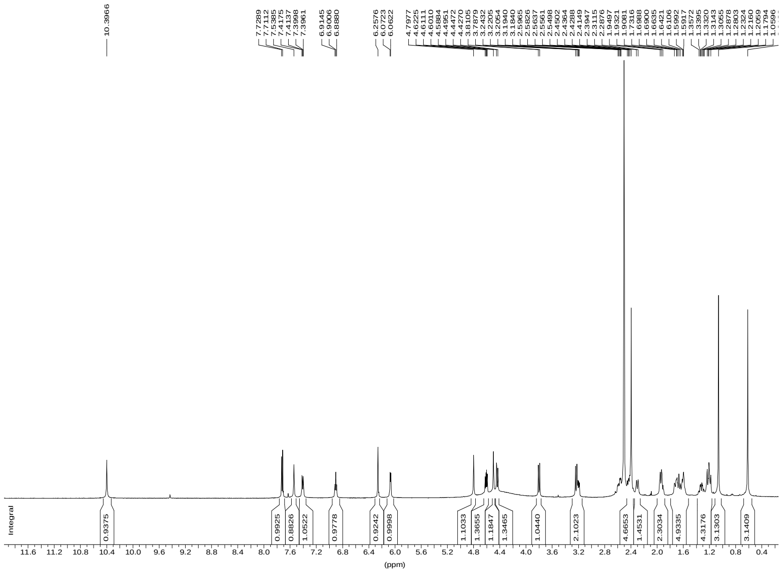

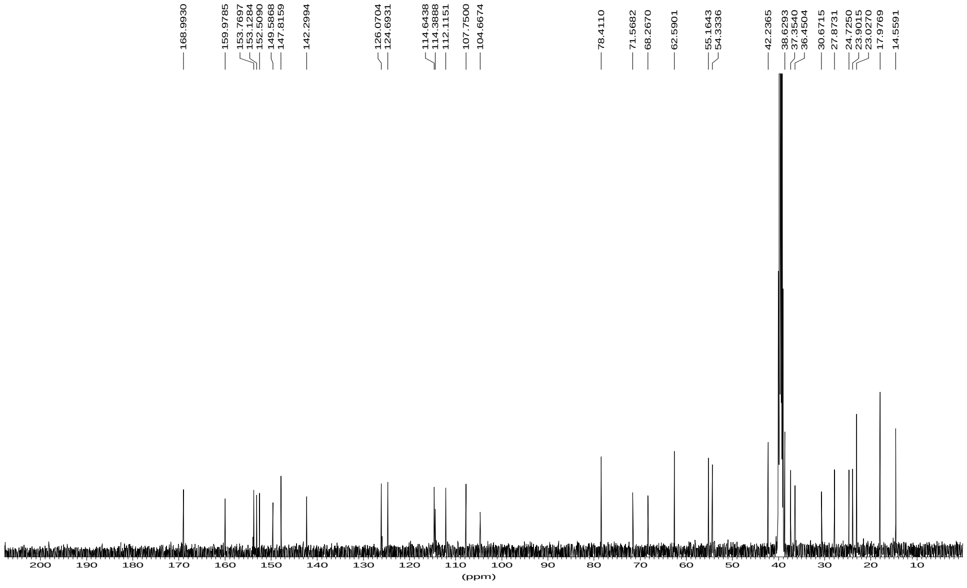

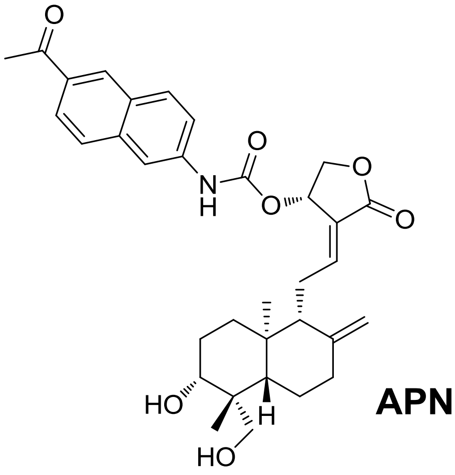
O
N
H
O
O
O
O
HO
H
HO

APNP

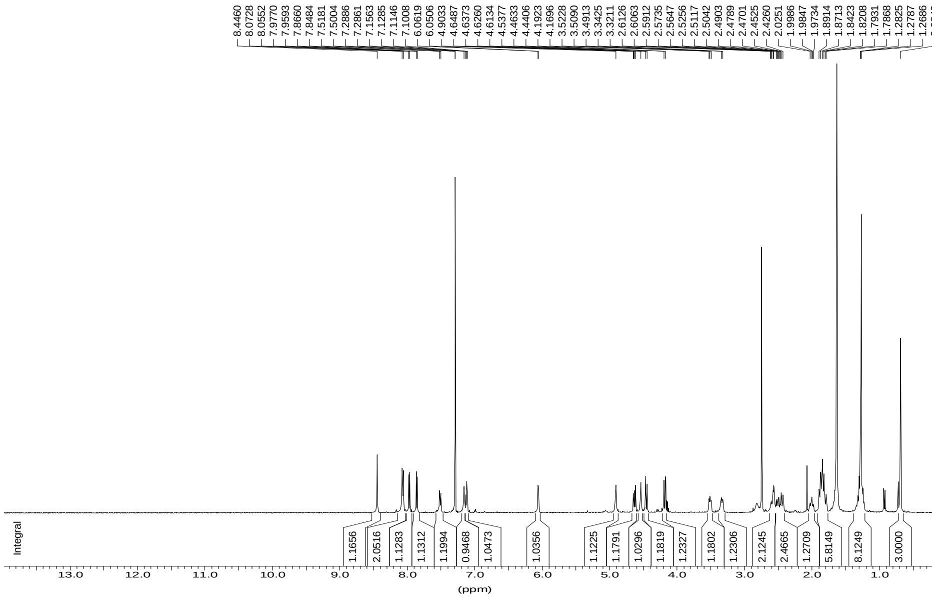
Integral
(ppm)
1.1656
2.0516
1.1283
1.1312
1.1994
0.9468
1.0473
1.0356
1.1225
1.1791
1.0296
1.1819
1.2327
1.1802
1.2306
2.1245
2.4665
1.2709
5.8149
8.1249
3.0000

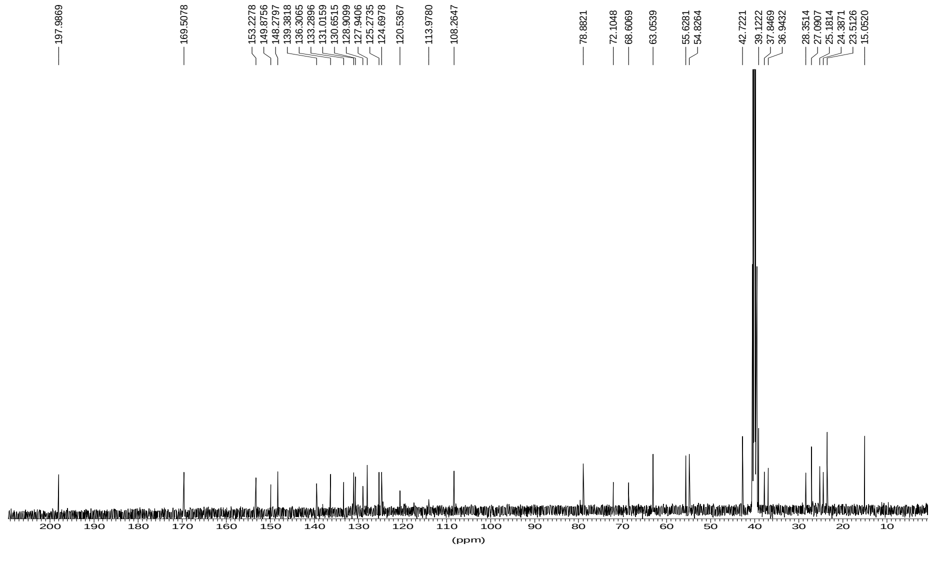
197.9869
169.5078
153.2278
149.8756
148.2797
139.3818
136.3065
133.2896
131.0159
130.6515
128.9099
127.9406
125.2735
124.6978
120.5367
113.9780
108.2647
78.8821
72.1048
68.6069
63.0539
55.6281
54.8264
42.7221
39.1222
37.8469
36.9432
28.3514
27.0907
25.1814
24.3871
23.5126
15.0520
(ppm)

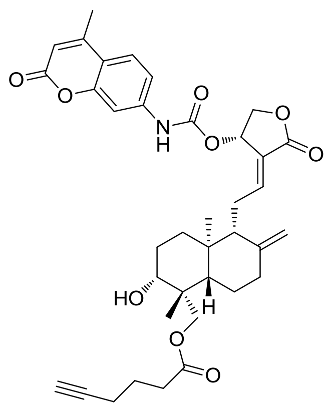
O
O
H
N
O
O
O
O
HO
H
O
O

AP1CM

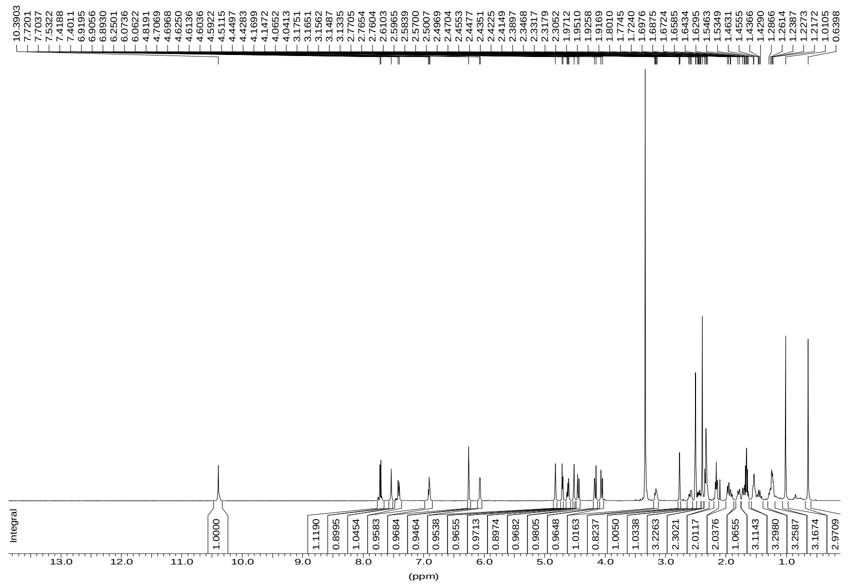
10.3903
7.7201
7.7037
7.5322
7.4188
7.4011
6.9195
6.9056
6.8930
6.2501
6.0736
6.0622
4.8191
4.7069
4.6968
4.6250
4.6136
4.6036
4.5922
4.5115
4.4497
4.4283
4.1699
4.1472
4.0652
4.0413
3.1751
3.1651
3.1562
3.1487
3.1325
2.7705
2.7654
2.7604
2.6103
2.5965
2.5839
2.5700
2.5007
2.4969
2.4704
2.4553
2.4477
2.4351
2.4225
2.4149
2.3897
2.3468
2.3317
2.3179
2.3052
1.9712
1.9510
1.9258
1.9169
1.8010
1.7745
1.7240
1.6976
1.6875
1.6724
1.6585
1.6434
1.6295
1.5463
1.5349
1.4631
1.4555
1.4366
1.4290
1.2866
1.2614
1.2387
1.2273
1.2172
1.0105
0.6398
Integral
1.0000
1.1190
0.8995
1.0454
0.9583
0.9684
0.9464
0.9538
0.9655
0.9713
0.8974
0.9682
0.9805
0.9648
1.0163
0.8237
1.0050
1.0338
3.2263
2.3021
2.0117
2.0376
1.0655
3.1143
3.2980
3.2587
3.1674
2.9709
13.0
12.0
11.0
10.0
9.0
8.0
7.0
6.0
5.0
4.0
3.0
2.0
1.0
(ppm)

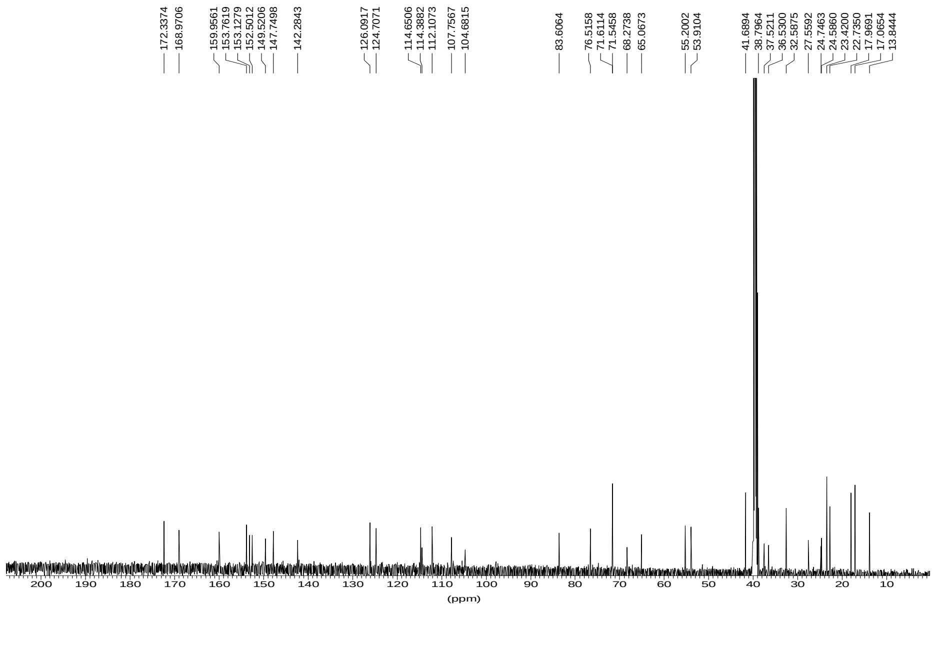
172.3374
168.9706
159.9561
153.7619
153.1279
152.5012
149.5206
147.7498
142.2843
126.0917
124.7071
114.6506
114.3882
112.1073
107.7567
104.6815
83.6064
76.5158
71.6114
71.5458
68.2738
65.0673
55.2002
53.9104
41.6894
38.7964
37.5211
36.5300
32.5875
27.5592
24.7463
24.5860
23.4200
22.7350
17.9691
17.0654
13.8444
200
190
180
170
160
150
140
130
120
110
100
90
80
70
60
50
40
30
20
10
(ppm)

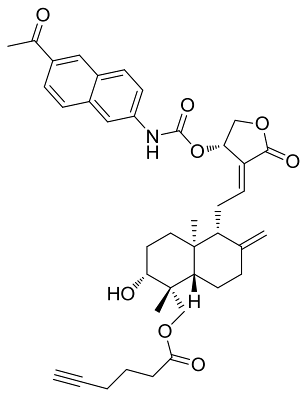

AP1NP

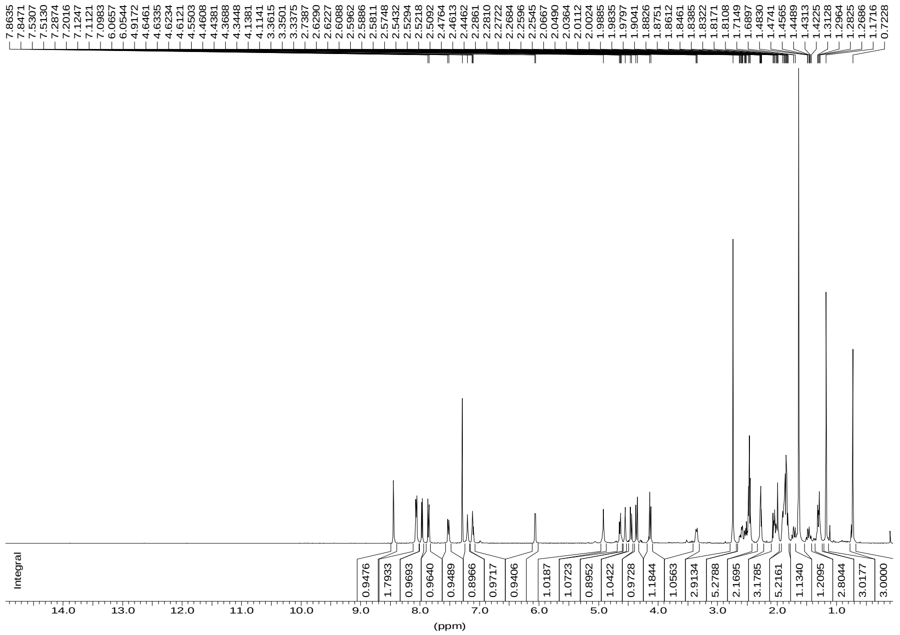

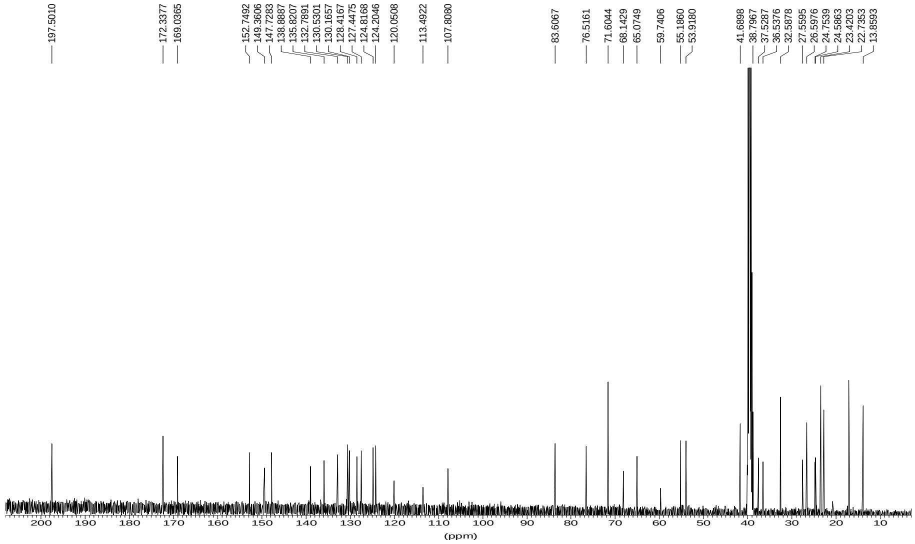

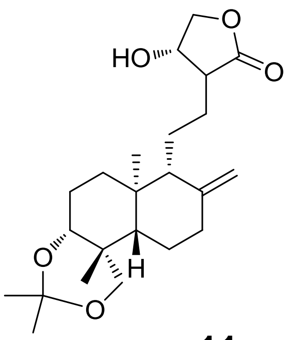
HO
O
O
O
H
O

11

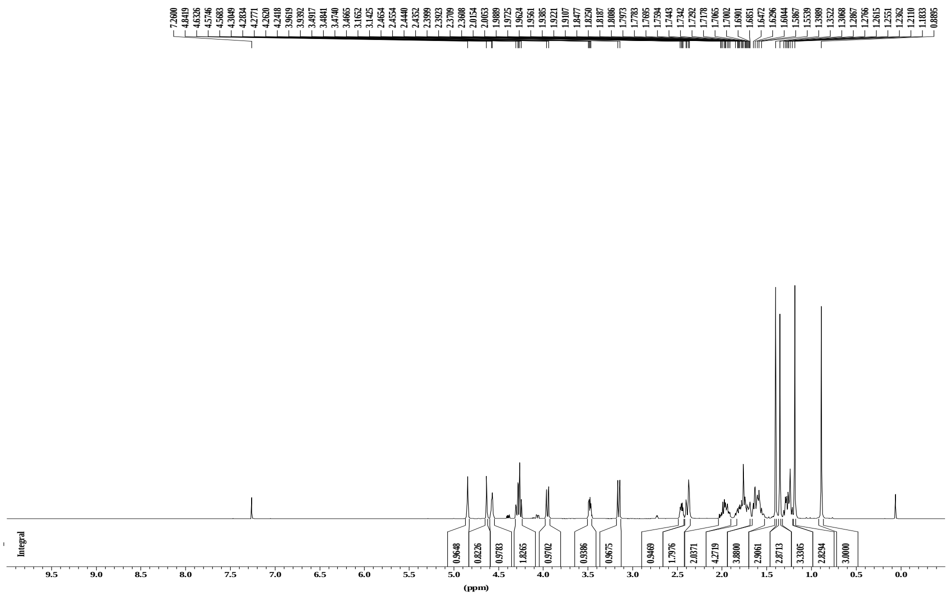
7.2600 4.8419 4.6326 4.5746 4.5683 4.3049 4.2834 4.2771 4.2620 4.2418 3.9619 3.9392 3.4917 3.4841 3.4740 3.4665 3.1652 3.1425 2.4654 2.4554 2.4440 2.4352 2.3999 2.3923 2.3709 2.3608 2.0154 2.0053 1.9889 1.9725 1.9624 1.9561 1.9385 1.9221 1.9107 1.8477 1.8250 1.8187 1.8086 1.7973 1.7783 1.7695 1.7594 1.7443 1.7342 1.7292 1.7178 1.7065 1.7002 1.6901 1.6851 1.6472 1.6296 1.6044 1.5867 1.5539 1.3989 1.3522 1.3068 1.2867 1.2766 1.2615 1.2551 1.2362 1.2110 1.1833 0.8895
Integral
0.9648 0.8226 0.9783 1.8265 0.9702 0.9386 0.9675 0.9469 1.7976 2.0371 4.2719 3.8800 2.9061 2.8713 3.3305 2.8294 3.0000
9.5 9.0 8.5 8.0 7.5 7.0 6.5 6.0 5.5 5.0 4.5 4.0 3.5 3.0 2.5 2.0 1.5 1.0 0.5 0.0
(ppm)

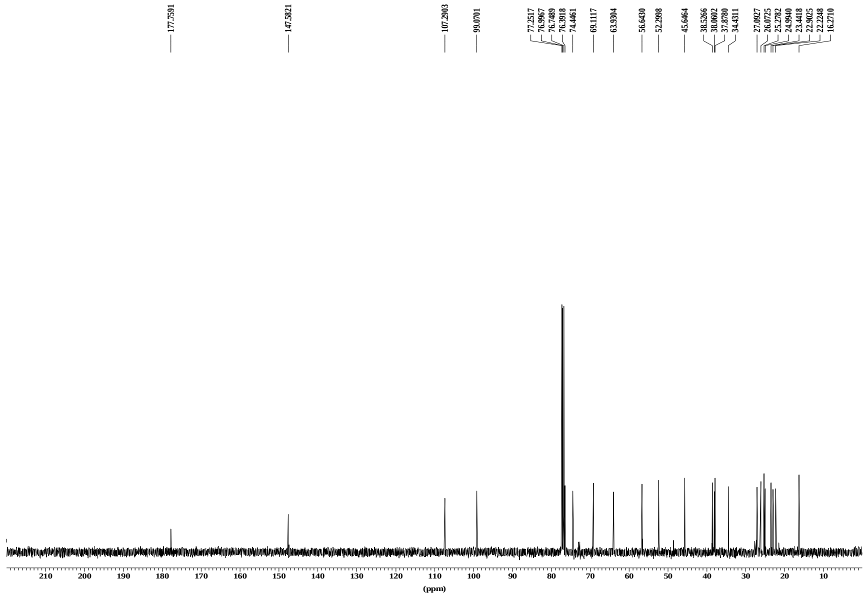
177.7591 147.5821 107.2903 99.0701 77.2517 76.9967 76.7489 76.3918 74.4461 69.1117 63.9304 56.6430 52.2998 45.6464 38.5266 38.0602 37.8780 34.4311 27.0927 26.0725 25.2782 24.9940 23.4418 22.9025 22.2248 16.2710
210 200 190 180 170 160 150 140 130 120 110 100 90 80 70 60 50 40 30 20 10
(ppm)

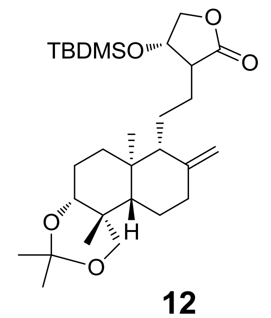

TBDMSO
O
O
O
O
H

12

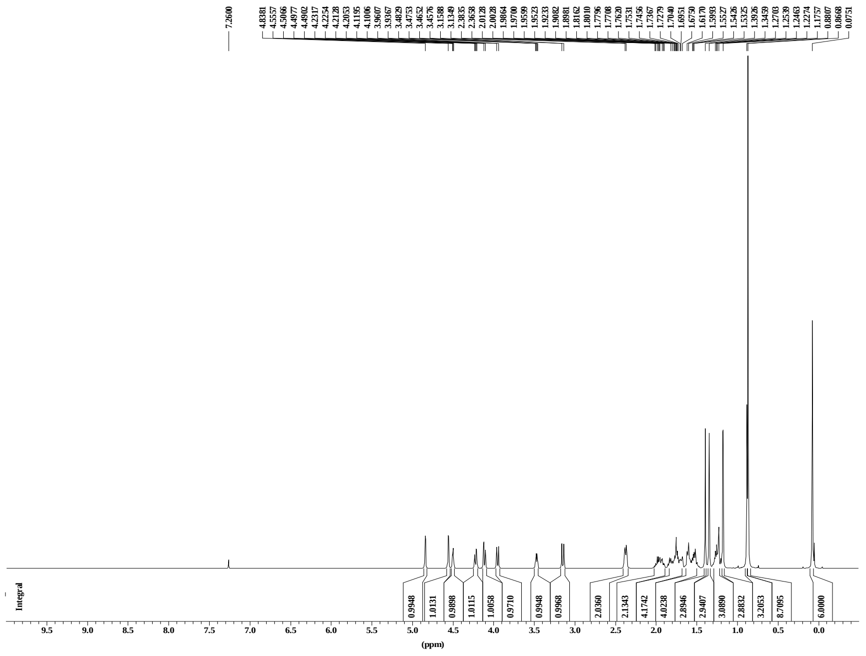

7.2600
4.8381 4.5557 4.5066 4.4977 4.4902 4.2317 4.2254 4.2128 4.2053 4.1195 4.1006 3.9607 3.9367 3.4829 3.4753 3.4652 3.4576 3.1588 3.1349 2.3835 2.3658 2.0128 2.0028 1.9864 1.9700 1.9599 1.9523 1.9233 1.9082 1.8981 1.8162 1.8010 1.7796 1.7708 1.7620 1.7531 1.7456 1.7367 1.7279 1.7040 1.6951 1.6750 1.6170 1.5993 1.5527 1.5426 1.5325 1.3926 1.3439 1.2703 1.2539 1.2463 1.2274 1.1757 0.8807 0.8668 0.0751
Integral
0.9948 1.0131 0.9898 1.0115 1.0058 0.9710 0.9948 0.9968 2.0360 2.1343 4.1742 4.0238 2.8946 2.9407 3.0890 2.8832 3.2053 8.7095 6.0000
9.5 9.0 8.5 8.0 7.5 7.0 6.5 6.0 5.5 5.0 4.5 4.0 3.5 3.0 2.5 2.0 1.5 1.0 0.5 0.0
(ppm)

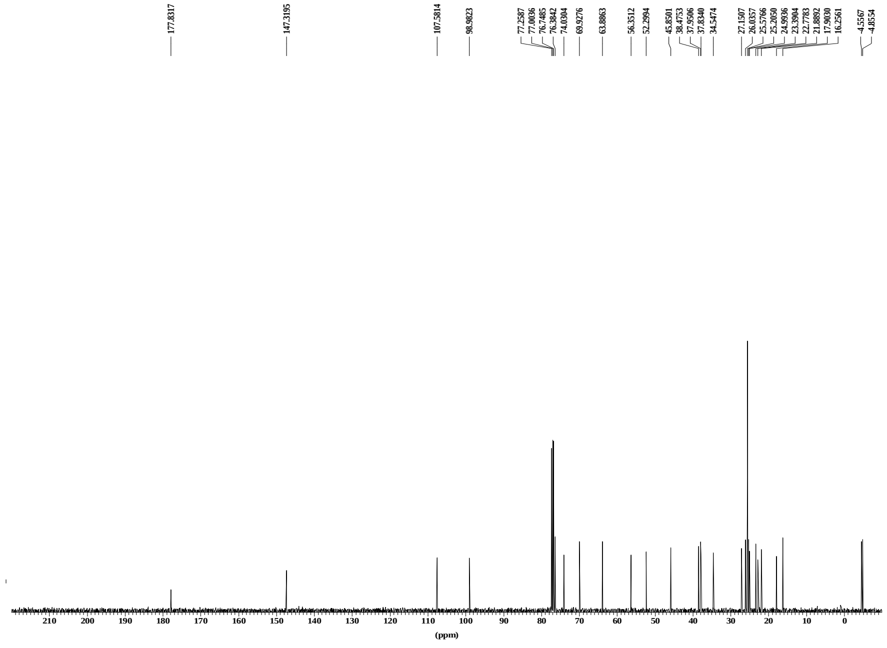

177.8317
147.3195
107.5814 98.9823
77.2587 77.0036 76.7485 76.3842 74.0304 69.9276 63.8863 56.3512 52.2994 45.8501 38.4753 37.9506 37.8340 34.5474 27.1507 26.0357 25.5766 25.2050 24.9936 23.3904 22.7783 21.8892 17.9030 16.2561 -4.5567 -4.8554
210 200 190 180 170 160 150 140 130 120 110 100 90 80 70 60 50 40 30 20 10 0
(ppm)

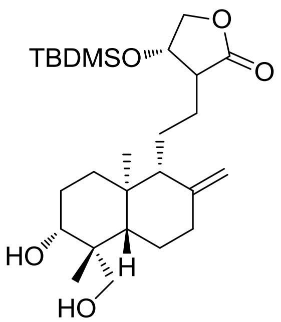

13

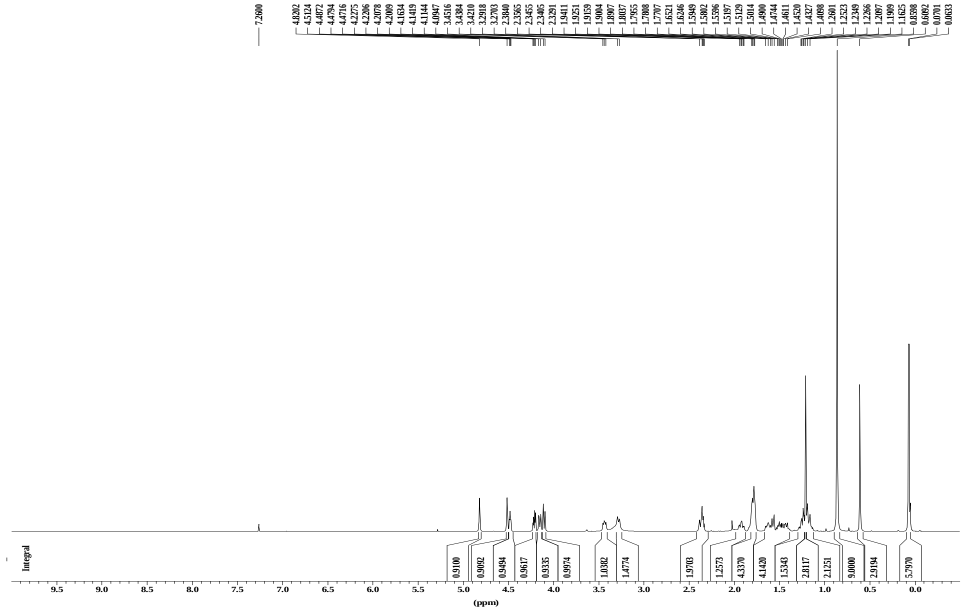

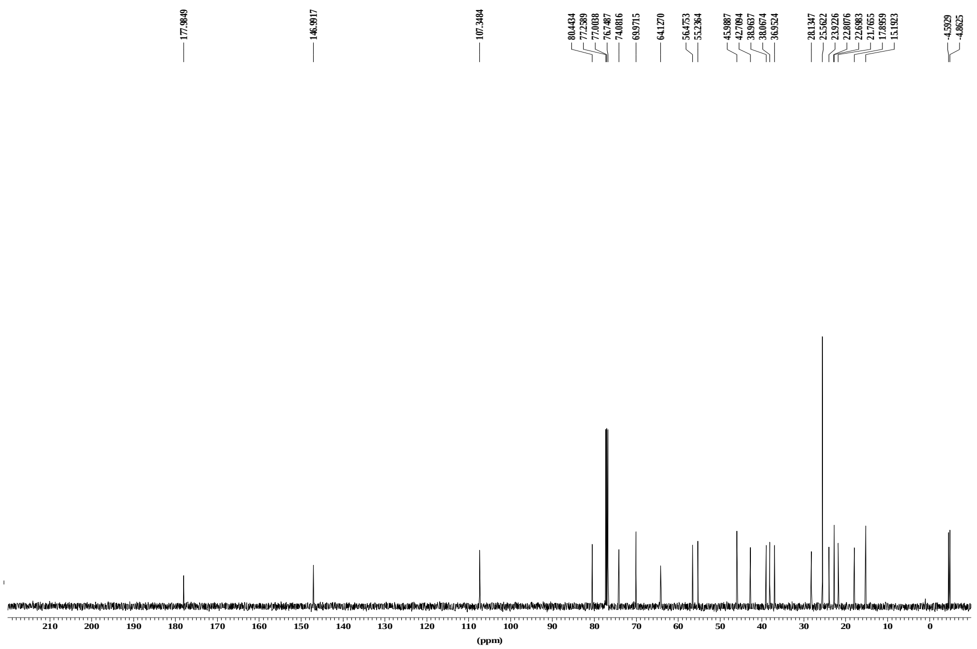

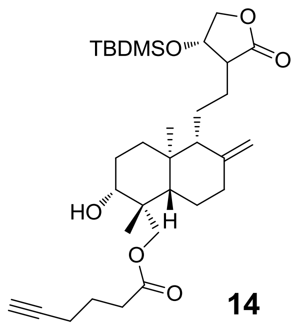

14

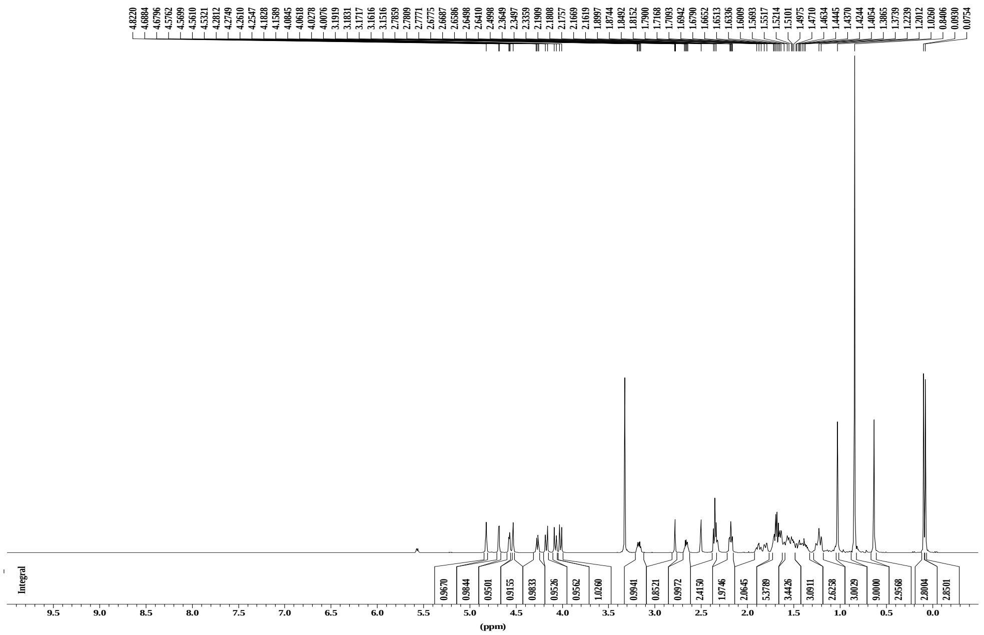

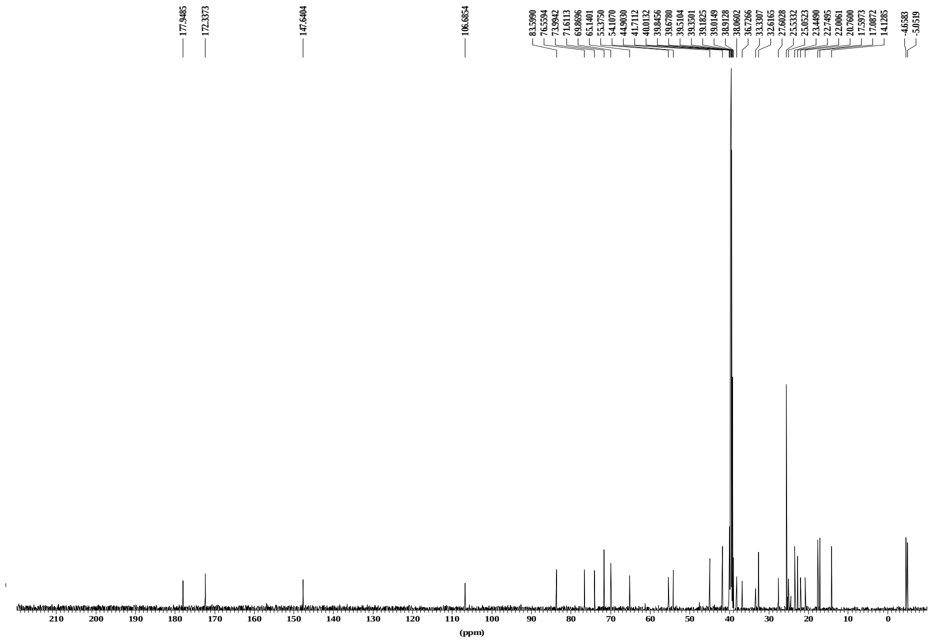

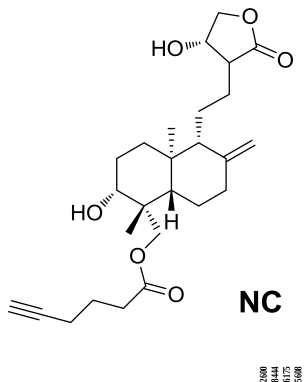
HO
O
O
HO
H
O
O

NC

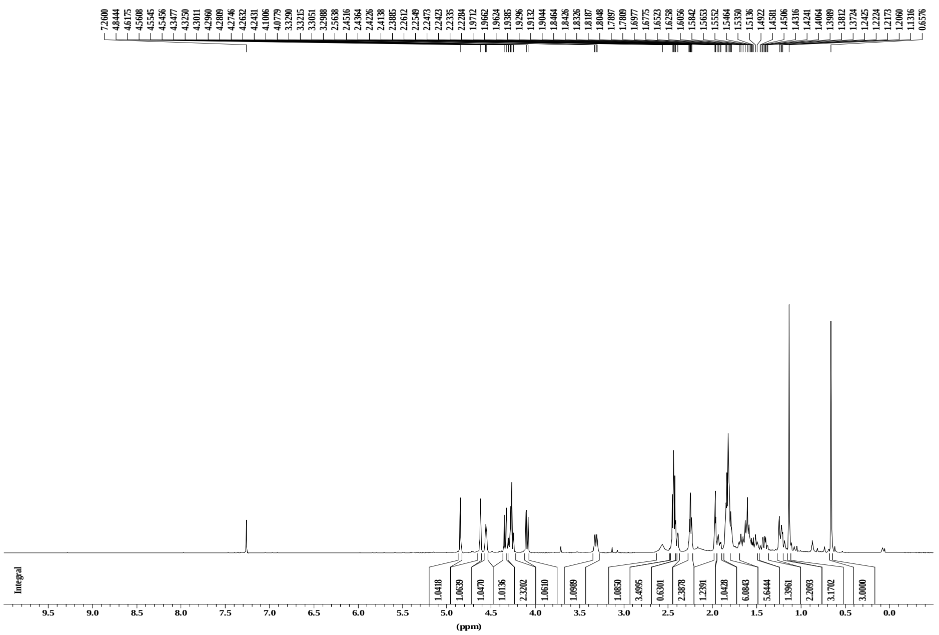
Integral
1.0418
1.0639
1.0470
1.0136
2.3202
1.0610
1.0989
1.0850
3.4995
0.6301
2.3878
1.2391
1.0428
6.0843
5.6444
1.3961
2.2093
3.1702
3.0000
9.5 9.0 8.5 8.0 7.5 7.0 6.5 6.0 5.5 5.0 4.5 4.0 3.5 3.0 2.5 2.0 1.5 1.0 0.5 0.0
(ppm)

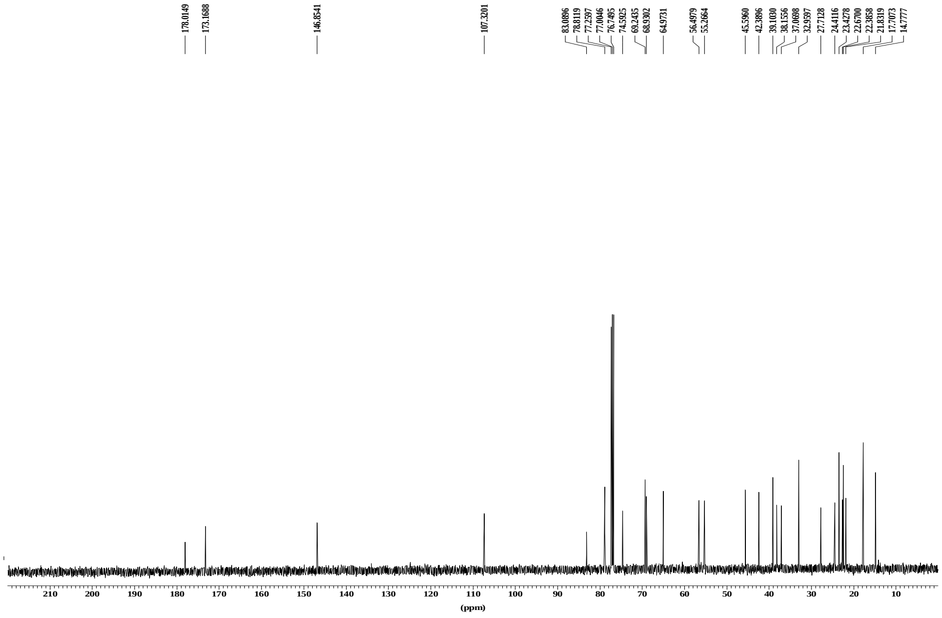
178.0149
173.1688
146.8541
107.3201
83.0896
78.8119
77.2597
77.0046
76.7495
74.5925
69.2435
68.9302
64.9731
56.4979
55.2664
45.5960
42.3896
39.1030
38.1556
37.0698
32.9597
27.7128
24.4116
23.4278
22.6700
22.3858
21.8319
17.7073
14.7777
210 200 190 180 170 160 150 140 130 120 110 100 90 80 70 60 50 40 30 20 10
(ppm)

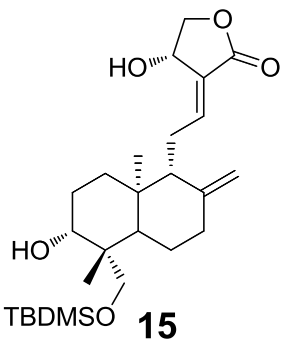

15

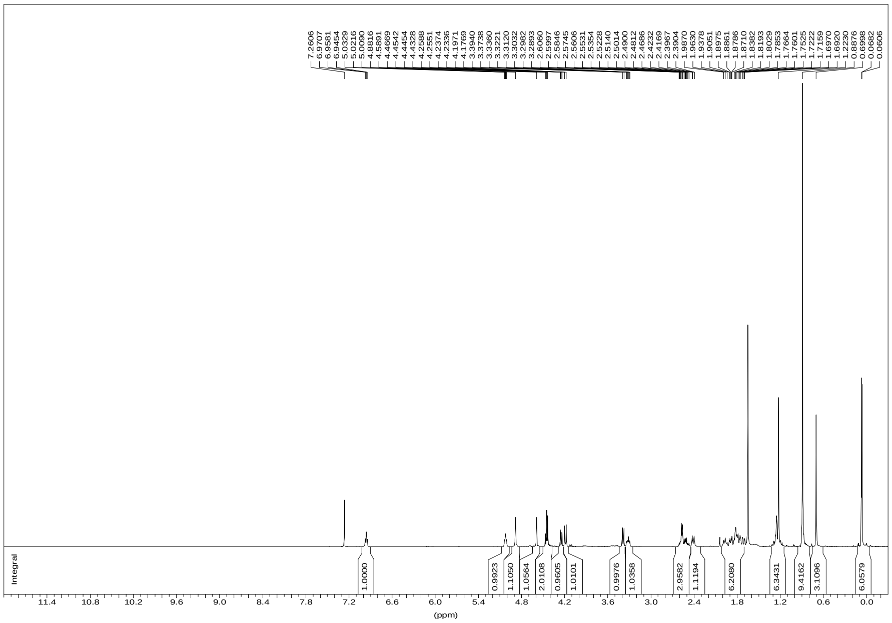

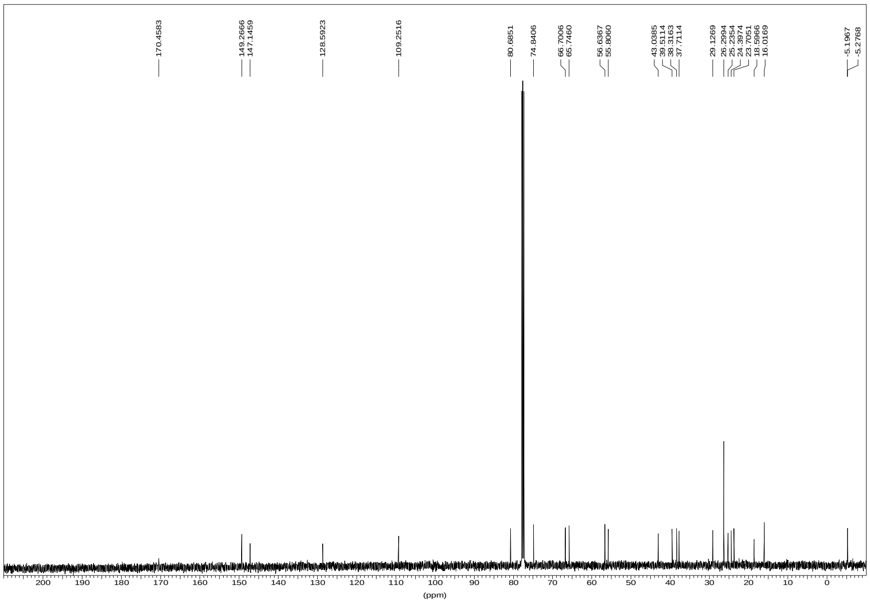

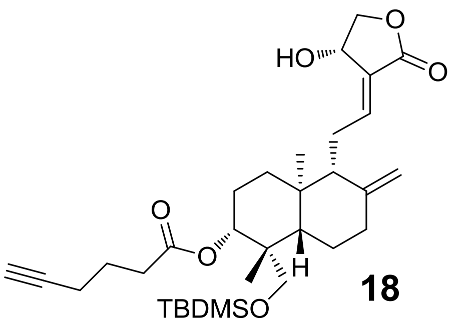
HO
O
O
O
O
H
TBDMSO

18

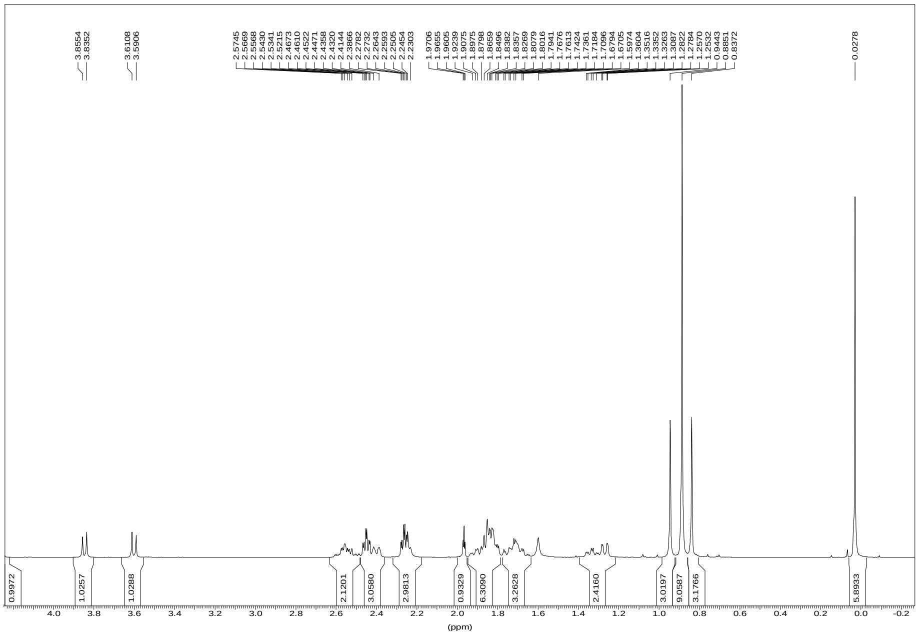
0.9972
1.0257
1.0288
2.1201
3.0580
2.9813
0.9329
6.3090
3.2628
2.4160
3.0197
9.0587
3.1766
5.8933
(ppm)

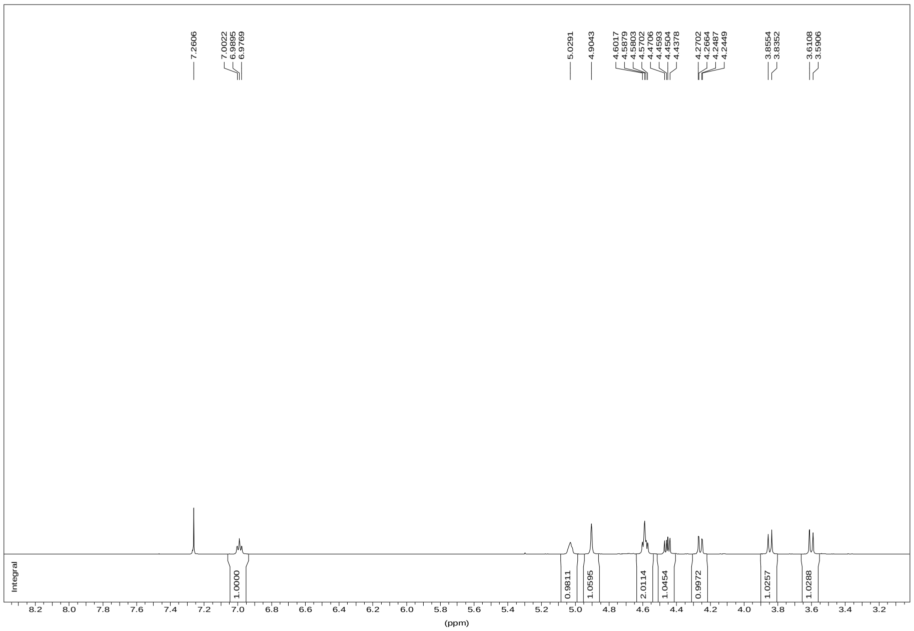
Integral
1.0000
0.9811
1.0595
2.0114
1.0454
0.9972
1.0257
1.0288
(ppm)

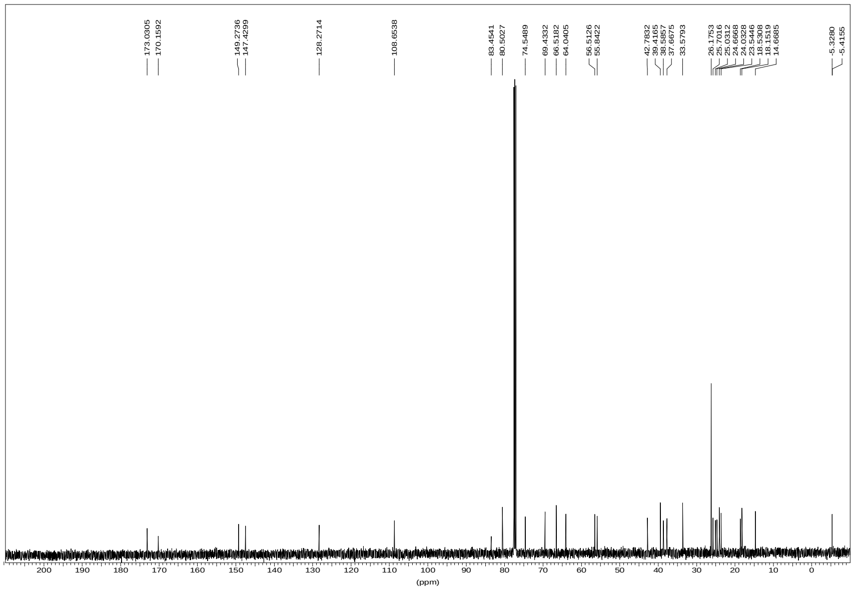

173.0305
170.1592
149.2736
147.4299
128.2714
108.6538
83.4541
80.5027
74.5489
69.4332
66.5182
64.0405
56.5126
55.8422
42.7832
39.4165
38.5857
37.6675
33.5793
26.1753
25.7016
25.0312
24.6668
24.0328
23.5446
18.5308
18.1519
14.6685
-5.3280
-5.4155
200 190 180 170 160 150 140 130 120 110 100 90 80 70 60 50 40 30 20 10 0
(ppm)

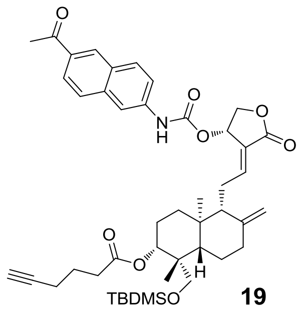

O
NH
O
O
O
O
O
O
H
TBDMSO

19

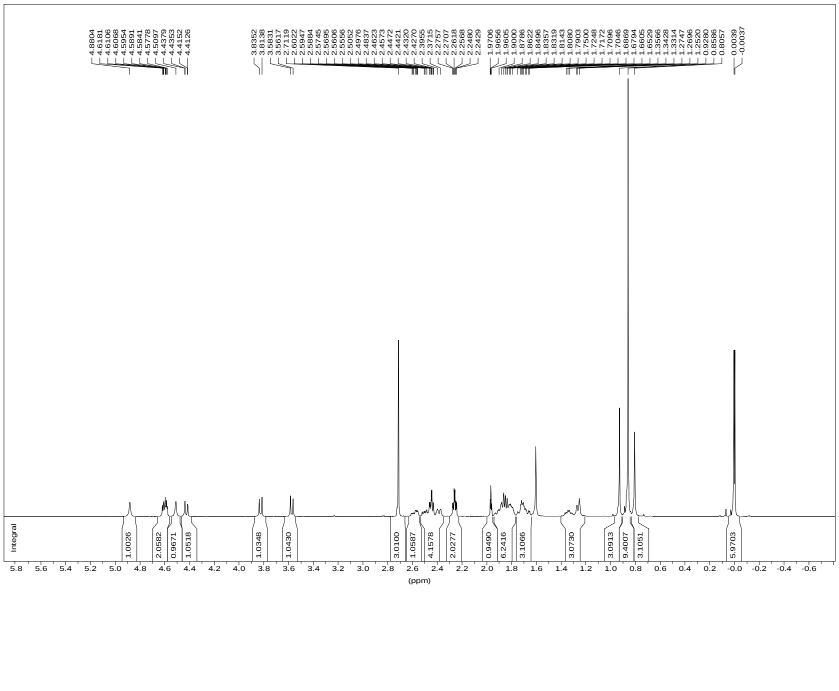

4.8804
4.6181
4.6106
4.6068
4.5954
4.5891
4.5841
4.5778
4.5097
4.4379
4.4353
4.4152
4.4126
3.8352
3.8138
3.5831
3.5617
2.7119
2.6022
2.5947
2.5884
2.5745
2.5695
2.5606
2.5556
2.5052
2.4976
2.4837
2.4623
2.4573
2.4472
2.4421
2.4320
2.4270
2.3955
2.3715
2.2757
2.2707
2.2618
2.2568
2.2480
2.2429
1.9706
1.9656
1.9605
1.9000
1.8786
1.8622
1.8496
1.8357
1.8319
1.8143
1.8080
1.7903
1.7500
1.7248
1.7172
1.7096
1.7046
1.6869
1.6794
1.6605
1.6529
1.3566
1.3428
1.3314
1.2747
1.2696
1.2520
0.9280
0.8586
0.8057
0.0039
-0.0037
Integral
1.0026
2.0582
0.9671
1.0518
1.0348
1.0430
3.0100
1.0587
4.1578
2.0277
0.9490
6.2416
3.1066
3.0730
3.0913
9.4007
3.1051
5.9703
5.8 5.6 5.4 5.2 5.0 4.8 4.6 4.4 4.2 4.0 3.8 3.6 3.4 3.2 3.0 2.8 2.6 2.4 2.2 2.0 1.8 1.6 1.4 1.2 1.0 0.8 0.6 0.4 0.2 -0.0 -0.2 -0.4 -0.6
(ppm)

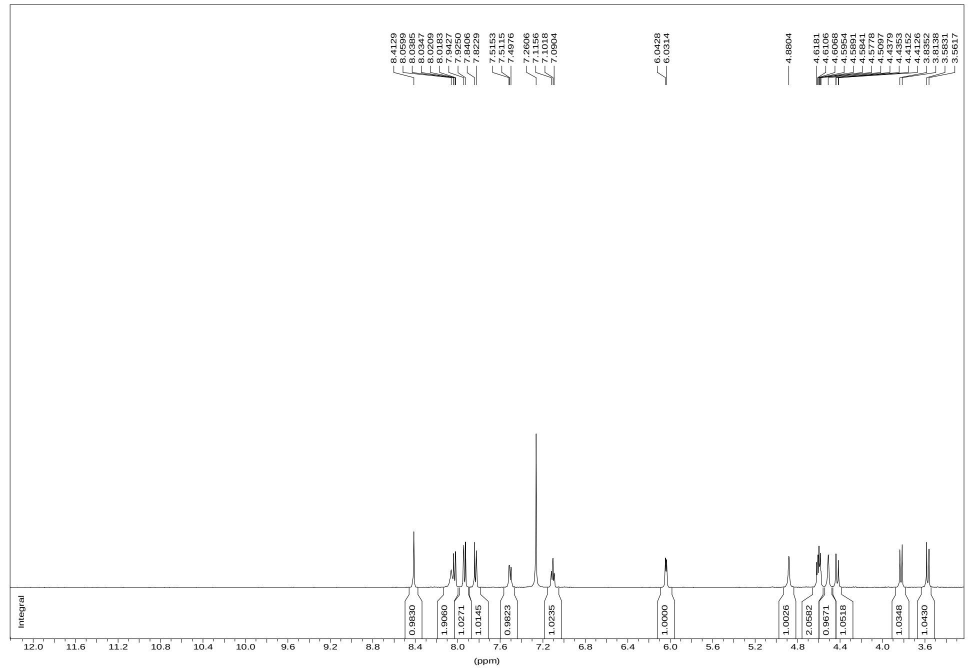

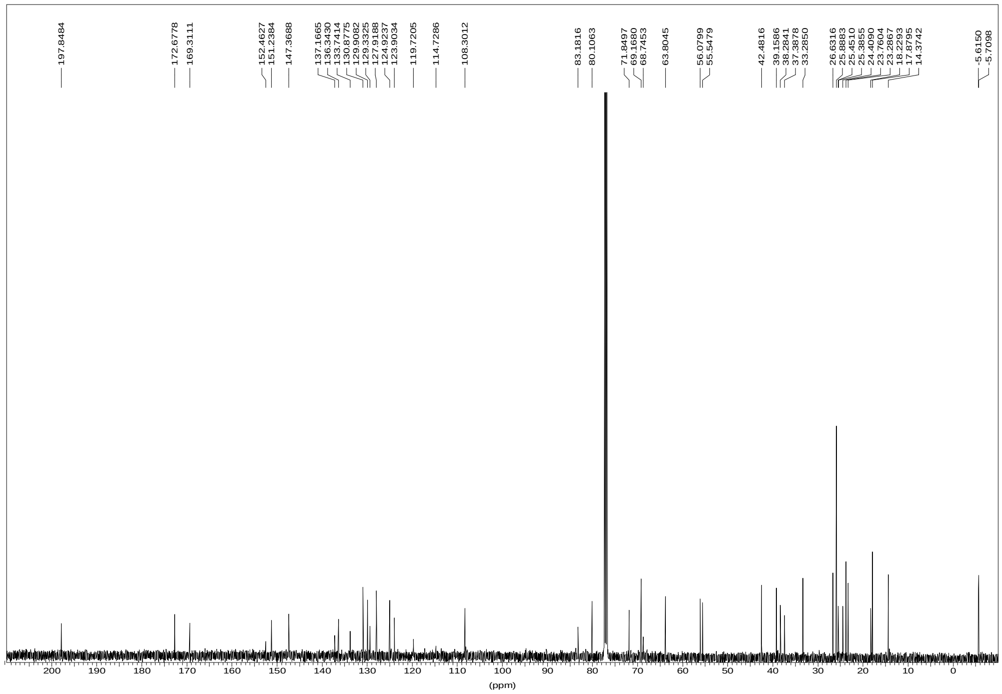

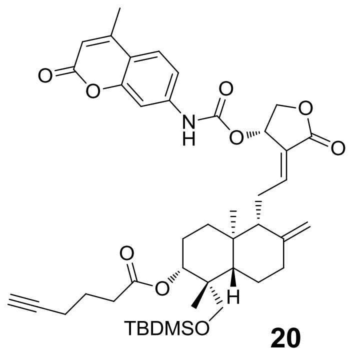

20

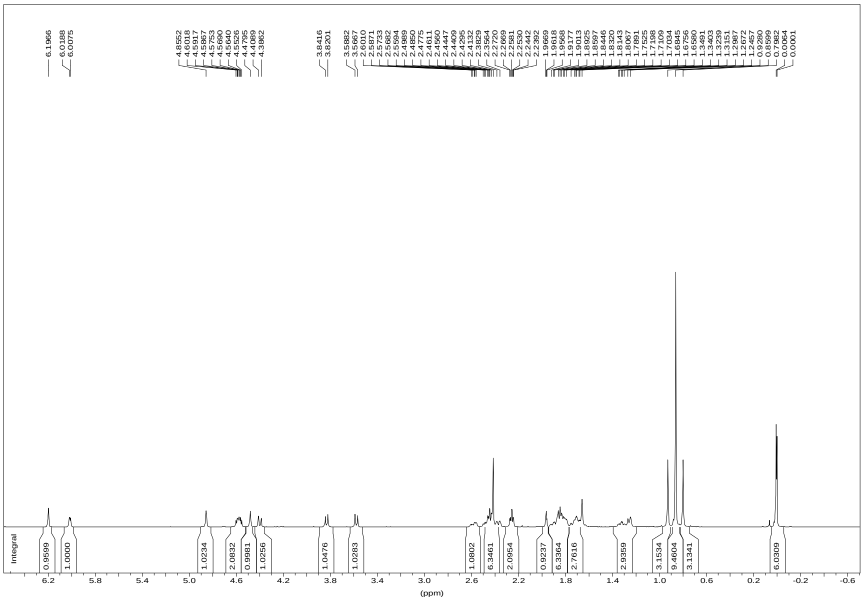

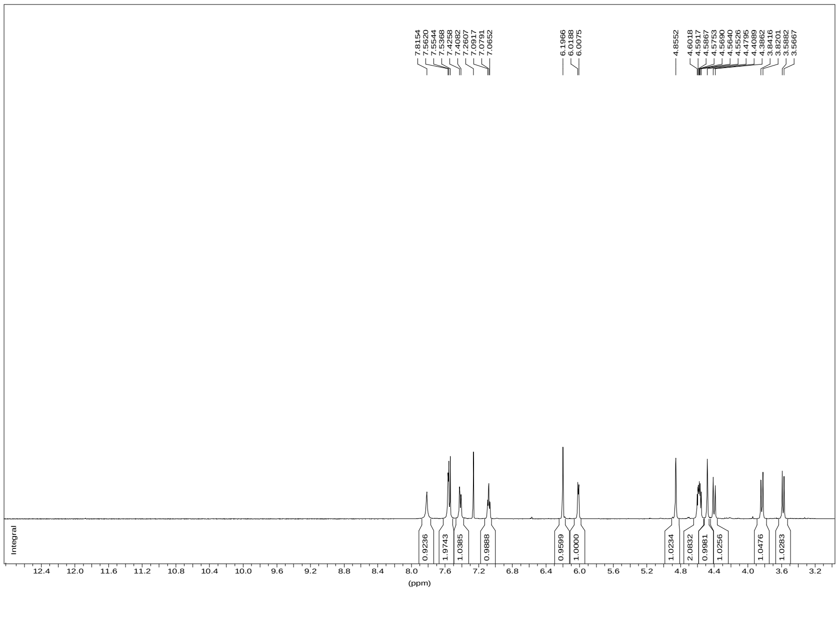

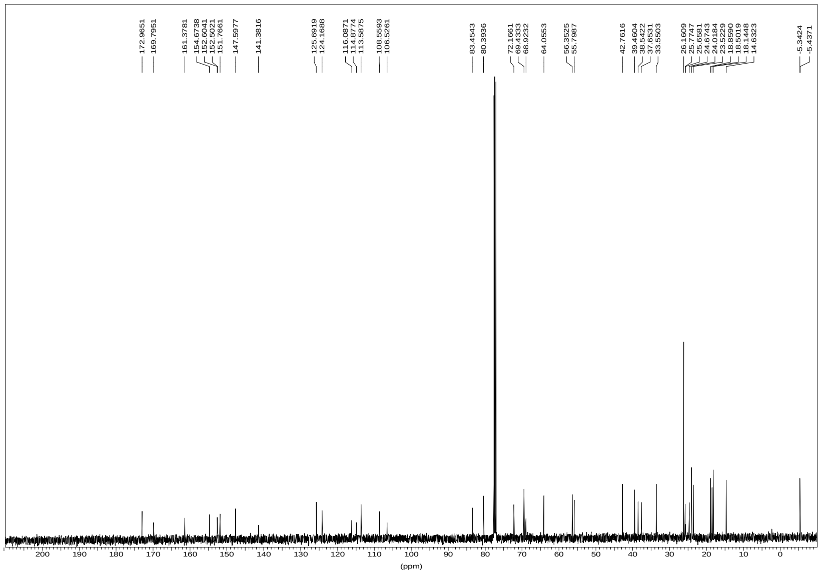

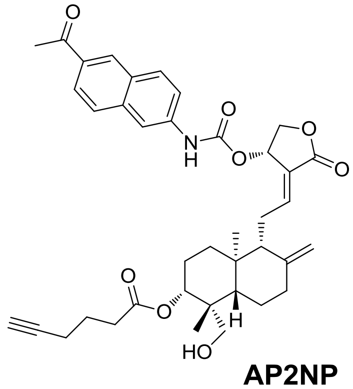

AP2NP

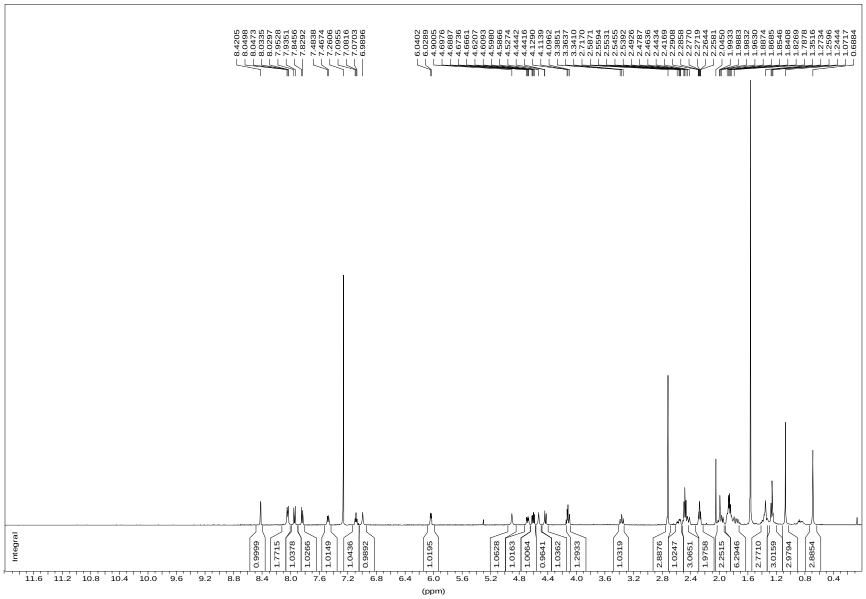

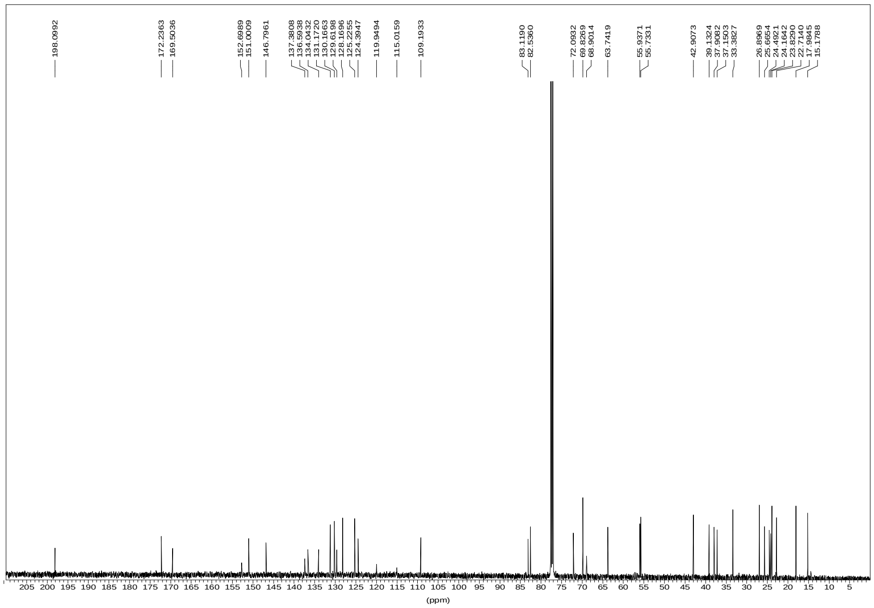

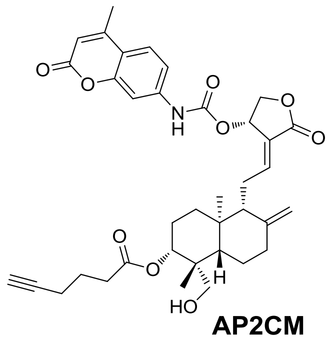

AP2CM

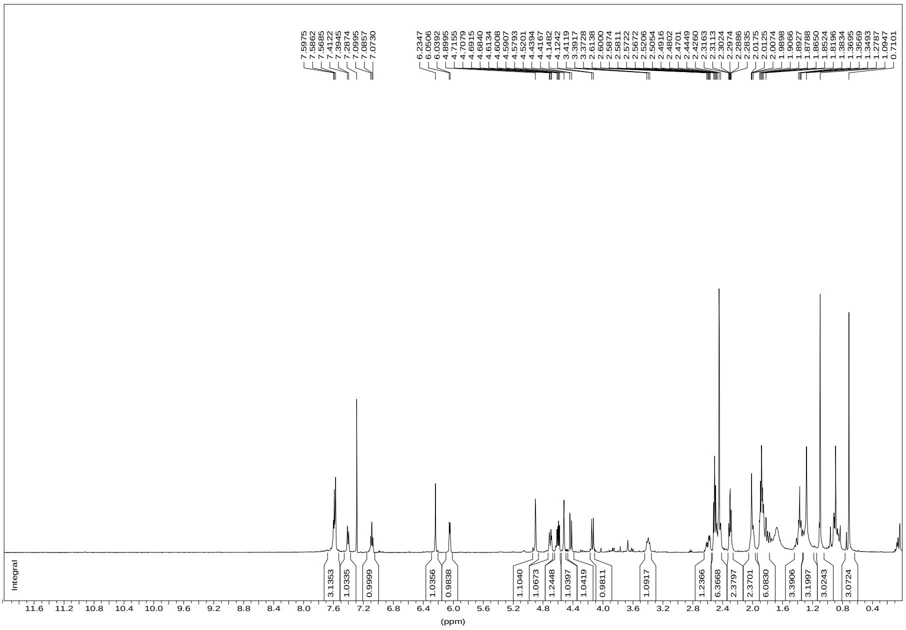

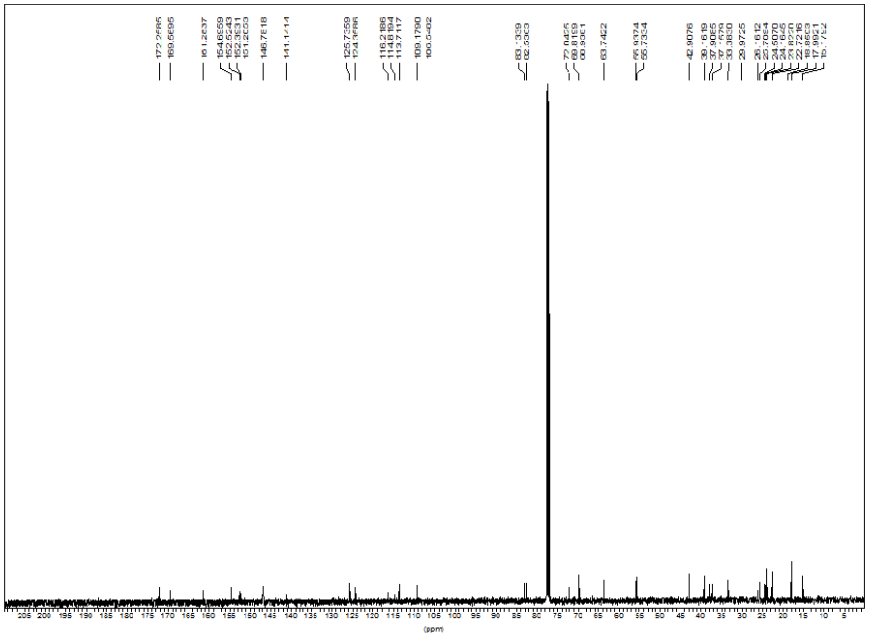

1 = AP1_A549_01
2 = AP1_A549_02
3 = AP1_A549_03

Triplicated samples of A549 cells labeled with **AP1** (10 uM)

4 = NC_A549_01
5 = NC_A549_02
6 = NC_A549_03

Triplicated samples of A549 cells labeled with the negative probe **NC** (10 uM)

Accession	GS	Name	Pro_Mass	1_prot_score	2_prot_score	3_prot_score	4_prot_score	5_prot_score	6_prot_score	1_emPAI	2_emPAI	3_emPAI	4_emPAI	5_emPAI	6_emPAI
P43490	NAMPT	NAMPT_HUMAN Nicotir	55772	270	253	309	-1	-1	-1	0.58	0.68	0.78	-1	-1	-1
P30837	ALDH1B1	AL1B1_HUMAN Aldehyd	57626	199	261	197	-1	-1	-1	0.32	0.4	0.32	-1	-1	-1
Q96D53	ADCK4	ADCK4_HUMAN Unchar	60773	269	195	198	-1	-1	-1	0.37	0.3	0.23	-1	-1	-1
O94919	ENDOD1	ENDD1_HUMAN Endonu	55723	259	236	240	-1	-1	-1	0.33	0.26	0.33	-1	-1	-1
P00390	GSR	GSHR_HUMAN Glutathic	56791	226	231	184	-1	-1	-1	0.25	0.33	0.33	-1	-1	-1
Q9HBI6	CYP4F11	CP4FB_HUMAN Cytochr	60848	116	129	139	-1	-1	-1	0.23	0.17	0.23	-1	-1	-1
E7EUC7	UGP2	E7EUC7_HUMAN UTP--g	57940	62	58	50	-1	-1	-1	0.18	0.12	0.12	-1	-1	-1
M0QWZ7	SARS2	M0QWZ7_HUMAN Serir	58602	141	50	106	-1	-1	-1	0.18	0.06	0.18	-1	-1	-1
Q7L5N7	LPCAT2	PCAT2_HUMAN Lysopho	60797	68	42	30	-1	-1	-1	0.11	0.05	0.05	-1	-1	-1
Q6STE5	SMARCD3	SMRD3_HUMAN SWI/SN	55210	59	81	43	-1	-1	-1	0.06	0.06	0.06	-1	-1	-1
Q07973	CYP24A1	CP24A_HUMAN 1,25-dih	59408	63	51	64	-1	-1	-1	0.06	0.06	0.06	-1	-1	-1
Q16678	CYP1B1	CP1B1_HUMAN Cytochr	61263	75	35	71	-1	-1	-1	0.05	0.05	0.05	-1	-1	-1
Q9P0J1	PDP1	PDP1_HUMAN [Pyruvate	61586	58	59	52	-1	-1	-1	0.05	0.05	0.05	-1	-1	-1
B4DXT0	CCDC151	B4DXT0_HUMAN Coiled-	63926	34	34	34	-1	-1	-1	0.05	0.05	0.05	-1	-1	-1

(-1 = no detected MS signal)
